# Design, synthesis and cholinesterase inhibitory properties of new oxazole benzylamine derivatives

**DOI:** 10.1080/14756366.2019.1707197

**Published:** 2020-01-03

**Authors:** Ivana Šagud, Nikolina Maček Hrvat, Ana Grgičević, Tena Čadež, Josipa Hodak, Milena Dragojević, Kornelija Lasić, Zrinka Kovarik, Irena Škorić

**Affiliations:** a Department of Organic Chemistry, Faculty of Chemical Engineering and Technology, University of Zagreb, Zagreb, Croatia; b Institute for Medical Research and Occupational Health, Biochemistry and Analytic Organic Chemistry Unit, Zagreb, Croatia; c TEVA, Pliva Tapi R&D, Zagreb, Croatia

**Keywords:** Arylethenyl-oxazole, benzylamine, cholinesterase, electrocyclization, naphthoxazole, synthesis

## Abstract

The enzymes acetylcholinesterase (AChE) and butyrylcholinesterase (BChE) are primary targets in attenuating the symptoms of neurodegenerative diseases. Their inhibition results in elevated concentrations of the neurotransmitter acetylcholine which supports communication among nerve cells. It was previously shown for *trans-*4/5-arylethenyloxazole compounds to have moderate AChE and BChE inhibitory properties. A preliminary docking study showed that elongating oxazole molecules and adding a new NH group could make them more prone to bind to the active site of both enzymes. Therefore, new *trans-*amino-4-/5-arylethenyl-oxazoles were designed and synthesised by the Buchwald-Hartwig amination of a previously synthesised *trans-*chloro-arylethenyloxazole derivative. Additionally, naphthoxazole benzylamine photoproducts were obtained by efficient photochemical electrocyclization reaction. Novel compounds were tested as inhibitors of both AChE and BChE. All of the compounds exhibited binding preference for BChE over AChE, especially for *trans-*amino-4-/5-arylethenyl-oxazole derivatives which inhibited BChE potently (IC_50_ in µM range) and AChE poorly (IC_50_≫100 µM). Therefore, due to the selectivity of all of the tested compounds for binding to BChE, these compounds could be applied for further development of cholinesterase selective inhibitors.

**HIGHLIGHTS**
- Series of oxazole benzylamines were designed and synthesised
- The tested compounds showed binding selectivity for BChE
- Naphthoxazoles were more potent AChE inhibitors

## 1. Introduction

Acetylcholinesterase (AChE, EC 3.1.1.7) and butyrylcholinesterase (BChE, EC 3.1.1.8) are two related enzymes present in vertebrates and plants. In humans, these enzymes are products of different genes but share about 54% of their amino acid sequence^1^. The major difference in their active site are the 14 aromatic amino acid residues in AChE which correspond to 8 aromatic and 6 aliphatic residues in BChE^2^. This enables BChE to hydrolyse larger substrates and ligands than AChE and is accountable for the binding selectivity of cholinesterases^3–6^.

AChE has an essential physiological role in the body as it controls the transmission of nerve impulses in the cholinergic synapses of the central and peripheral nervous system by hydrolysis of the neurotransmitter acetylcholine. It also has a role in neuritogenesis, cell adhesion, proliferation and cell interactions, synaptogenesis, dopamine neuronal activation, the formation of amyloid fibres characteristic for Alzheimer's disease, haematopoiesis and thrombopoiesis^7–9^. The role of BChE is not physiologically essential but it could be assigned to the detoxification of xenobiotics (organophosphates and carbamate pesticides, cocaine, aspirin, succinyldicholine, etc.) and bioactivation of drugs (bambuterol, heroin, etc.)^10^^,^^11^. Also, BChE serves as a co-regulator of cholinergic neurotransmission and is capable of catalysing the hydrolysis of acetylcholine^12^. It was found that high BChE levels are associated with neuritic plaques and neurofibrillary tangles, the neuropathologic hallmarks of Alzheimer’s disease (AD)^13^^,^^14^. Therefore, both cholinesterases are pharmacologically relevant targets in neurodegenerative disorders, and today’s treatment includes cholinesterase inhibitors like donepezil, galantamine, physostigmine, rivastigmine, ect.^15^. Many other compounds acting as inhibitors of cholinesterase are therefore considered as potential AD therapeutics^16–18^.

Recently we have shown that 4/5-arylethenyloxazoles possess a moderate potency to inhibit AChE and BChE^19^. In this study, we designed new *trans-*amino-5-arylethenyl-oxazole derivatives where the oxazole molecule has an NH group on one of the substituents. For the synthesis of styryl-oxazoles, the Van Leusen reaction was utilised. Styryl-oxazole that has chlorine as a substituent was then *N*-alkylated by Buchwald-Hartwig type reaction to give oxazole benzylamines which were tested as cholinesterase inhibitors. All of the styryl-oxazole amines were also photochemically cyclized to give naphthoxazole benzylamines^20^. Naphthoxazoles synthesised by this manner were also tested. This gave a wide range of molecules with either oxazole or the naphthoxazole moiety to evaluate their impact on the cholinesterase inhibitory activity.

## 2. Results and discussion

### 2.1. Synthesis and photochemistry of novel oxazole benzylamines

**Scheme 1.**
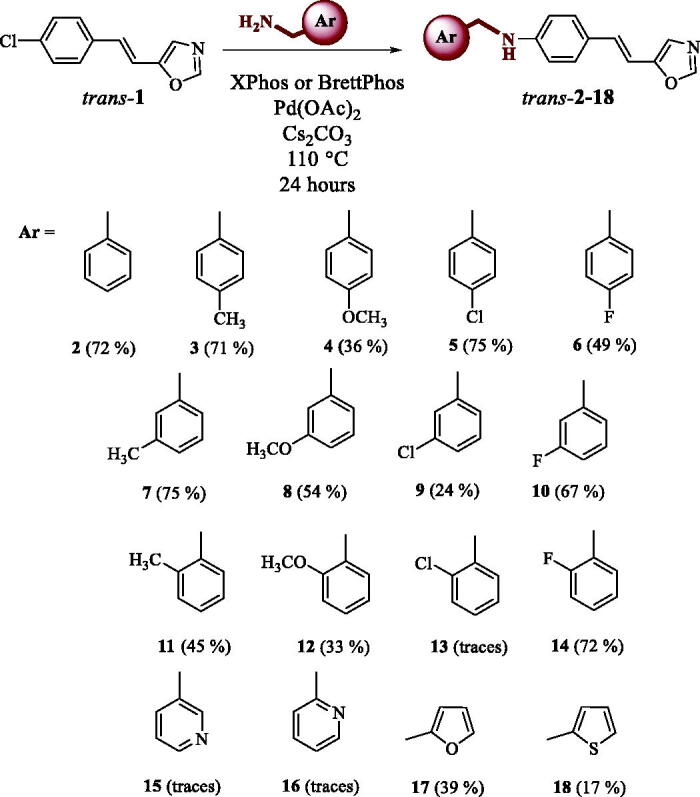
Synthesis of targeted compounds *trans-***2–18** by Buchwald-Hartwig reaction.

Using the reaction of *N*-alkylation on the previously synthesised *trans-*chloro-arylethenyloxazole **1**^20^, new *trans-*amino-5-arylethenyl-oxazole derivatives *trans-***2–18** were synthesised (Scheme 1) with an aim to add a new functional group at the end of the oxazole derivative that resembles acetylcholine, the substrate of cholinesterase. The Buchwald-Hartwig reaction^21^ was utilised with two catalysts and the reaction was optimised for best conditions to enhance the yield. Change of base was crucial for the optimisation of this reaction. Sodium *tert*-butoxide was previously used as a base but the dehalogenation of the starting material was observed. Caesium carbonate improved yield and conversion. Temperature, solvent and catalyst used were independently varied to give the best conversion. The best conditions found are given in Scheme 1. The catalysed *N*-alkylation reaction is a complex coupling reaction and it gave a vast array of yields. Some of the substrates were optimised to excellent yields, while in the example of others only moderate to low yields were obtained. There is still some room for optimisation in the future with additional catalysts but at this time this was sufficient.

**Scheme 2.**
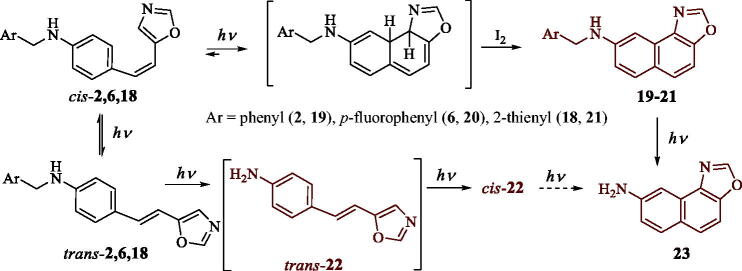
Photochemical reactivity of amino-5-arylethenyl-oxazoles *trans*-**2,6,18** into naphtho[1,2-*d*]oxazoles, **19**,**20** and **21**, respectively.

A vast number of new compounds was synthesised and spectroscopically characterised (See experimental and Supplementary Figures S1–S19). Compounds with a pyridine ring **15** and **16** and that bearing 2-chlorophenyl substituent **13** were synthesised only in trace amounts and these compounds were not further investigated. Pyridine derivatives **15** and **16** could not be obtained probably because of the influence of basicity of the heteroaromatic ring containing nitrogen on the complex reaction steps of Buchwald-Hartwig amination reaction. Only some of the compounds (*trans*-**2**, *trans*-**6** and *trans*-**18**) were successfully photochemically cyclized into novel polycyclic derivatives **19–21** (Scheme 2). Other starting amines did not react in the electrocyclization reaction and remained unreacted in the reaction solution, some of them as mixture of configurational isomers. The photochemically reactive anilines showed *cis*-*trans* photoisomerization during the photoreaction and as the consequence of that gave photocyclization products **19–21** as only *cis*-configuration is suitable for electrocyclization. Only the *cis*-isomer of the amine **18** was isolated from the photomixture after the cyclisation reaction, and spectroscopically characterised. It does not mean that the photostationary state is not established in the photomixture in the case of other amines, but without further electrocyclization reaction. During the photocyclization of 2-thienyl (*trans-***18**) derivative, the competitive cleavage of the heteroaromatic moiety occurred resulting in the isolation of the *cis*-**22** and its electrocyclization product **23**. The same products were seen also in the ^1^H NMR spectrum after photoreaction of *trans*-**17**. The difference between these two heteroaromatic amines is that *trans*-**17** does not cyclize to the corresponding electrocyclization product and gave these products only in traces. The formation of the same product **23** can be also explained as the consequence of the cleavage of the heteroaromatic moiety from **21** (Scheme 2) and this pathway of formation of **23** cannot be excluded as both pathways can occur as competitive processes in the same photoreaction.

**Figure 1.**
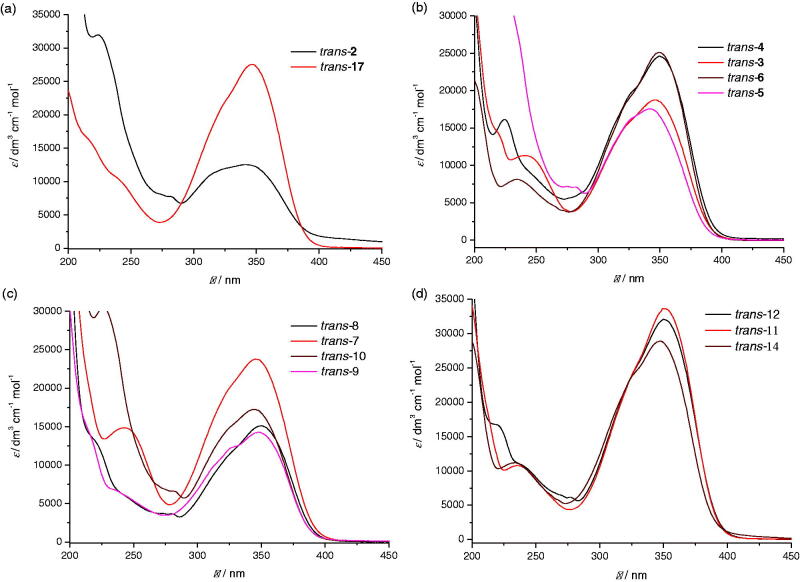
UV spectra of compounds *trans-***2** and *trans-***17** (a), *para*-substituted synthesised compounds *trans-***3–6** (b), *meta*-substituted synthesised compounds *trans-***7–10** (c) and *ortho*-substituted synthesised compounds *trans-***11**, *trans-***12** and *trans-***14** (d).

All compounds were completely spectroscopically characterised. On Figure 1, UV spectra are given as they are used in the determination of wavelength in cyclisation reactions. Absorption maxima of all of the starting *trans*-isomers of compounds **2–18** are in the area between 340 nm and 350 nm and that is the reason why irradiation at 350 nm wavelength was used for cyclisation.

**Figure 2.**
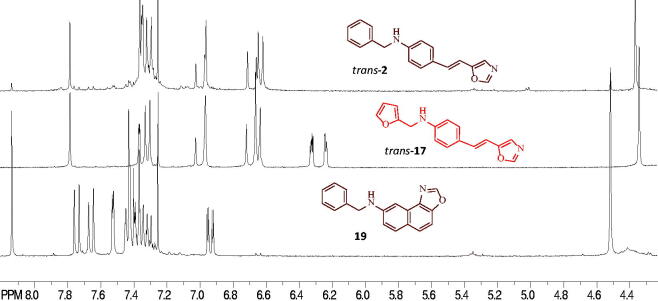
Partial ^1^H NMR spectra of starting amines *trans-***2** and *trans-***17** and of the photocyclization product **19**.

All isolated compounds exhibited in the ^1^H NMR spectrum a singlet in the range of 7.78–7.81 ppm, which was attributed to the proton on the position 2 of the oxazole ring due to the influence of nitrogen and oxygen found in its immediate vicinity unshaded and shifted to a lower field (Figure 2 and Supplementary Figures S1–S19). The protons located at position 4 of the oxazole ring showed a singlet in the range of 6.95–6.99 ppm, the ethylenic protons are visible as doublets in the range of 6.99–7.08 ppm with coupling constants between 16 Hz and 17 Hz, characteristic for *trans*-isomers. For compounds *trans-***17** and *trans-***18**, the characteristic signals for the furan or thiophene ring are also visible with characteristic coupling constants (See experimental and Supplementary Figure S16). In the spectra of electrocyclization products **19–21,** two new doublets with *cis* coupling constants appeared, characteristic for the central ring of the cyclized naphthoxazole. The structure and purity of the synthesised amines were also confirmed by ^13 ^C NMR and two-dimensional NMR techniques as well as HRMS analyses (See experimental and Supplementary Figures S1–S19).

### 2.2. Inhibition of cholinesterases by novel oxazole benzylamines

The eleven new synthetised *trans-*amino-5-arylethenyl-oxazole derivatives (*trans*-**2**-*trans*-**12**, and *trans*-**17**) were tested in a wide concentration range as BChE inhibitors to evaluate the inhibitor concentration that inhibits 50% of enzyme activity (IC_50_), presented in Table 1. The most potent BChE inhibitors were compounds *trans-***12**, *trans-***10** and *trans-***8** with an IC_50_ of about 30 µM. BChE had the lowest binding affinity for compound *trans-***11** which was 5.5-fold lower than the most potent inhibitor *trans-***12**. It is interesting to note that the binding affinity of BChE for compounds *trans-***12**, *trans-***10** and *trans-***8** was similar as reported for cholinesterase inhibitors BW284C51, huperzine or rivastigmine (IC_50_ 30 – 54 µM)^22^.

**Table 1.** Inhibition of BChE and AChE by tested *trans-*amino-5-arylethenyl-oxazole derivatives (*trans*-**2**-*trans*-**17**), naphtho[1,2-*d*]oxazoles (**19**–**21** and **23),** and amino-4/5-arylethenyl-oxazoles (*cis*-**18** and *cis*-**22)**, expressed as IC_50_ ± SE.

	IC_50_ (µM)	
Compound (aromatic/heteroaromatic substitution)	BChE	AChE
*trans-***2** (phenyl)	120 ± 22	≫100
*trans-***3** (*p*-methylphenyl)	120 ± 19	≫100
*trans-***4** (*p*-methoxyphenyl)	110 ± 15	≫100
*trans-***5** (*p*-chlorophenyl)	87 ± 12	≫100
*trans-***6** (*p*-fluorophenyl)	80 ± 10	≫100
*trans-***7** (*m*-methylphenyl)	130 ± 26	≫100
*trans-***8** (*m*-methoxyphenyl)	36 ± 4.4	≫100
*trans-***10** (*m*-fluorophenyl)	32 ± 5.2	≫100
*trans-***11** (*o*-methylphenyl)	160 ± 35	≫100
*trans-***12** (*o*-methoxyphenyl)	28 ± 5.2	≫100
*trans-***17** (2-furyl)	65 ± 10	≫100
**19** (phenyl)	140 ± 24	68 ± 25
**20** (*p*-fluorophenyl)	12 ± 1.3	45 ± 17
**21** (2-thienyl)	35 ± 7.1	51 ± 20
**23**	1000 ± 650	120 ± 46
*cis*-**22**	110 ± 58	190 ± 100
*cis*-**18** (*p*-fluorophenyl)	5.7 ± 0.8	160 ± 100
Ethopropazine	0.046 ± 0.0037	73 ± 8.3

Generally, although these compounds, with the exception of compound *trans-***17**, systematically differ only by their substituent on the benzyl group and its substituent position (*ortho-, meta-, para-*), this structural variation cannot be easily related to IC_50_ values. The most potent inhibitors, compounds *trans-***12**, *trans-***10** and *trans-***8** have *ortho*-methoxy, *meta*-fluoro and *meta*-methoxy substituents at the phenyl rings, respectively. Moreover, *ortho*- (*trans-***12**) and *meta*- (*trans-***8**) analogues had about a 3 times lower IC_50_ than their *para*-methoxy analogue (*trans-***4**). Similarly, the *meta-*fluoro substituted compound, *trans-***10**, exhibited a 2.5 times lower IC_50_ than its *para*-fluoro analogue, *trans-***6**. In case of the methyl substituent, the position of the substituent was not relevant because all three compounds (*trans-***3**, *trans-***7** and *trans-***11**) had a similar IC_50_ and were the weakest inhibitors among the tested compounds (Table 1). Nevertheless, the *para*-substitution and methyl-substitution led to inactive compounds, while the *ortho*/*meta*-methoxy and the *meta*-fluoro were active derivatives. It is not surprising that the activity was noticeably different between the most potent (*trans-***12**) and the weakest inhibitor (*trans-***11**) differed in the methoxy and methyl substituent at position 2, respectively.

Eleven *trans*-amino-5-arylethenyl-oxazole derivatives inhibited maximally 20% of AChE activity (Supplementary Figure S20) and IC_50_ values were not determined. Since higher concentrations than 100 µM could not be used due to AChE inhibition by solvent DMSO^23^, the IC_50_ values for AChE were presumably much higher than 100 µM.

Four of the polycyclic naphtho[1,2-*d*]oxazoles compounds (**19**, **20**, **21**, **23**) and two isolated *cis-*isomers of amino-5-arylethenyl-oxazole derivatives (*cis-***18**, *cis-***22**) were also tested as potential inhibitors of cholinesterases. All compounds, except **23**, inhibited both enzymes more than 50% with concentrations in µM range and the evaluated IC_50_ values are given in Table 1. The IC_50_ for BChE and compound *cis-***18** was the lowest IC_50_ value evaluated in this study. *cis*-**18** was about 5 times more potent inhibitor of BChE than amino-5-arylethenyl-oxazoles *trans*-**12**, *trans*-**10** and *trans*-**8** (Table 1). It is also interesting to note that *cis-***18** and *cis-***22** exhibited a higher inhibition effect for BChE than their electrocyclic products **21** and **23**, respectively, while polycyclic derivative **20** had about 8-fold higher potency for BChE than its counterpart *trans*-**6** (Table 1).

In the case of AChE, it seems that the electrocyclization and *trans-cis* isomerisation of amino-5-arylethenyl-oxazole derivatives enabled additional interactions in the active site improving the inhibition potency. The electrocyclization of *cis*-**18** and *cis*-**6** resulted in the thienyl-naphtho[1,2-*d*]oxazole, **21**, and *p*-fluorophenyl-naphtho[1,2-*d*]oxazole, **20**, respectively. Both compounds **21** and **20** are potent inhibitors of AChE. The naphtho[1,2-*d*]oxazole without aminoalkyl substituent **23** was the poorest inhibitor out of the six tested compounds (Table 1). Again, except for **23,** there was a slight binding preference of BChE. Therefore, generally, the obtained results indicate that the tested oxazole amines as well as naphtho[1,2-*d*]oxazole derivatives may be classified as selective inhibitors of BChE.

## 3. Conclusions

New amino-5-arylethenyl-oxazoles *trans*-**2–18,** and *cis*-**18** and *cis*-**22,** as well as naphthoxazole benzylamines **19–23** were successfully synthesised using the reaction of *N*-alkylation on previously synthesised *trans*-chloro-arylethenyl-oxazole **1**. The IC_50_ values evaluated for BChE classified the tested compounds as moderate BChE inhibitors. Naphtho[1,2-*d*]oxazoles showed to be more potent AChE inhibitors than the *trans*-amino-5-arylethenyl-oxazole derivatives, which inhibited 20% of AChE activity at most at the highest concentration possible to test. Due to the selectivity of tested oxazole benzylamines for binding to BChE, the scaffold of these compounds could be used for further development of cholinesterase selective inhibitors.

## 4. Experimental section

### 4.1. Chemistry

#### 4.1.1. General procedures

Reactions that required the use of anhydrous, inert atmosphere techniques were carried out under an atmosphere of nitrogen. Petroleum ether, bp 40–60 °C, was used. Solvents were purified by distillation. Column chromatography was carried out on columns with silica gel (Fluka 0.063–0.2 nm and Fluka 60 Å, technical grade). TLC was carried out using plates coated with silica gel (0.2 mm, 0.5 mm, 1.0 mm, Kieselgel 60 F_254_). Organic layers were routinely dried with anhydrous MgSO_4_ and evaporated using a rotary evaporator. ^1^H and ^13 ^C NMR spectra were recorded on a spectrometer at 300 and 600 MHz. All NMR spectra were measured in CDCl_3_ using tetramethylsilane as reference. The assignment of signals was based on 2 D-CH correlation and 2 D-HH-COSY experiments. The following abbreviations are used: s, singlet; d, doublet; t, triplet; q, quartette, dd, doublet of doublets; m, multiplet and br, broad. UV spectra were measured on a UV/VIS spectrophotometer. IR spectra were recorded on a FTIR. Mass spectra were obtained on a GC-MS system. Melting points were obtained using a microscope equipped apparatus and are uncorrected. HRMS analyses were carried out on a mass spectrometer. The LC-MS system consisted of an Agilent 1290 LC coupled with an Agilent 6550 iFunnel quadrupole time-of-flight mass spectrometer (Agilent Technologies, Santa Clara, CA, USA). The LC-MS system equipped with a quaternary gradient pump, temperature-controlled column compartment, refrigerated autosampler component, diode array detector (DAD) and MS with electrospray ionisation were used for the identification. Chromatographic separations were performed using Acquity UPLC BEH C18 column, 50 × 2.1 mm, 1.7 µm (Agilent Technologies, Santa Clara, CA, USA). Gradient elution with mobile phase containing solvent A (0.1% formic acid) and solvent B (acetonitrile) was used. The mobile-phase flow rate was 0.4 mL/min and the column temperature was maintained at 50 ± 1 °C. Substances were analysed in positive electrospray ionisation mode. Nitrogen was a nebuliser and curtain gas. The capillary voltage was 3500 V. Gas temperature was 200 °C, gas flow was 14 L/min, nebuliser was 35 psi, sheath gas temperature was 350 °C and sheath gas flow was 11 L/min. Data acquisition and processing were performed on a MassHunter Data Acquisition for Q-TOF B.06.01 (B6157) software (Agilent Technologies). Irradiation experiments were performed in a closed quartz vessel in toluene solution in a photochemical reactor equipped with 360 nm lamps. The solvents were removed on the rotatory evaporator under reduced pressure in a ventilated hood.

#### 4.1.2. Synthesis of (E)-5–(4-chlorostyryl)oxazole (trans-1)

Compound *trans***-1** was synthesised from (*E*)-3–(4-chlorophenyl)acryladehyde (6.00 mmol, 1 eq) by Van Leusen reaction^,^^24^ with tosylmethylisocyanide (TosMIC) (5.76 mmol, 0.96 eq) reagent and potassium carbonate as base (5.79 mmol, 0.96 eq) in methanol. (*E*)-5–(4-chlorostyryl)oxazole (*trans***-1**) of compound^17^ was isolated as yellow powder (0.950 g; 77.24%): mp 78–81 °C; Rf (PE/E, 20%) = 0.59; UV (EtOH) *λ*_max_/nm (*ε*/dm^3^mol^−1 ^cm^−1^): 299 (27977), 312 (29351), 326 (20895); IR *ν*_max_/cm^−1^ (NaCl): 1697, 1610, 1491, 1089; ^1^H NMR (CDCl_3_, 600 MHz): *δ*/ppm 7.84 (s, 1H, H-2), 7.40 (dd, *J*_ar_= 8.5 Hz, *J*_ar_= 6.6 Hz, 2H, H-ar), 7.33 (dd, *J*_ar_= 8.5 Hz, *J*_ar_= 6.6 Hz, 2H, H-ar), 7.08 (s, 1H, H-4), 7.04 (d, *J*_et_ = 16.2 Hz, 1H, H-et), 6.88 (d, *J*_et_ = 16.2 Hz, 1H, H-et); ^13 ^C NMR (CDCl_3_, 150 MHz): *δ*/ppm 150.63 (d), 149.23 (s), 147.54 (s), 137.03 (s), 132.58 (s), 129.92 (d), 128.33 (d), 128.12 (d), 127.52 (d), 125.52 (d), 122.11 (d), 112.48 (d), 108.52 (d), 46.88 (t); ^13 ^C NMR (CDCl_3_, 150 MHz): *δ*/ppm 149.9 (d, C-2), 149.7 (s), 134.2 (s), 133.5 (s), 128.5 (d), 128.4 (d), 127.2 (d), 124.0 (d), 112.9 (d); MS *m/z* (EI) = 205 (100, M^+^); HRMS(Q-TOF) for C_11_H_8_ClNO: (M + H)^+^_calcd_ = 206.0294, (M + H)^+^_found_ = 206.0369.

#### 4.1.3. Synthesis of new (E)-N-benzyl-4–(2-(oxazol-5-yl)vinyl)anilines

##### 4.1.3.1. Synthesis with BrettPhos

BrettPhos (0.024 mmol 0.1 eq), Pd(OAc)_2_ (0.012 mmol, 0.05 eq) were suspended in 2 mL of dioxane with 0.01 mL water and heated to 120 °C. (*E*)-5–(4-chlorostyryl)oxazole (0.243 mmol, 1 eq) Cs_2_CO_3_ (0.365 mmol, 1,5 eq) and different benzyl-amines were added (0.486 mmol, 2 eq). The reaction mixture was heated in a pressure tube to 110 °C for 24 h. Solvent was evaporated under pressure and the compound purified by column chromatography on silicagel using petroleumether/dichloromethane (20–100%) as eluent.

##### 4.1.3.2. Synthesis with XPhos

(*E*)-5–(4-chlorostyryl)oxazole (0.243 mmol, 1 eq), XPhos (0.049 mmol, 0.2 eq), Pd(OAc)_2_ (0.012 mmol, 0.05 eq) and Cs_2_CO_3_ (0.365 mmol, 1.5 eq) were dissolved in 2 mL of dioxane and benzyl-amines (0.486 mmol, 2 eq) were added. The reaction mixture was purged with argon and heated to 110 °C in a pressure tube for 24 h. Solvent was evaporated under pressure and the compound purified by column chromatography on silica gel using petroleumether/dichloromethane (20–100%) as eluent.

(*E*)-*N*-benzyl-4–(2-(oxazol-5-yl)vinyl)aniline (*trans***-2**) was isolated (0.097 g (72.22%)) as yellow powder: mp 112–118 °C; Rf (DCM, 100%) = 0.32; UV (EtOH) *λ*_max_/nm (*ε*/dm^3^mol^−1 ^cm^−1^): 224 (32046), 319 (Sh, 11687), 343 (12469); IR *ν*_max_/cm^−1^ (NaCl): 3421, 2924, 1748, 1607, 1524, 1490, 1453, 955, 817, 744, 638; ^1^H NMR (CDCl_3_, 300 MHz) *δ*/ppm: 7.79 (s, 1H, H-Ox_2_), 7.37–7.28 (m, 8H, H-Ar, H-NH), 7.00 (d, 1H, *J*_Et1,Et2_ = 16.07 Hz, H-Et_1_), 6.97 (s, 1H, H-Ox_4_), 6.69 (d, 1H, *J*_Et2,Et1_ = 16.42 Hz, H-Et_2_), 6.63 (d, 2H, *J*_Ar2a,Ar1a_ = 8.74 Hz, H-Ar_2a_), 4.37 (s, 2H, H-CH_2_); ^13 ^C NMR (CDCl_3_, 75 MHz): *δ*/ppm 149.22 (s), 147.88 (s), 138.46 (s), 130.04 (d), 128.21 (d), 127.51 (d), 126.97 (d), 126.88 (d), 125.27 (s), 122.02 (d), 112.38 (d), 108.33 (d), 47.59 (t); HRMS(Q-TOF) for C_18_H_16_N_2_O: (M + H)^+^_calcd_ = 277.1335, (M + H)^+^_found_ = 277.1325.

(*E*)-*N*-(4-methylbenzyl)-4–(2-(oxazol-5-yl)vinyl)aniline (*trans***-3**) was isolated (0.099 g (70.92%)) as yellow powder mp 128–131 °C; Rf (DCM, 100%) = 0.43; UV (EtOH) *λ*_max_/nm (*ε*/dm^3^mol^−1 ^cm^−1^): 242 (11249), 325 (Sh,15804), 346 (18719); IR *ν*_max_/cm^−1^ (NaCl): 3416, 2925, 1612, 1577, 1558, 1519, 1476, 953, 820, 637; ^1^H NMR (CDCl_3_, 300 MHz) *δ*/ppm: 7.81 (s, 1H,H-Ox_2_), 7.32 (d, 2H, *J*_Ar1aAar2a_ = 8.58 Hz, H-Ar_1a_), 7.27 (d, 2H, *J*_Ar3b,Ar4b_ = 7.82 Hz, H-Ar_3b_), 7.18 (d, 2H, *J*_Ar4b,Ar3b_ = 7.82 Hz, H-Ar_4_), 7.02 (d, 1H, *J*_Et1,Et2_ = 16.65 Hz, H-Et_1_), 6.98 (s, 1H, H-Ox_4_), 6.70 (d, 1H, *J*_Et2,Et1_ = 16.40 Hz, H-Et_2_), 6.63 (d, 2H, *J*_Ar2a,Ar1a_ = 8.58 Hz, H-Ar_2a_), 4.34 (s, 2H, H-CH_2_), 4.21 (s, 1H, H-NH), 2.36 (s, 3H, H-CH_3_); ^13 ^C NMR (CDCl_3_, 75 MHz): *δ*/ppm 147.95 (s), 136.56 (s), 135.38 (s), 130.10 (d), 128.88 (d), 127.51 (d), 126.93 (d), 125.16 (s), 122.01 (d), 112.36 (d), 108.25 (d), 47.34 (q), 20.58 (t); HRMS(Q-TOF) for C_19_H_18_N_2_O: (M + H)^+^_calcd_ = 291.1492, (M + H)^+^_found_ = 291.1487.

(*E*)-*N*-(4-methoxybenzyl)-4–(2-(oxazol-5-yl)vinyl)aniline (*trans***-4**) was isolated (0.054 g (36.29%)) as yellow powder mp 153–159 °C; Rf (DCM, 100%) = 0.38; UV (EtOH) *λ*_max_/nm (*ε*/dm^3^mol^−1 ^cm^−1^): 224 (16148), 328 (Sh, 19991), 350 (24578); IR *ν*_max_/cm^−1^ (NaCl): 3376, 2925, 1601, 1514, 1468, 952, 822, 638; ^1^H NMR (CDCl_3_, 300 MHz) *δ*/ppm: 7,81 (s, 1H, H-Ox_2_), 7.34–7.29 (m, 4H, H-Ar), 7.02 (d, 1H, *J*_Et1,Et2_ = 16.51 Hz, H-Et_1_), 6.98 (s, 1H, H-Ox_4_), 6.90 (d, 2H, *J*_Ar1a,Ar2a_ = 8.77 Hz, H-Ar_1a_), 6.70 (d, 1H, *J*_Et2,Et1_ = 16.26 Hz, H-Et_2_), 0.63 (d, 2H, *J*_Ar2a,Ar1a_ = 8.51 Hz, H-Ar_2a_), 4.31 (s, 1H, H-CH_2_), 4.17 (s, 1H, H-NH), 3.82 (s, 3H, H-OCH_3_); ^13 ^C NMR (CDCl_3_, 75 MHz): *δ*/ppm 158.50 (s), 147.93 (s), 130.43 (s), 130.07 (d), 128.24 (d), 127.17 (s), 125.17 (s), 121.99 (d), 113.62 (d), 112.36 (d), 108.26 (d), 54.80 (q), 47.06 (t); HRMS(Q-TOF) for C_19_H_18_N_2_O_2_: (M + H)^+^_calcd_ = 307.1441, (M + H)^+^_found_ = 307.1439.

(*E*)-*N*-(4-chlorobenzyl)-4–(2-(oxazol-5-yl)vinyl)aniline (*trans***-5**) was isolated (0.114 g (75.49%)) as yellow powder mp 120–125 °C; Rf (DCM, 100%) = 0.43; UV (EtOH) *λ*_max_/nm (*ε*/dm^3^mol^−1 ^cm^−1^): 242 (14854), 325 (Sh, 20225), 346 (23776); IR *ν*_max_/cm^−1^ (NaCl): 3325, 2924, 1605, 1520, 1473, 953, 818, 638; ^1^H NMR (CDCl_3_, 300 MHz) *δ*/ppm: 7.81 (s, 1H, H-Ox_2_), 7.33–7.28 (m, 6H, H-Ar), 7.01 (d, 1H, *J*_Et1,Et2_ = 16.15 Hz, H-Et_1_), 6.99 (s, 1H, H-Ox_4_), 6.70 (d, 1H, *J*_Et2,Et1_ = 16.15 Hz, H-Et_2_), 6.60 (d, 2H, *J*_Ar2a,Ar1a_ = 8.39 Hz, H-Ar_2_), 4.36 (s, 2H, H-CH_2_), 4.28 (s, 1H, H-NH); ^13 ^C NMR (CDCl_3_, 75 MHz): *δ*/ppm 150.63 (d), 149.23 (s), 147.54 (s), 137.03 (s), 132.58 (s), 129.92 (d), 128.33 (d), 128.12 (d), 127.52 (d), 125.52 (s), 122.11 (d), 112.48 (d), 108.52 (d), 46.88 (t); HRMS(Q-TOF) for C_18_H_15_ClN_2_O: (M + H)^+^_calcd_ = 311.0946, (M + H)^+^_found_ = 311.0938.

(*E*)-*N*-(4-fluorobenzyl)-4–(2-(oxazol-5-yl)vinyl)aniline (*trans***-6**) was isolated (0.070 g (48.95%)) as yellow powder mp 137–142 °C; Rf (DCM, 100%) = 0.43; UV (EtOH) *λ*_max_/nm (*ε*/dm^3^mol^−1 ^cm^−1^): 234 (8159), 326 (Sh, 19305), 349 (25076); IR *ν*_max_/cm^−1^ (NaCl): 3325, 2925, 1606, 1520, 1509, 1470, 953, 818, 638; ^1^H NMR (CDCl_3_, 300 MHz) *δ*/ppm: 7.81 (s, 1H, H-Ox_2_), 7.36 (d, 2H, *J*_Ar1a,Ar2a_ = 8.56 Hz, H-Ar_1a_), 7.34 (d, 2H, *J*_Ar4b,Ar3b_ = 8.83 Hz, H-Ar_4b_), 7.05 (d, 1H, *J*_Et1,Et2_ = 16.86 Hz, H-Et_1_), 7.06 (d, 2H, *J*_Ar3b,Ar4b_ = 8.83 Hz, H-Ar_3_), 6.99 (s, 1H, H-Ox_4_), 6.71 (d, 1H, *J*_Et2,Et1_ = 16.32 Hz, H-Et_2_), 6.62 (d, 2H, *J*_Ar2a,Ar1a_ = 8.56 Hz, H-Ar_2_), 4.35 (s, 2H, H-CH_2_), 4.24 (s, 1H, H-NH); ^13 ^C NMR (CDCl_3_, 75 MHz): *δ*/ppm 162.44 (s), 150.67 (s), 149.23 (s), 147.66 (s), 134.16 (s), 129.95 (d), 128.48 (d), 128.42 (d), 127.52 (d), 125.43 (d), 122.08 (d), 115.11 (d), 114.97 (d), 112.42 (d), 108.46 (d), 46.88 t); HRMS(Q-TOF) for C_18_H_15_FN_2_O: (M + H)^+^_calcd_ = 295.1241, (M + H)^+^_found_ = 295.1234.

(*E*)-*N*-(3-methylbenzyl)-4–(2-(oxazol-5-yl)vinyl)aniline (*trans***-7**) was isolated (0.105 g (75.18%)) as yellow powder mp 75–80 °C; Rf (DCM, 100%) = 0.31; UV (EtOH) *λ*_max_/nm (*ε*/dm^3^mol^−1 ^cm^−1^): 242 (14732), 325 (Sh, 19824), 346 (23783); IR *ν*_max_/cm^−1^ (NaCl): 3413, 3325, 2921, 1605, 1520, 1489, 1469, 953, 817, 638; ^1^H NMR (CDCl_3_, 300 MHz) *δ*/ppm: 7.81 (s, 1H, H-Ox_2_), 7.34–7.13 (m, 6H, H-Ar), 7.08 (d, 1H, *J*_Et1,Et2_ = 16.54 Hz, H-Et_1_), 6.98 (s, 1H, H-Ox_4_), 6.70 (d, 1H, *J*_Et2,Et1_ = 16.54 Hz, H-Et_2_), 6.63 (d, 2H, *J*_Ar2a,Ar1a_ = 8.14 Hz, H-Ar_2a_), 4.34 (s, 2H, H-CH_2_), 4.23 (s, 1H, H-NH), 2.37 (s, 3H, H-CH_3_); ^13 ^C NMR (CDCl_3_, 75 MHz) *δ*/ppm: 151.23 (d, C-Ox_2_), 149,73 (s), 148,48 (s), 138, 92 (s), 138,42 (s), 130,57 (d, C-Et_1_), 128,63 (d, C-Ar), 128,23 (d, C-Ar), 128,16 (d, C-Ar), 128,04 (d, C-Ar), 125,65 (s), 124,53 (d, C-Ar), 122,52 (d, C-Ox_4_), 112,86 (d, C-Ar_1a_), 108,75 (d, C-Et_2_), 48,09 (t, C-CH_2_), 21,45 (q, C-CH_3_); HRMS(Q-TOF) for C_19_H_18_N_2_O: (M + H)^+^_calcd_ = 291.1492, (M + H)^+^_found_ = 291.1485.

(*E*)-*N*-(3-methoxybenzyl)-4–(2-(oksazol-5-yl)vinyl)aniline (*trans***-8**) was isolated (0.080 g (53.76%)) as yellow oil: R_f_ (DCM, 100%) = 0.27; UV (EtOH) *λ*_max_/nm (*ε*/dm^3^mol^−1 ^cm^−1^): 219 (Sh, 13312), 326 (Sh, 11808), 349 (15095); IR *ν*_max_/cm^−1^ (NaCl): 3307, 2927, 1605, 1522, 1489, 1465, 953, 817, 639; ^1^H NMR (CDCl_3_, 300 MHz) *δ*/ppm: 7.81 (s, 1H, H-Ox_2_), 7.33 (d, 2H, *J*_Ar1a,Ar2a_ = 8.56 Hz, H-Ar_1_), 7.02 (d, 1H, *J*_Et1,Et2_=16.51 Hz, H-Et_1_), 6.98 (s, 1H, H-Ox_4_), 6.97–6.90 (m, 1H, H-Ar_5b_), 6.93 (s, 1H, H-Ar_2b_), 6.84 (d, 2H, *J*_Ar4b,6b,Ar5b_ = 8.00 Hz, H-Ar_4b_, H-Ar_6b_), 6.70 (d, 1H, *J*_Et2,Et1_ = 16.38 Hz, H-Et_2_), 6.63 (d, 2H, *J*_Ar2a,Ar1a_ = 8.38 Hz, H-Ar_2_), 4.36 (s, 2H, H-CH_2_), 4.25 (s, 1H, H-NH), 3.82 (s, 3H, H-OCH_3_); ^13 ^C NMR (CDCl_3_, 75 MHz) *δ*/ppm:133.05 (d), 131.30 (d), 130.05 (d), 129.23 (d), 127.50 (d), 125.47 (d), 125.26 (s), 119.72 (d), 119.13 (d), 112.56 (d), 112.40 (d), 112.19 (d), 108.31 (d), 47.55 (t); HRMS (Q-TOF) for C_19_H_18_N_2_O_2_: (M + H)^+^_calcd_ = 307.1441, (M + H)^+^_found_ = 307.1434.

(*E*)-*N*-(3-chlorobenzyl)-4–(2-(oxazol-5-yl)vinyl)aniline (*trans***-9**) was isolated (0.036 g (23.83%)) as yellow oil: Rf (DCM, 100%) = 0.27; UV (EtOH) *λ*_max_/nm (*ε*/dm^3^mol^−1 ^cm^−1^): 216 (Sh, 13861), 234 (6874), 329 (Sh, 12349), 347 (14260); IR *ν*_max_/cm^−1^ (NaCl): 3325, 2925, 1606, 1520, 1473, 953, 818, 638; ^1^H NMR (CDCl_3_, 300 MHz) *δ*/ppm: 7.81 (s, 1H, H-Ox_2_), 7.41–7.31 (m, 6H, H-Ar), 7.02 (d, 1H, *J*_Et1,Et2_ = 16.22 Hz, H-Et_1_), 6.99 (s, 1H, H-Ox_4_), 6.71 (d, 1H, *J*_Et2,Et1_ = 16.22 Hz, H-Et_2_), 6.62 (d, 2H, *J*_Ar2a,Ar1a_ = 8.48 Hz, H-Ar_2a_), 4.38 (s, 2H, H-CH_2_), 4,33 (s, 1H, H-NH); ^13 ^C NMR (CDCl_3_, 75 MHz) *δ*/ppm: 158.5 (s), 147.9 (s), 130.4 (s), 130.1 (d), 128.5 (d), 128.4 (s), 128.3 (d), 128.2 (2d), 127.5 (d), 125.2 (s), 122.0 (d), 113.6 (d), 112.5 (d), 112.4 (2d), 108.2 (d), 47.1 (t); HRMS (Q-TOF) for C_18_H_15_ClN_2_O: (M + H)^+^_calcd_ = 311.0946, (M + H)^+^_found_ = 311.0939.

(*E*)-*N*-(3-fluorobenzyl)-4–(2-(oxazol-5-yl)vinyl)aniline (*trans***-10**) was isolated (0.096 g (67.13%)) as yellow powder mp 92–95 °C;Rf (DCM, 100%) = 0.32; UV (EtOH) *λ*_max_/nm (*ε*/dm^3^mol^−1 ^cm^−1^): 226 (30378), 325 (Sh, 14962), 345 (17263); IR *ν*_max_/cm^−1^ (NaCl): 3419, 2926, 1606, 1520, 1487, 1448, 953, 817, 638; ^1^H NMR (CDCl_3_, 300 MHz) *δ*/ppm: 7.81 (s, 1H, H-Ox_2_), 7.32 (d, 2H, *J*_Ar1a,Ar2a_ = 8.19 Hz, H-Ar_1_), 7.18–6.99 (m, 4H, H-Ar), 7.02 (d, 1H, *J*_Et1,Et2_ = 16.38 Hz, H-Et_1_), 6.99 (s, 1H, H-Ox_4_), 6.71 (d, 1H, *J*_Et2,Et1_ = 16.38 Hz, H-Et_2_), 6.61 (d, 2H, *J*_Ar2a,Ar1a_ = 8.19 Hz, H-Ar_2_), 4.39 (s, 2H, H-CH_2_), 4,32 (s, 1H, H-NH). ^13 ^C NMR (CDCl_3_, 75 MHz) *δ*/ppm; 163.5 (s), 161.8 (s), 147.5 (s), 141.3 (s), 129.9 (d), 127.5 (2d), 125.5 (s), 122.2 (d), 122.1 (d), 113.8 (d), 113.7 (d), 113.6 (d), 113.5 (d), 112.4 (2d), 108.5 (d), 47.0 (t); HRMS(Q-TOF) for C_18_H_15_FN_2_O: (M + H)^+^_calcd_ = 295.1241, (M + H)^+^_found_ = 295.1237.

(*E*)-*N*-(2-methylbenzyl)-4–(2-(oxazol-5-yl)vinyl)aniline (*trans***-11**) was isolated (0.064 g (45.35%)) as yellow oil: Rf (DCM, 100%) = 0.30; UV (EtOH) *λ*_max_/nm (*ε*/dm^3^mol^−1 ^cm^−1^): 236 (10757), 329 (Sh, 25544), 350 (33604); IR *ν*_max_/cm^−1^ (NaCl): 3413, 3324, 2923, 1604, 1520, 1495, 1462, 953, 817, 638; ^1^H NMR (CDCl_3_, 300 MHz) *δ*/ppm: 7.81 (s, 1H, H-Ox_2_), 7.37–7.31 (m, 1H, H-Ar_3_), 7.35 (d, 2H, *J*_Ar1a,Ar2a_ = 8,81 Hz, H-Ar_1a_), 7.26–7.18 (m, 1H, H-Ar_4b_), 7.24 (d, 2H, *J*_Ar2b,Ar3b_ = *J*_Ar5b,Ar4b_ = 1,70 Hz, H-Ar_2_, H-Ar_5_), 7.04 (d, 1H, *J*_Et1,Et2_ = 16.48 Hz, H-Et_1_), 6.99 (s, 1H, H-Ox_4_), 6.72 (d, 1H, *J*_Et2,Et1_ = 16.48 Hz, H-Et_2_), 6.64 (d, 2H, *J*_Ar2a,Ar1a_ = 8.81 Hz, H-Ar_2a_), 4.33 (s, 2H, H-CH_2_), 4,10 (s, 1H, H-NH), 2,40 (s, 3H, H-CH_3_); ^13 ^C NMR (CDCl_3_, 75 MHz) *δ*/ppm 150.72 (s), 149.19 (s), 148.03 (s), 136.08 (s), 135.80 (s), 130.09 (d), 130.01 (d), 127.68 (d), 127.54 (d), 127.09 (d), 125.72 (d), 125.17 (d), 122.00 (d), 112.22 (d), 108.28 (d), 45.67 (q), 18.41 (t); HRMS(Q-TOF) for C_19_H_18_N_2_O: (M + H)^+^_calcd_ = 291.1492, (M + H)^+^_found_ = 291.1481.

(*E*)-*N*-(2-methoxybenzyl)-4–(2-(oxazol-5-yl)vinyl)aniline **(***trans***-12**) was isolated (0.050 g (33.58%)) as yellow oil: R_f_ (DCM, 100%) = 0.18; UV (EtOH) *λ*_max_/nm (*ε*/dm^3^mol^−1 ^cm^−1^): 221 (16630), 327 (Sh, 24690), 350 (32084); IR *ν*_max_/cm^−1^ (NaCl): 3413, 2930, 1605, 1521, 1490, 1463, 953, 817, 638; ^1^H NMR (CDCl_3_, 300 MHz) *δ*/ppm: 7.78 (s, 1H, H-Ox_2_), 7.32–7.23 (m, 1H, H-Ar_3b_), 7.29 (d, 2H, *J*_Ar1aAar2a_ = 8.45 Hz, H-Ar_1a_), 6.99 (d, 1H, *J*_Et1,Et2_ = 16.18 Hz, H-Et_1_), 6.96–6.88 (m, 3H, H-Ar_2b,3b,4b_), 6.95 (s, 1H, H-Ox_4_), 6.67 (d, 1H, *J*_Et2,Et1_ = 16.18 Hz, H-Et_2_), 6.63 (d, 2H, *J*_Ar2a,Ar1a_ = 8.45 Hz, H-Ar_2a_), 4.36 (s, 2H, H-CH_2_), 4.33 (s, 1H, H-NH), 3.87 (s, 3H, H-OCH_3_); ^13 ^C NMR (CDCl_3_, 75 MHz) *δ*/ppm 149.14 (s), 148.24 (s), 130.16 (d), 128.31 (d), 127.98 (d), 127.46 (d), 124.96 (d), 121.90 (d), 120.06 (d), 112.51 (d), 109.84 (d), 108.08 (d), 54.83 (q), 42.75 (t)); HRMS (Q-TOF) for C_19_H_18_N_2_O_2_: (M + H)^+^_calcd_ = 307.1441, (M + H)^+^_found_ = 307.1444.

(*E*)-*N*-(2-fluorobenzyl)-4–(2-(oxazol-5-yl)vinyl)aniline (*trans***-14**) was isolated (0.102 g (71.52%)) as yellow powder mp 119–122 °C; Rf (DCM, 100%) = 0.26; UV (EtOH) *λ*_max_/nm (*ε*/dm^3^mol^−1 ^cm^−1^): 233 (11204), 326 (Sh, 24144), 346 (28843); IR *ν*_max_/cm^−1^ (NaCl): 3398, 2926, 1605, 1520, 1487, 1455, 952, 815, 758, 638; ^1^H NMR (CDCl_3_, 300 MHz) *δ*/ppm: 7.81 (s, 1H, H-Ox_2_), 7.40–7.25 (m, 1H, H-Ar_3b_), 7.34 (d, 2H, *J*_Ar1a,Ar2a_ = 8.15 Hz, H-Ar_1a_), 7.14–6.97 (m, 3H, H-Ar_2b,3b,4b_), 7.02 (d, 1H, *J*_Et1,Et2_ = 16.30 Hz, H-Et_1_), 6.99 (s, 1H, H-Ox_4_), 6.70 (d, 1H, *J*_Et2,Et1_ = 16.30 Hz, H-Et_2_), 6.64 (d, 2H, *J*_Ar2a,Ar1a_ = 8.15 Hz, H-Ar_2a_), 4.45 (s, 2H, H-CH_2_), 4.29 (s, 1H, H-NH); ^13 ^C NMR (CDCl_3_, 75 MHz) *δ*/ppm 149.37 (s), 147.58 (s), 129.98 (d), 131.84 (d), 128.40 (d), 127.51 (d), 125.46 (d), 123.74 (d), 122.07 (d), 114.88 (d), 112.47 (d), 108.46 (d), 41.17 (t); HRMS(Q-TOF) for C_18_H_15_FN_2_O: (M + H)^+^_calcd_ = 295.1241, (M + H)^+^_found_ = 295.1236.

(*E*)-*N*-(furan-2-ylmethyl)-4–(2-(oxazol-5-yl)vinyl)aniline (*trans***-17**) was isolated (0.050 g (38.63%)) as yellow powder mp 97–102 °C; Rf (DCM, 100%) = 0.22; UV (EtOH) *λ*_max_/nm (*ε*/dm^3^mol^−1 ^cm^−1^): 215 (Sh, 16540), 241 (Sh, 10289), 325 (Sh, 22270), 346 (27578); IR *ν*_max_/cm^−1^ (NaCl): 3407, 2926, 1609, 1519, 1492, 964, 951, 820, 738, 637; ^1^H NMR (CDCl_3_, 300 MHz) *δ*/ppm: 7.79 (s, 1H, H-Ox_2_), 7.37 (d, 1H, *J*_Fur5,Fur4_ = 1.80 Hz, H-Fur_5_), 7.32 (d, 2H, *J*_Ar1_,_Ar2_ = 8.48 Hz, H-Ar_1_), 7.00 (d, 1H, *J*_Et1,Et2_ = 16.60 Hz, H-Et_1_), 6.97 (s, 1H, H-Ox_4_), 6.69 (d, 1H, *J*_Et2,Et1_ = 16.60 Hz, H-Et_2_), 6.65 (d, 2H, *J*_Ar2,Ar1_ = 8.48 Hz, H-Ar_2_), 6.32 (dd, 1H, *J*_Fur4,Fur5_ = 1.81 Hz, *J*_Fur4,Fur3_ = 3.16 Hz, H-Fur_4_), 6,24 (d, 1H, *J*_Fur3,Fur4_ = 3.16 Hz, H-Fur_3_), 4.35 (s, 2H, H-CH_2_), 4.21 (s, 1H, H-NH); ^13 ^C NMR (CDCl_3_, 75 MHz) *δ*/ppm 151.52 (s), 150.64 (s), 149.23 (s), 147.37 (s), 141.55 (d), 129.97 (d), 127.46 (d), 125.66 (d), 122.10 (d), 112.63 (d), 109.87 (d), 108.55 (d), 106.65 (d), 40.69 (t).

(*E*)-4–(2-(oxazol-5-yl)vinyl)-*N*-(thiophen-2-ylmethyl)aniline (*trans***-18**) was isolated (11.1 mg (17.10%)) as yellow oil; Rf (DCM) = 0.42; UV (EtOH) *λ*_max_/nm: 274 (12817), 291 (13098); IR *ν*_max_/cm^−1^ (NaCl): 3359, 2811, 1708, 1665; ^1^H NMR (CDCl_3_, 300 MHz) *δ*/ppm: 7.62 (s, 1H, H-Ox2), 7.20–7.09 (m, 4H), 7.07 (d, 1H, *J* = 5.1 Hz), 6.98 (d, 1H, *J* = 3.2 Hz), 6.79 (dd, 1H, *J* = 5.1, 3.2 Hz), 6.63 (s, 1H), 6.55 (d, 1H, *J* = 12.3 Hz), 6.23 (d, 1H, *J* = 12.3 Hz), 4.62 (s, 2H), 4.31 (s, 1H); MS *m/z* (%, fragment) (EI): 282 (M^+^, 100); HRMS (*m/z*): [M + H]^+^ calcd for 283.0827; found for 283.0848.

#### 4.1.4. Photochemistry of the new (E)-N-benzyl-4(2-(oxazol-5-yl)vinyl)anilines

A quartz vessel was charged with (*E*)-*N*-aryl-4(2-(oxazol-5-yl)vinyl)anilines (*trans***-2**–**8,** *trans***-10–12** and *trans***-17**) in 50 ml of toluene (0.003 mmol/mL) with the addition of a small amount of iodine and irradiated at 350 nm in the Rayonet reactor for 2 h, 3 h, 4 h and 8 h. The conversion was followed by thin-layer chromatography. After irradiation, the solvent was removed in vacuum and the residue chromatographed on a silica gel column using dichloromethane as eluent. In the first fractions, the photoelectrocyclized products **19**–**21** were isolated, and in the last fractions the unreacted started amines and photolyzed products *cis*-**18**, *cis*-**22** and **23**.

*N*-benzylnaphtho[1,2-*d*]oxazol-8-amine (**19**) was isolated (25.0 mg (37.40%)) as yellow oil; R_f_ (DCM) = 0.53; UV (EtOH) *λ_max_*/nm: 247 (15132), 299 (5098), 344 (4899); IR *ν*_max_/cm^−1^ (NaCl): 3396, 2922, 2849, 1735, 1635, 1535; ^1^H NMR (CDCl_3_, 300 MHz) *δ*/ppm: 8.15 (s, 1H, H-Ox2), 7.75 (d, 1H, *J* = 8.8 Hz), 7.66 (d, 1H, *J* = 8.8 Hz), 7.52 (d, 1H, *J* = 2.4 Hz), 7.43 (t, 3H, *J* = 7.2 Hz), 7.35 (t, 2H, *J* = 7.3 Hz), 7.31–7.28 (m, 1H), 6.94 (dd, 1H, *J* = 8.7; 2.5 Hz), 4.52 (s, 2H); ^13 ^C NMR (CDCl_3_, 75 MHz) *δ*/ppm: 149.7 (s), 146.2 (s), 138.2 (s), 129.3 (2d), 128.2 (d), 128.7 (s), 127.7 (s), 127.3 (2d), 126.9 (d), 126.4 (d), 125.9 (d), 124.2 (s), 116.1 (d); 105.9 (d), 98.8 (d), 47.8 (t); MS *m/z* (%, fragment) (EI): 274 (M^+^, 100); HRMS (*m/z*): [M + H]^+^ calcd for 275.1106; found for 275.1119.

*N*-(4-fluorobenzyl)naphtho[1,2-*d*]oxazol-8-amine (**20**) was isolated (18.6 mg (26.4%)) as yellow oil; R_f_ (DCM) = 0.55; UV (EtOH) *λ_max_*/nm: 247 (5711), 310 (4677), 339 (2013); IR *ν*_max_/cm^−1^ (NaCl): 3414, 2922, 1637, 1601, 1533, 1508, 1255, 1222; ^1^H NMR (CDCl_3_, 300 MHz) *δ*/ppm: 8.11 (s, 1H, H-Ox2), 7.75 (d, 1H, *J* = 8.5 Hz), 7.66 (d, 1H, *J* = 8.8 Hz), 7.49 (d, 1H, *J* = 2.5 Hz), 7.43 (d, 2H, *J* = 8.3 Hz), 7.42–7.39 (m, 2H), 7.04 (d, 2H, *J* = 8.1 Hz), 6.94 (dd, 1H, *J* = 8.8; 2.5 Hz), 4.50 (s, 2H), 4.39 (s, 1H, NH); ^13 ^C NMR (CDCl_3_, 75 MHz) *δ*/ppm: 163.7 (s), 150.8 (s), 148.2 (s), 146.9 (s), 134.5 (s), 129.8 (d), 129.3 (d), 129.2 (d), 126.4 (d), 124.7 (s), 116.5 (d), 115.7 (d), 115.4 (d), 106.6 (d), 99.3 (d); 47.5 (t); MS *m/z* (%, fragment) (EI): 292 (M^+^, 100); HRMS (*m/z*): [M + H]^+^ calcd for 293.1012; found for 293.1004.

*N*-(thiophen-2-ylmethyl)naphtho[1,2-*d*]oxazol-8-amine (**21**) was isolated (8.5 mg (13.0%)) as yellow oil; R_f_ (DCM) = 0.67; UV (EtOH) *λ_max_*/nm: 231 (19138); IR *ν*_max_/cm^−1^ (NaCl): 3393, 2917, 2310, 1731, 1539, 1460; ^1^H NMR (CDCl_3_, 300 MHz) *δ*/ppm: 8.16 (s, 1H, H-Ox2), 7.76 (d, 1H, *J* = 8.8 Hz), 7.67 (d, 1H, *J* = 8.8 Hz), 7.56 (d, 1H, *J* = 2.3 Hz), 7.44 (d, 1H, *J* = 8.9 Hz), 7.24 (d, 1H, *J* = 5.0 Hz), 7.10 (d, 1H, *J* = 3.5 Hz), 6.99 (dd, 1H, *J* = 5.0, 3.5 Hz), 6.95 (dd, 1H, *J* = 8.8; 2.5 Hz), 4.71 (s, 2H), 4.40 (s, 1H); ^13 ^C NMR (CDCl_3_, 75 MHz) *δ*/ppm: 153.7 (s), 149.3 (s), 136.9 (s), 129.9 (d), 128.7 (d), 128.1 (d), 127.5 (s), 127.0 (d), 126.6 (d), 125.3 (d), 124.4 (s), 116.4 (d), 106.2 (d), 99.5 (d), 50.1 (t); MS *m/z* (%, fragment) (EI): 280 (M^+^, 100); HRMS (*m/z*): [M + H]^+^ calcd for 281.0670; found for 281.0691.

(*Z*)-4–(2-(oxazol-5-yl)vinyl)-*N*-(thiophen-2-ylmethyl)aniline (*cis*-**18**) was isolated (11.1 mg (17.10%)) as yellow oil; R_f_ (DCM) = 0.42; UV (EtOH) *λ*_max_/nm: 274 (12817), 291 (13098); IR *ν*_max_/cm^−1^ (NaCl): 3359, 2811, 1708, 1665; ^1^H NMR (CDCl_3_, 300 MHz) *δ*/ppm: 7.62 (s, 1H, H-Ox2), 7.20–7.09 (m, 4H), 7.07 (d, 1H, *J* = 5.1 Hz), 6.98 (d, 1H, *J* = 3.2 Hz), 6.79 (dd, 1H, *J* = 5.1, 3.2 Hz), 6.63 (s, 1H), 6.55 (d, 1H, *J* = 12.3 Hz), 6.23 (d, 1H, *J* = 12.3 Hz), 4.62 (s, 2H), 4.31 (s, 1H); MS m/z (%, fragment) (EI): 282 (M+, 100); HRMS (m/z): [M + H]+ calcd for 283.0827; found for 283.0848.

(*Z*)-4–(2-(oxazol-5-yl)vinyl)aniline (*cis-***22**) was isolated (4.5 mg (7.2%)) as yellow oil; R_f_ (DCM) = 0.43; UV (EtOH) *λ*_max_/nm: 259 (13474), 265 (12817), 272 (13409); IR *ν*_max_/cm^−1^ (NaCl): 3412, 2917, 2844, 1737, 1632, 1462; UV (EtOH) *λ*_max_/nm: 259 (13474), 265 (12817), 272 (13409); ^1^H NMR (CDCl_3_, 300 MHz) *δ*/ppm: 7.73 (s, 1H, H-Ox2), 7.40–7.33 (m, 4H), 7.32 (s, 2H), 6.91 (s, 1H), 6.67 (d, 1H, *J* = 12.5 Hz), 6.39 (d, 1H, *J* = 12.5 Hz); MS *m/z* (%, fragment) (EI): 186 (M^+^, 100); HRMS (*m/z*): [M + H]^+^ calcd for 186.0793; found for 186.0782.

Naphtho[1,2-*d*]oxazol-8-amine (**23**) was isolated (3.9 mg (6.4%)) as yellow oil; R_f_ (PE/DCM = 1:1) = 0.23; UV (EtOH) *λ_max_*/nm: 225 (18143), 353 (4881), 400 (4012); IR *ν*_max_/cm^−1^ (NaCl): 3414, 2922, 1637, 1601, 1533, 1508, 1255, 1222; ^1^H NMR (CDCl_3_, 300 MHz) *δ*/ppm: 7.37 (s, 1H, H-Ox2), 7.34 (d, 1H, *J* = 8.9 Hz), 6.93 (s, 1H), 6.86 (d, 1H, *J* = 10.0 Hz), 6.72 (d, 1H, *J* = 8.9 Hz), 6.65 (d, 1H, *J* = 10.0 Hz), 3.01 (s, 2H, NH_2_); MS *m/z* (%, fragment) (EI): 184 (M^+^, 100); HRMS (*m/z*): [M + H]^+^ calcd for 185.0637; found for 185.0644.

### 4.2. Reversible inhibition of cholinesterases by novel oxazole benzylamine compounds

Inhibition potency of novel compounds was evaluated for recombinant human AChE (prepared as described earlier^25^ and kindly donated by Prof Palmer Taylor, Skaggs School of Pharmacy and Pharmaceutical Sciences, University of California at San Diego, La Jolla, USA) and BChE isolated from human plasma (kindly donated by late Dr Douglas Cerasoli and Dr David Lenz, USAMRICD, Edgewood, MD). The inhibition mixture contained a 0.1 M phosphate buffer, pH 7.4, enzyme, tested compound, and reagent, 5,5'-dithiobis(2-nitrobenzoic acid) (DTNB, 0.3 mM; Sigma Chemical Co., St. Louis, MO, USA). Enzyme activity was measured upon addition of substrate, acetylthiocholine (ATCh, 0.2 or 0.1 mM; Sigma Chemical Co., St. Louis, MO, USA) by the Ellman method^26^ at 25 °C and 412 nm, on a Tecan Infinite M200PRO plate reader (Tecan Austria, GmbH, Salzburg, Austria). Due to the low solubility, a stock solution of the tested compounds was prepared in DMSO or methanol (Kemika, Zagreb, Croatia), and a corresponding solvent was in controls as well. The IC_50_ values were determined from at least three experiments by a nonlinear fit of the compound concentration logarithm values vs. % of enzyme activity using Prism6 software (GraphPad Prism 6 Software, San Diego, USA).

## Supporting information

^1^H and ^13 ^C NMR spectra of all the newly synthesised and isolated compounds, along with the 2 D NMR spectra of some compounds as well as AChE inhibition by *trans-*amino-5-arylethenyl-oxazole derivatives.

## Supplementary Material

Supplemental Material
