# Supplementary material for: Design, synthesis and cholinesterase inhibitory properties of new oxazole benzylamine derivatives
Source: J Enzyme Inhib Med Chem. 2020 Jan 3;35(1):460–7. doi: 10.1080/14756366.2019.1707197 (PMC6968547; doi:10.1080/14756366.2019.1707197)

Supplemental Material

Design, synthesis and cholinesterase inhibitory properties of new oxazole benzylamine derivatives

Ivana Šagud[1,§], Nikolina Maček Hrvat[2,§], Ana Grgičević[1], Tena Čadež[2], Josipa Hodak[1], Milena Dragojević[1], Kornelija Lasić[3], Zrinka Kovarik[2*] and Irena Škorić[1*]

[1]Department of Organic Chemistry, Faculty of Chemical Engineering and Technology, University of Zagreb, Marulićev trg 19, 10000 Zagreb, Croatia
[2]Institute for Medical Research and Occupational Health, Ksaverska cesta 2, 10000 Zagreb, Croatia
[3]Pliva Tapi R&D, TEVA, Prilaz Baruna Filipovića 25, 10000 Zagreb, Croatia

[§]These authors contributed equally.
*Corresponding authors:
Dr. Zrinka Kovarik, zkovarik@imi.hr
Prof. Irena Škorić, iskoric@fkit.hr

CONTENT

Figure S1. ^{1}H and ^{13}C NMR spectra of (*E*)-*N*-benzyl-4-(2-(oxazol-5-yl)vinyl)aniline **(*trans*-2)**

Figure S2. ^{1}H and ^{13}C NMR spectra of (*E*)-*N*-(4-methylbenzyl)-4-(2-(oxazol-5-yl)vinyl)aniline **(*trans*-3)**

Figure S3. ^{1}H and ^{13}C NMR spectra of (*E*)-*N*-(4-methoxybenzyl)-4-(2-(oxazol-5-yl)vinyl)aniline **(*trans*-4)**

Figure S4. ^{1}H and ^{13}C NMR spectra of (*E*)-*N*-(4-chlorobenzyl)-4-(2-(oxazol-5-yl)vinyl)aniline **(*trans*-5)**

Figure S5. ^{1}H and ^{13}C NMR spectra of (*E*)-*N*-(4-fluorobenzyl)-4-(2-(oxazol-5-yl)vinyl)aniline **(*trans*-6)**

Figure S6. ^{1}H and ^{13}C NMR spectra of (*E*)-*N*-(3-methylbenzyl)-4-(2-(oxazol-5-yl)vinyl)aniline **(*trans*-7)**

Figure S7. COSY spectra of (*E*)-*N*-(3-methylbenzyl)-4-(2-(oxazol-5-y)vinyl)aniline (*trans*-**7**)

Figure S8. HETCOR spectra of (*E*)-*N*-(3-methylbenzyl)-4-(2-(oxazol-5-il)vinil)aniline (*trans*-**7**)

Figure S9. NOESY spectra of (*E*)-*N*-(3-methylbenzyl)-4-(2-(oxazol-5-yl)vinyl)aniline (*trans*-**7**)

Figure S10. ^{1}H and ^{13}C NMR spectra of (*E*)-*N*-(3-methoxybenzyl)-4-(2-(oxazol-5-yl)vinyl)aniline **(*trans*-8)**

Figure S11. ^{1}H and ^{13}C NMR spectra of (*E*)-*N*-(3-chlorobenzyl)-4-(2-(oxazol-5-yl)vinyl)aniline **(*trans*-9)**

Figure S12. ^{1}H and ^{13}C NMR spectra of (*E*)-*N*-(3-fluorobenzyl)-4-(2-(oxazol-5-yl)vinyl)aniline **(*trans*-10)**

Figure S13. ^{1}H and ^{13}C NMR spectra of (*E*)-*N*-(2-methylbenzyl)-4-(2-(oxazol-5-yl)vinyl)aniline **(*trans*-11)**

Figure S14. ^{1}H and ^{13}C NMR spectra of (*E*)-*N*-(2-methoxybenzyl)-4-(2-(oxazol-5-yl)vinyl)aniline **(*trans*-12)**

Figure S15. ^{1}H and ^{13}C NMR spectra of (*E*)-*N*-(2-fluorobenzyl)-4-(2-(oxazol-5-yl)vinyl)aniline **(*trans*-14)**

Figure S16. ^{1}H and ^{13}C NMR spectra of (*E*)-*N*-(furan-2-ylmethyl)-4-(2-(oxazol-5-yl)vinyl)aniline **(*trans*-17)**

Figure S17. ^{1}H and ^{13}C NMR spectra of *N*-benzylnaphtho[1,2-*d*]oxazol-8-amine **(19)**

Figure S18. ^{1}H and ^{13}C NMR spectra of *N*-(4-fluorobenzyl)naphtho[1,2-*d*]oxazol-8-amine **(20)**

Figure S19. ^{1}H NMR spectrum of *N*-(thiophen-2-ylmethyl)naphtho[1,2-*d*]oxazol-8-amine **(21)**

Figure S20. Acetylcholinesterase inhibition by *trans*-amino-5-arylethenyl-oxazole derivatives.

Figure S1. ^{1}H and ^{13}C NMR spectra of (*E*)-*N*-benzyl-4-(2-(oxazol-5-yl)vinyl)aniline (***trans*-2**)

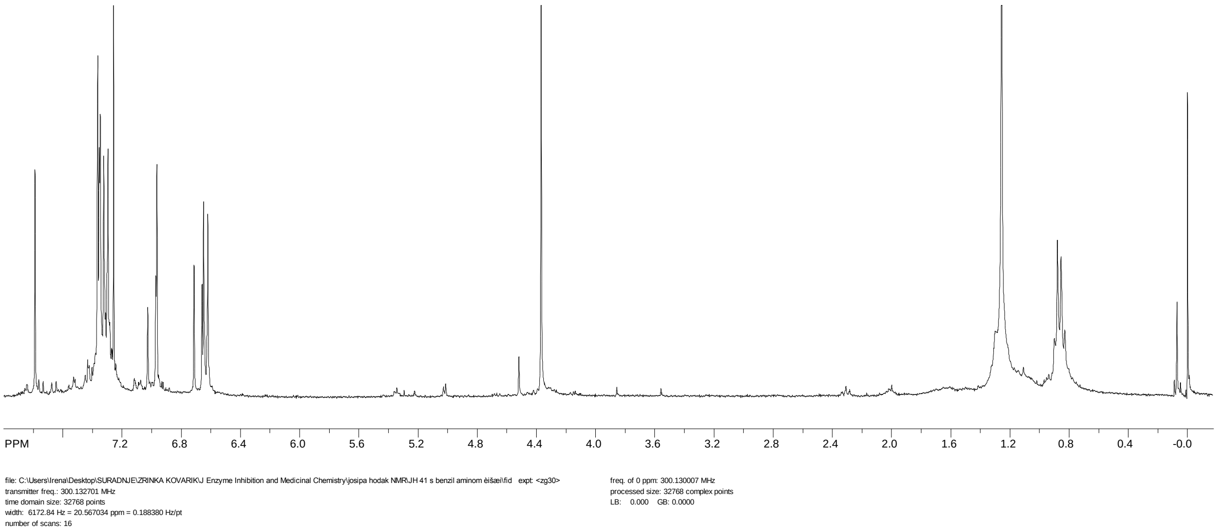

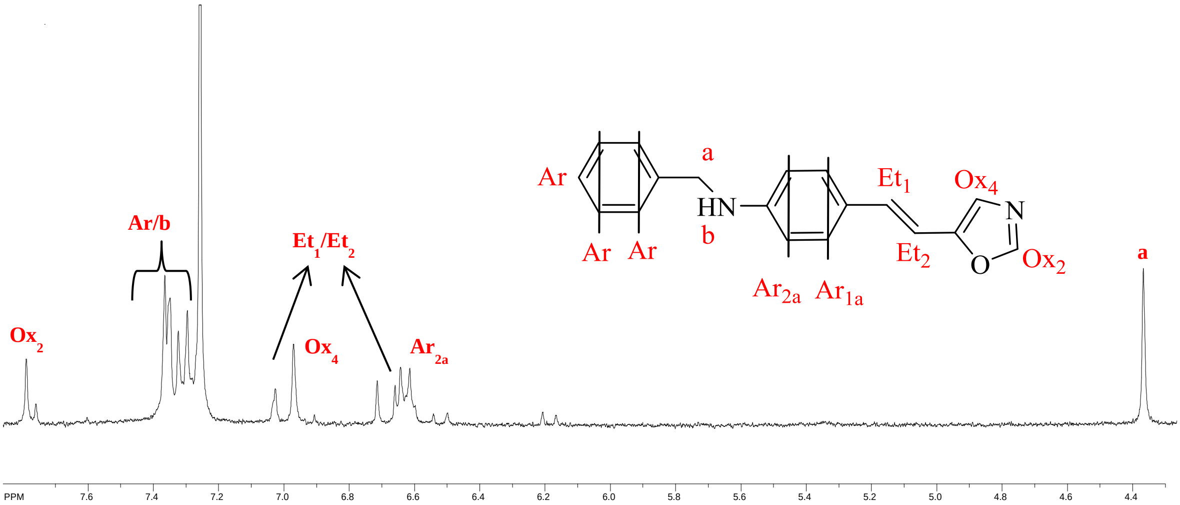

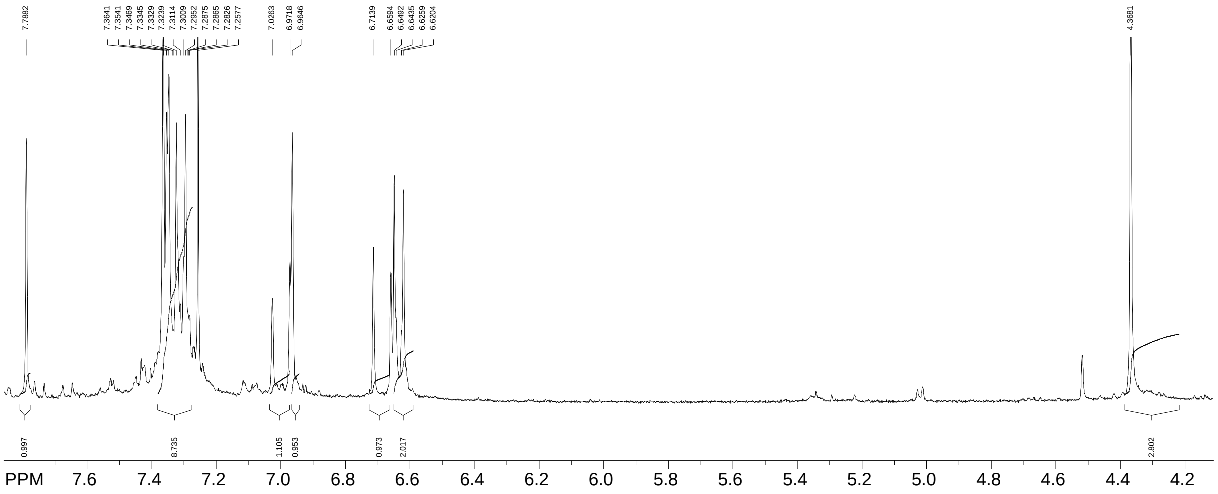
7.7882
7.3641 7.3541 7.3469 7.3345 7.3329 7.3239 7.3114 7.3009 7.2962 7.2875 7.2865 7.2826 7.2577
7.0263 6.9718 6.9646
6.7139 6.6594 6.6492 6.6435 6.6259 6.6204
4.3681
0.997 8.735 1.105 0.953 0.973 2.017 2.802
PPM 7.6 7.4 7.2 7.0 6.8 6.6 6.4 6.2 6.0 5.8 5.6 5.4 5.2 5.0 4.8 4.6 4.4 4.2

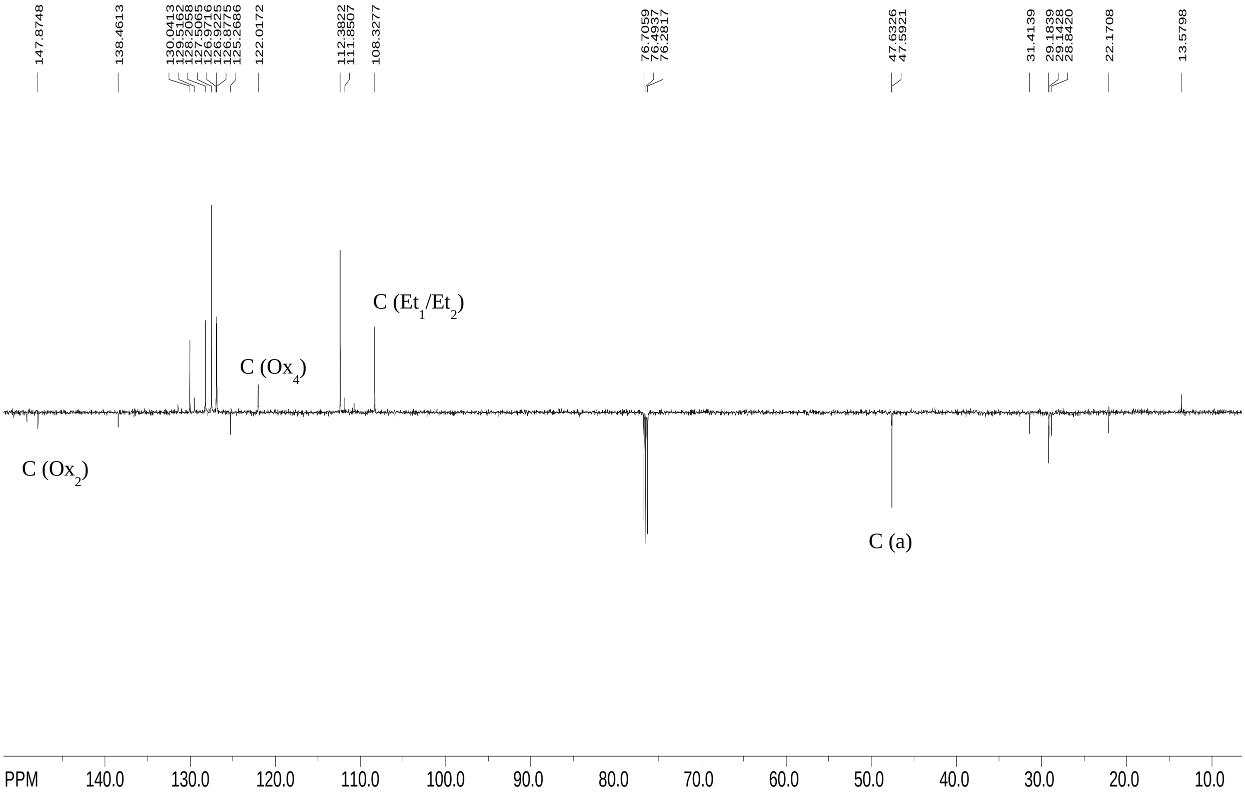
147.8748
138.4613
130.0413 129.5162 128.2058 127.5065 126.9716 126.9225 126.8275 125.2686
122.0172
112.3822 111.8507
108.3277
76.7059 76.4937 76.2817
47.6326 47.5921
31.4139
29.1839 29.1428 28.8420
22.1708
13.5798
C (Et1/Et2)
C (Ox4)
C (Ox2)
C (a)
PPM 140.0 130.0 120.0 110.0 100.0 90.0 80.0 70.0 60.0 50.0 40.0 30.0 20.0 10.0

Figure S2. ^{1}H and ^{13}C NMR spectra of (*E*)-*N*-(4-methylbenzyl)-4-(2-(oxazol-5-yl)vinyl)aniline (*trans*-**3**)

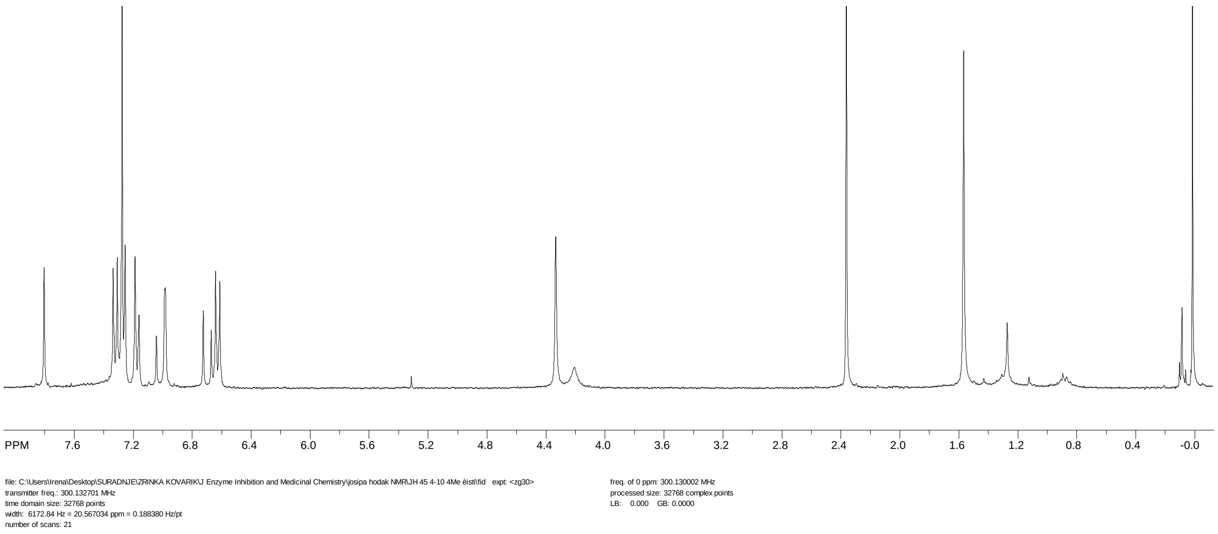

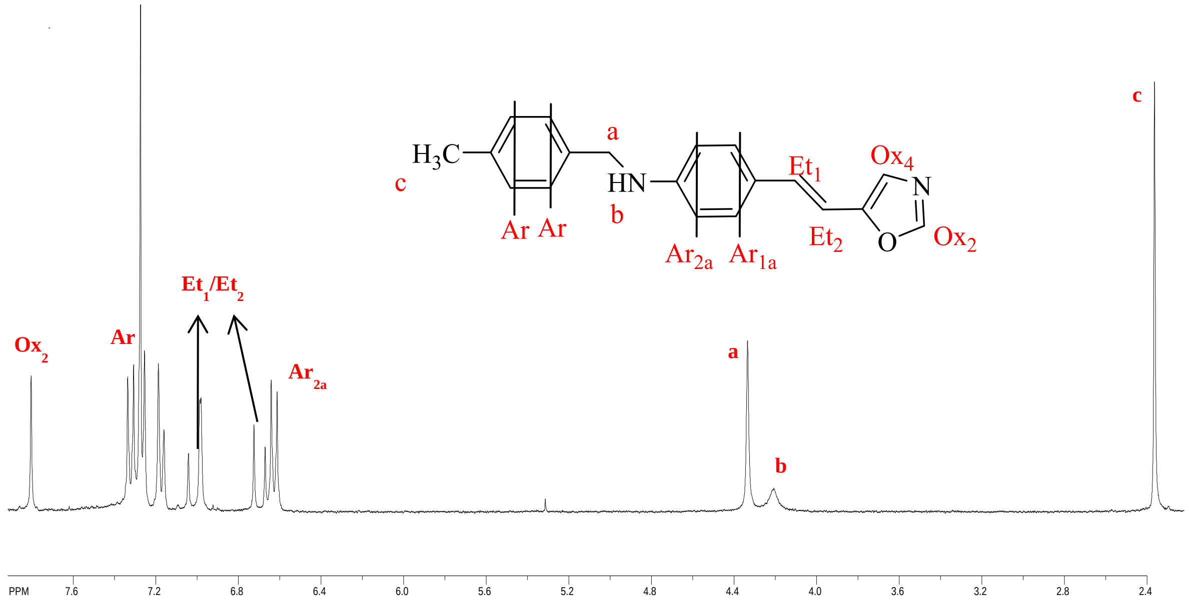

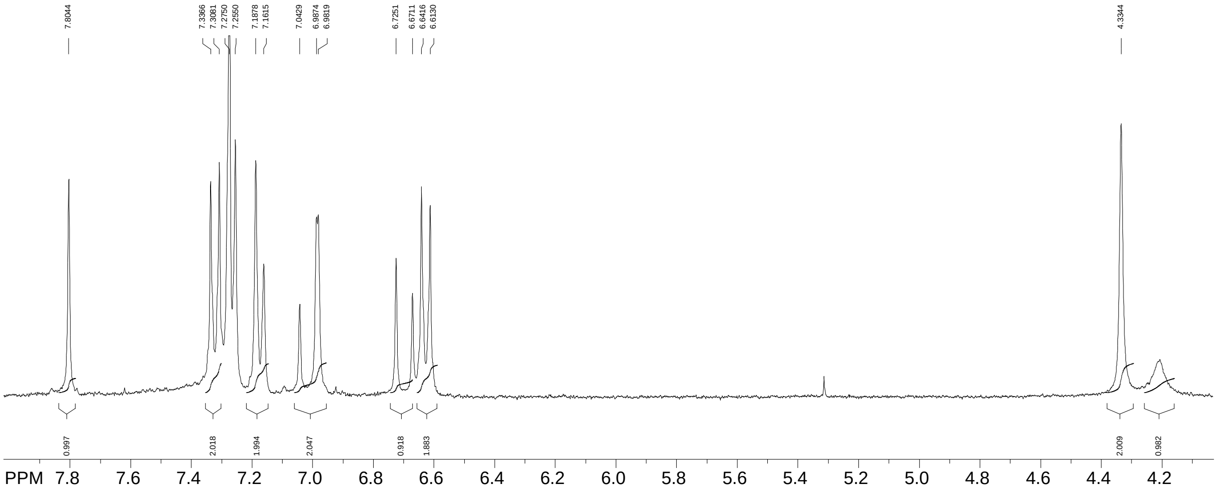
PPM 7.8 7.6 7.4 7.2 7.0 6.8 6.6 6.4 6.2 6.0 5.8 5.6 5.4 5.2 5.0 4.8 4.6 4.4 4.2

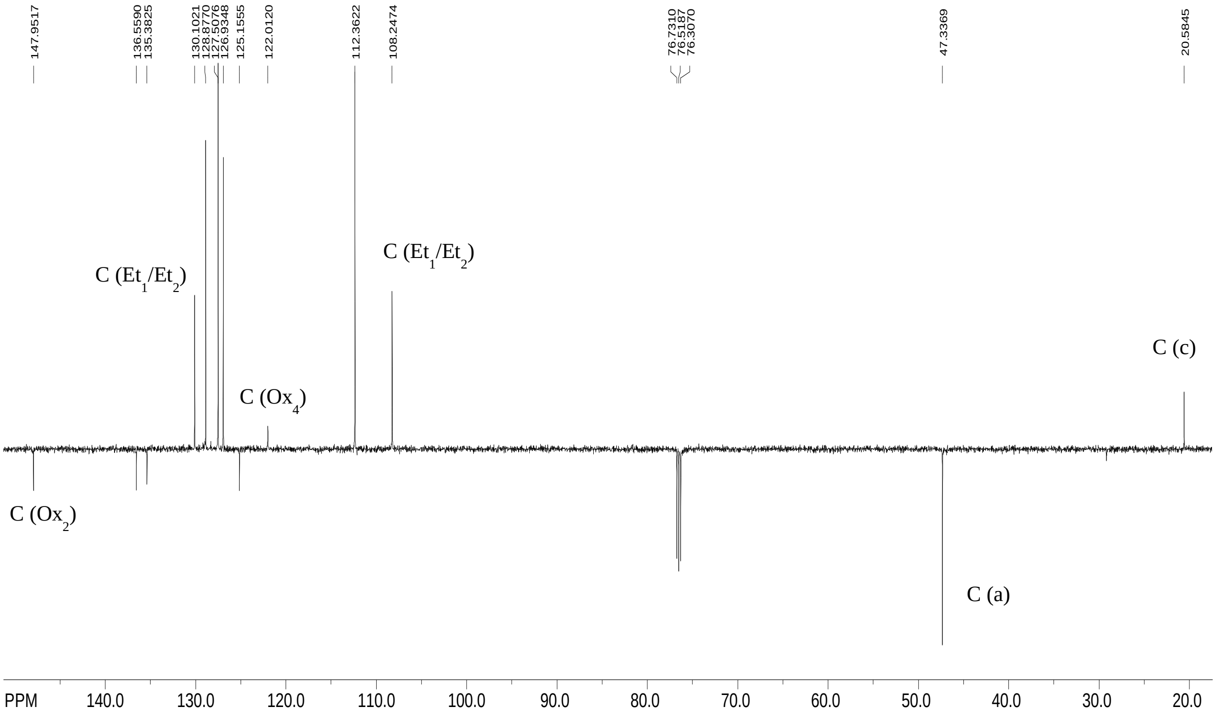
147.9517
136.5590
135.3825
130.1021
128.8770
127.5076
126.9348
125.1555
122.0120
112.3622
108.2474
76.7310
76.5187
76.3070
47.3369
20.5845
C (Et1/Et2)
C (Et1/Et2)
C (c)
C (Ox4)
C (Ox2)
C (a)
PPM 140.0 130.0 120.0 110.0 100.0 90.0 80.0 70.0 60.0 50.0 40.0 30.0 20.0

Figure S3. ^{1}H and ^{13}C NMR spectra of (*E*)-*N*-(4-methoxybenzyl)-4-(2-(oxazol-5-yl)vinyl)aniline (*trans*-**4**)

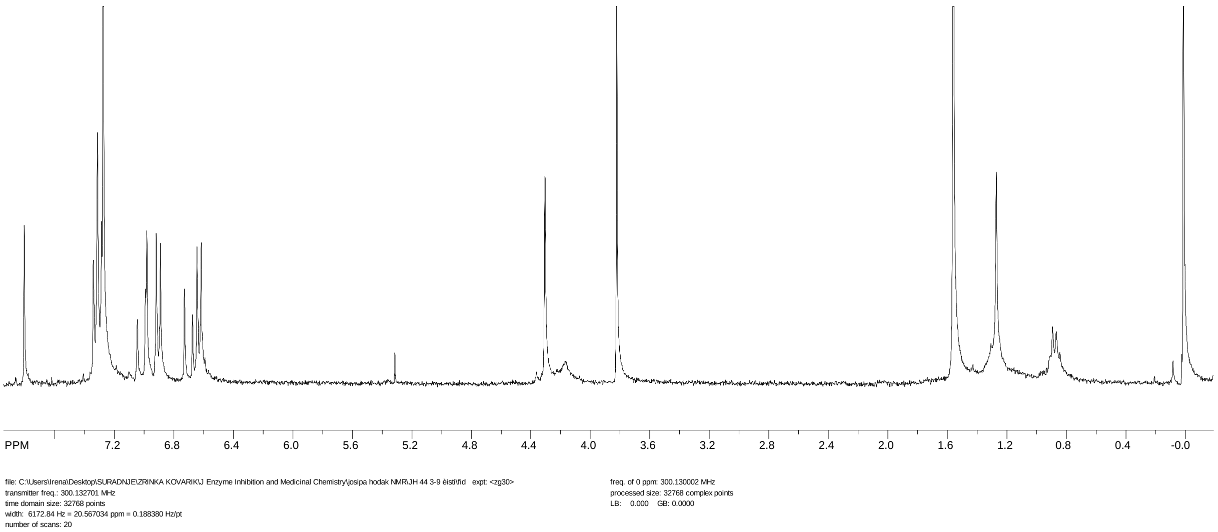

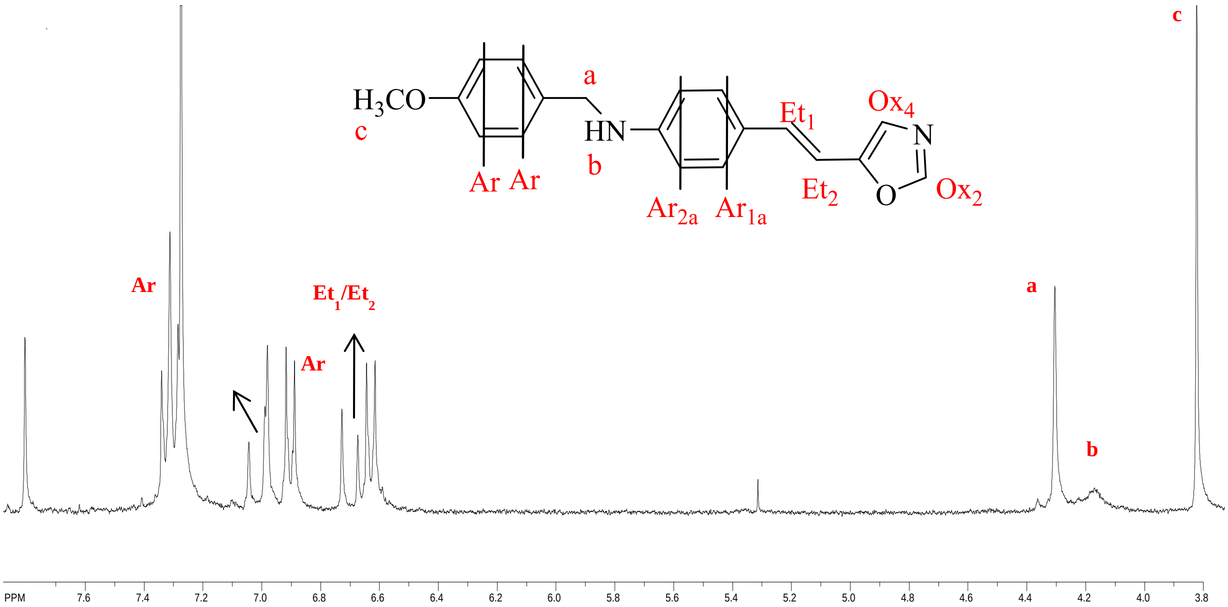

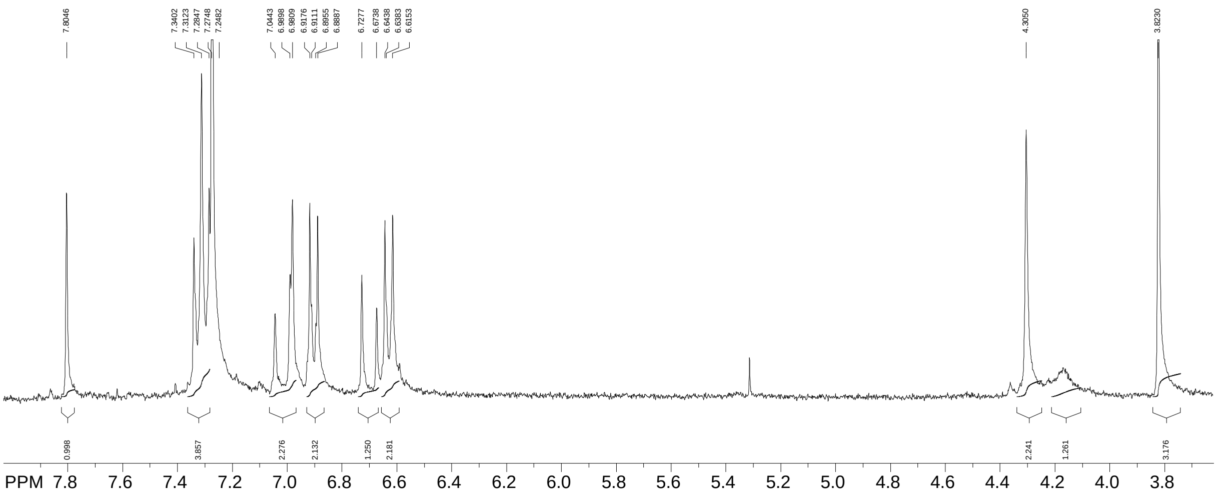
PPM 7.8 7.6 7.4 7.2 7.0 6.8 6.6 6.4 6.2 6.0 5.8 5.6 5.4 5.2 5.0 4.8 4.6 4.4 4.2 4.0 3.8

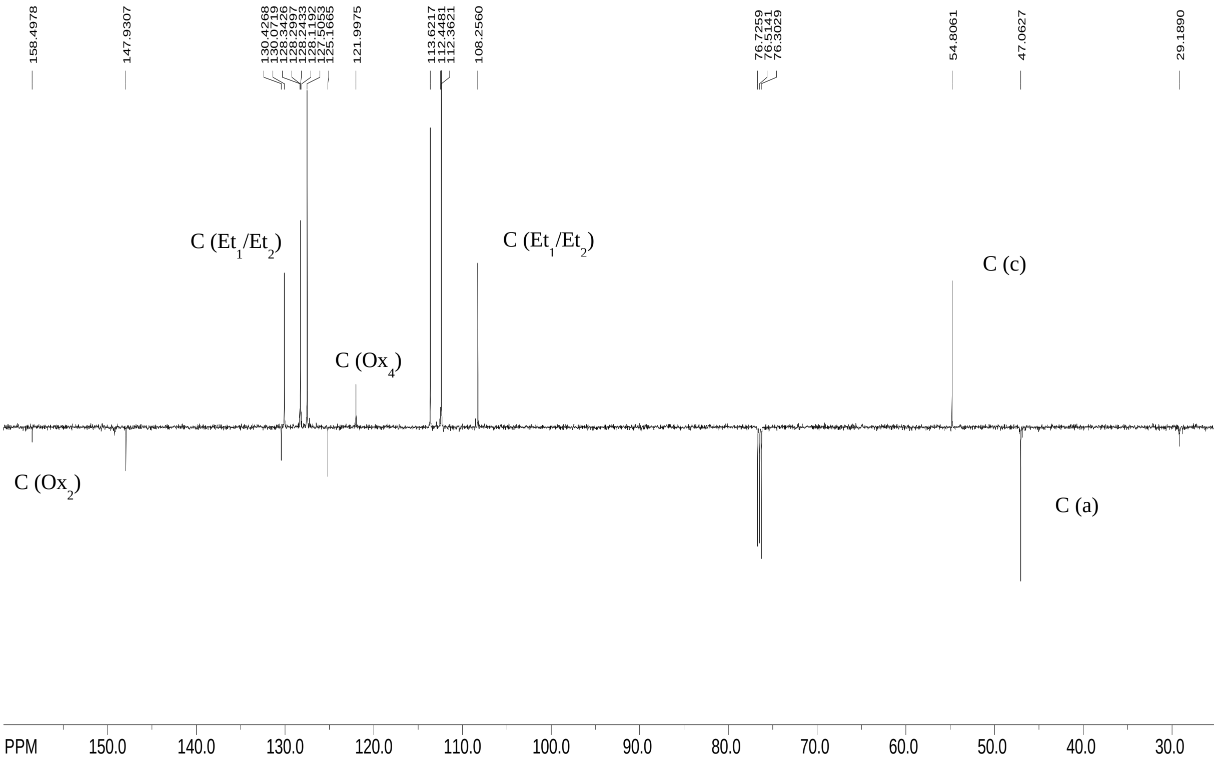
158.4978
147.9307
130.4268
130.0719
128.3426
128.2997
128.2433
128.1192
127.5053
125.1665
121.9975
113.6217
112.4481
112.3621
108.2560
76.7259
76.5141
76.3029
54.8061
47.0627
29.1890
C (Et1/Et2)
C (Et1/Et2)
C (c)
C (Ox4)
C (Ox2)
C (a)
PPM 150.0 140.0 130.0 120.0 110.0 100.0 90.0 80.0 70.0 60.0 50.0 40.0 30.0

Figure S4. ^{1}H and ^{13}C NMR spectra of (*E*)-*N*-(<u>4-chlorobenzyl</u>)-4-(2-(oxazol-5-yl)vinyl)aniline (*trans*-**5**)

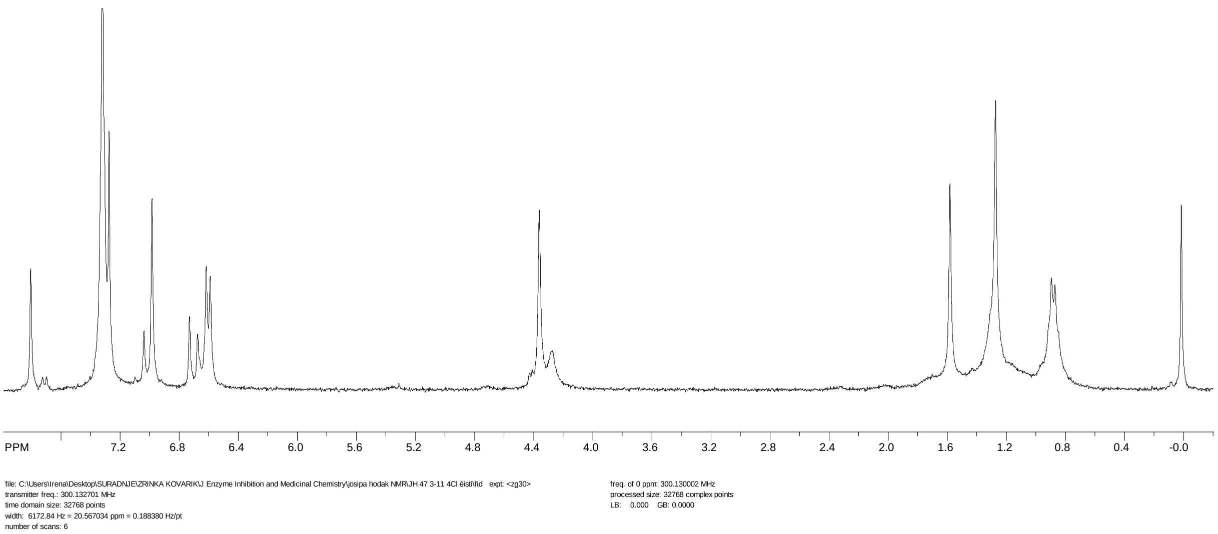

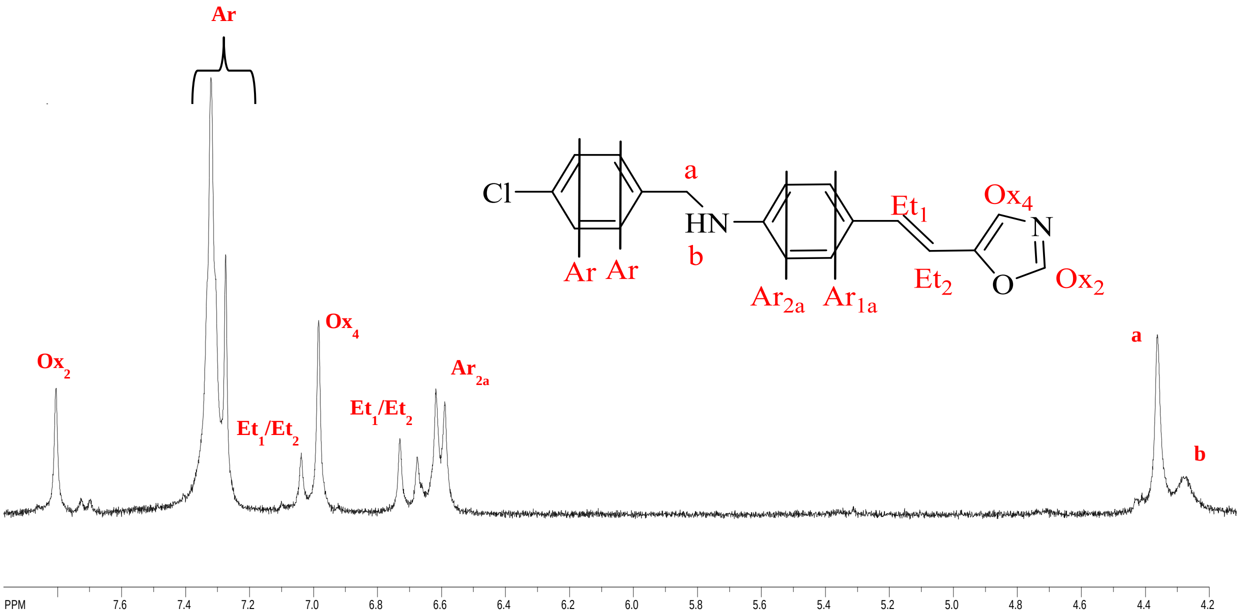

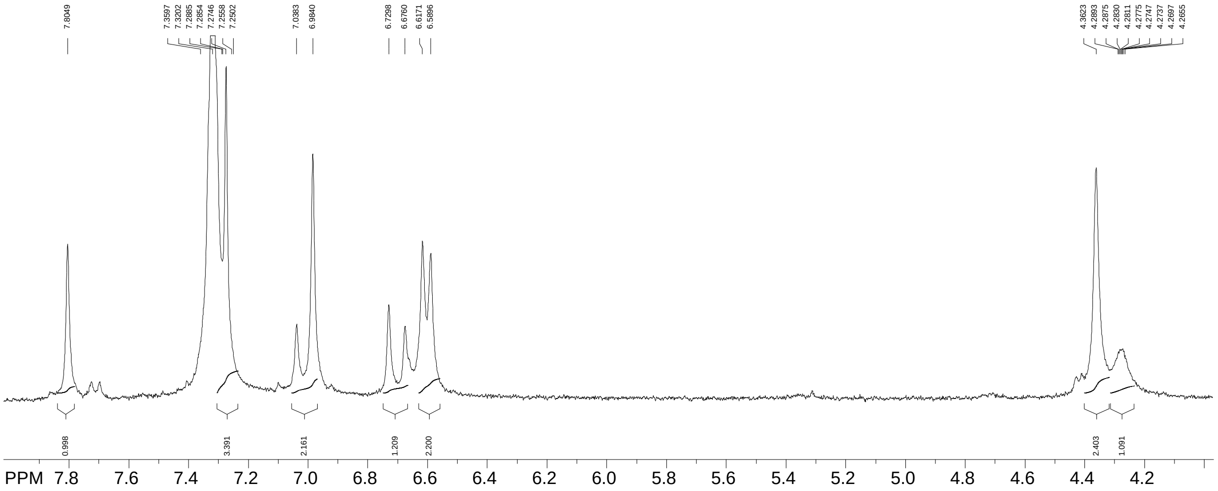
7.8049
7.3597 7.3202 7.2885 7.2854 7.2746 7.2558 7.2502
7.0383 6.9840
6.7298 6.6760 6.6171 6.5896
4.3623 4.2893 4.2875 4.2830 4.2811 4.2775 4.2747 4.2737 4.2697 4.2655
0.998 3.391 2.161 1.209 2.200 2.403 1.091
PPM 7.8 7.6 7.4 7.2 7.0 6.8 6.6 6.4 6.2 6.0 5.8 5.6 5.4 5.2 5.0 4.8 4.6 4.4 4.2

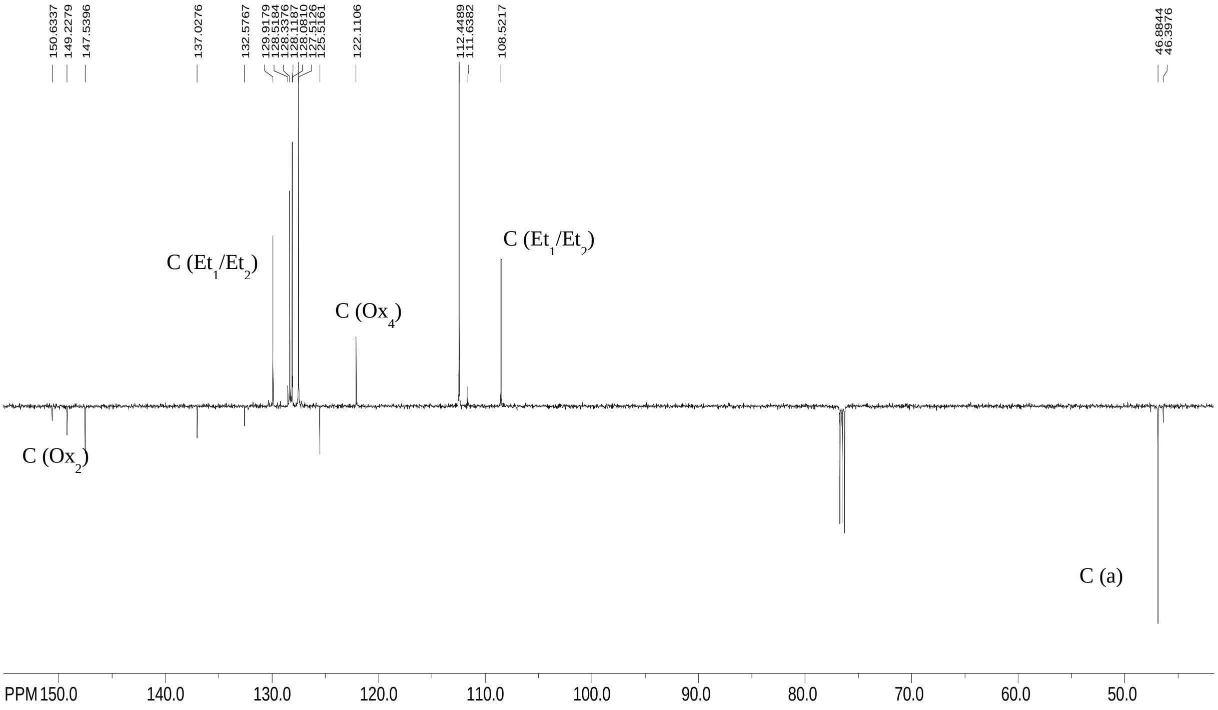
150.6337 149.2279 147.5396
137.0276
132.5767
129.9179 128.5184 128.3376 128.1187 128.0810 127.5126 125.5161
122.1106
112.4489 111.6382
108.5217
46.8844 46.3976
C (Et1/Et2)
C (Ox4)
C (Et1/Et2)
C (Ox2)
C (a)
PPM 150.0 140.0 130.0 120.0 110.0 100.0 90.0 80.0 70.0 60.0 50.0

Figure S5. ^{1}H and ^{13}C NMR spectra of (*E*)-*N*-(4-fluorobenzyl)-4-(2-(oxazol-5-yl)vinyl)aniline (*trans*-**6)**

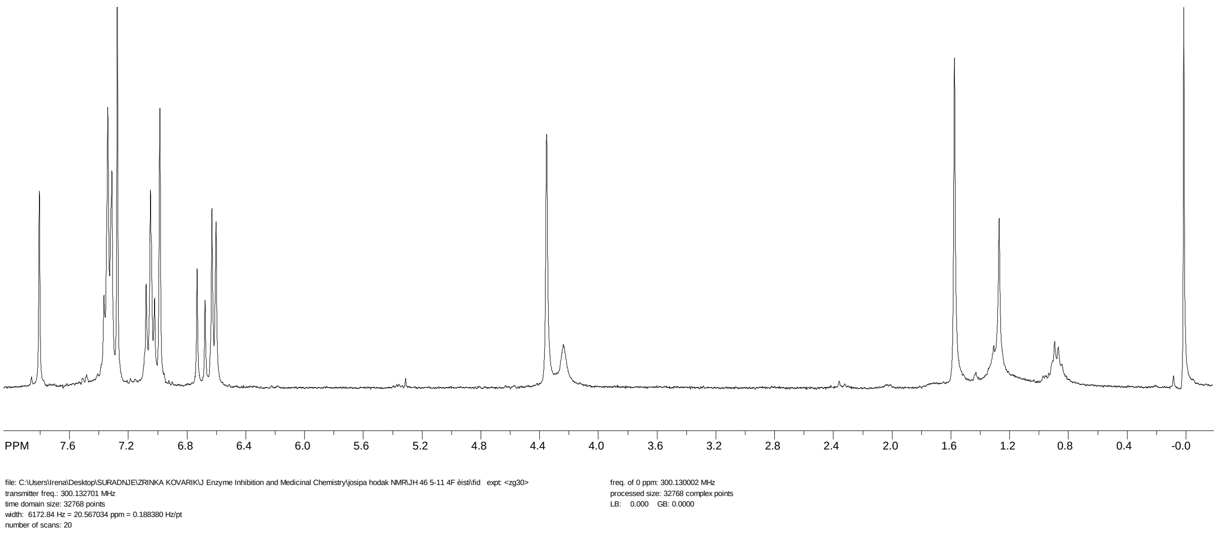

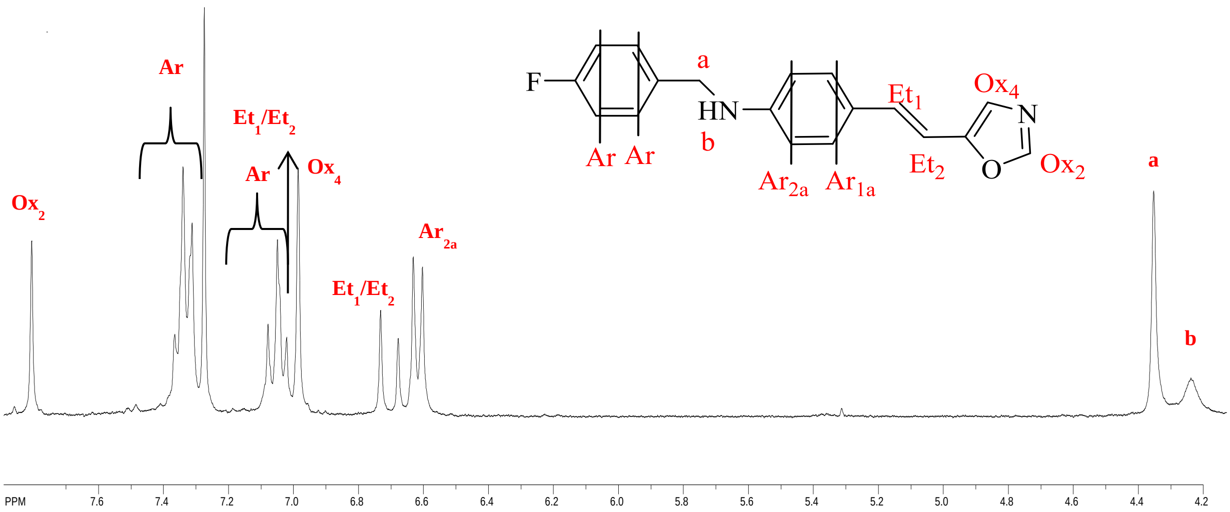

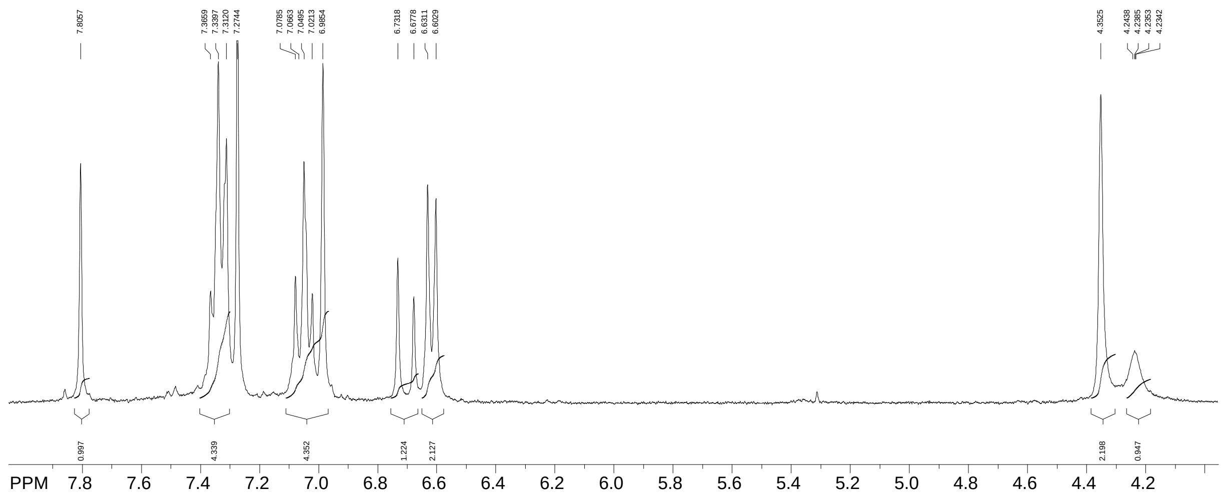
PPM
7.8
7.6
7.4
7.2
7.0
6.8
6.6
6.4
6.2
6.0
5.8
5.6
5.4
5.2
5.0
4.8
4.6
4.4
4.2

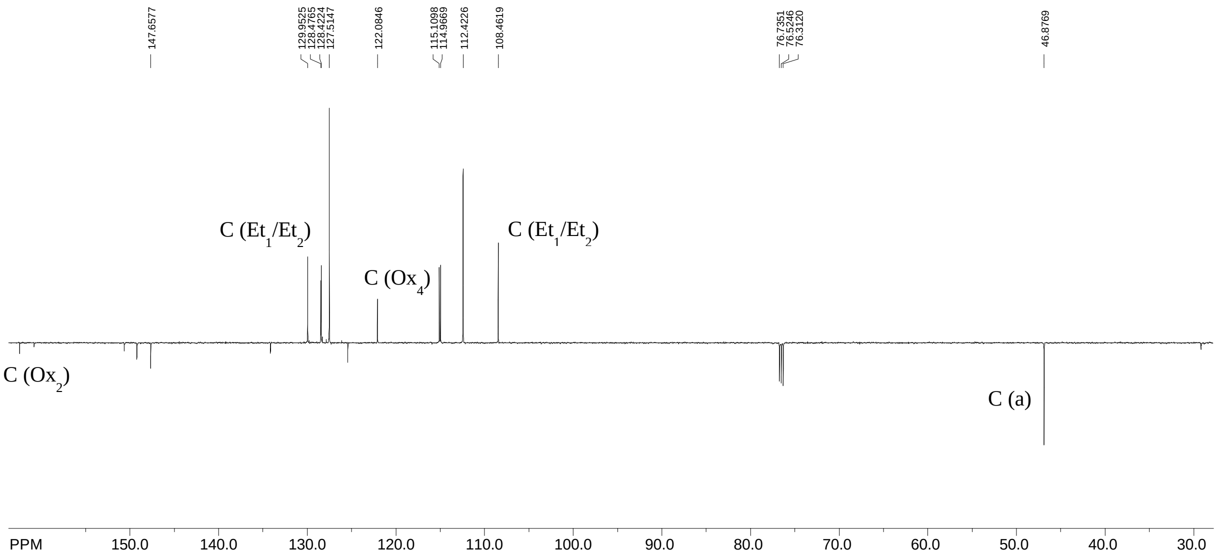
C (Et1/Et2)
C (Et1/Et2)
C (Ox4)
C (Ox2)
C (a)
PPM
150.0
140.0
130.0
120.0
110.0
100.0
90.0
80.0
70.0
60.0
50.0
40.0
30.0

Figure S6. ^{1}H and ^{13}C NMR spectra of (*E*)-*N*-(<u>3-methylbenzyl</u>)-4-(2-(oxazol-5-yl)vinyl)aniline (*trans*-**7**)

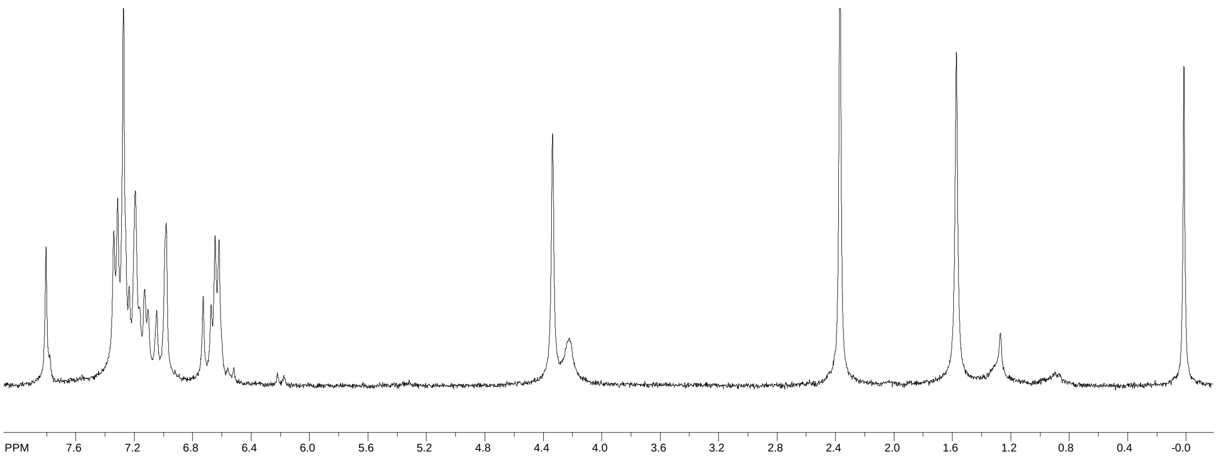

file: C:\Users\Irena\Desktop\SURADNJE\ZRINKA KOVARIK\J Enzyme Inhibition and Medicinal Chemistry\josipa hodak NMR\JH 50 4-5 3Me èisti\fid expt: <zg30>
transmitter freq.: 300.132701 MHz
time domain size: 32768 points
width: 6172.84 Hz = 20.567034 ppm = 0.188380 Hz/pt
number of scans: 16

freq. of 0 ppm: 300.130002 MHz
processed size: 32768 complex points
LB: 0.000 GB: 0.0000

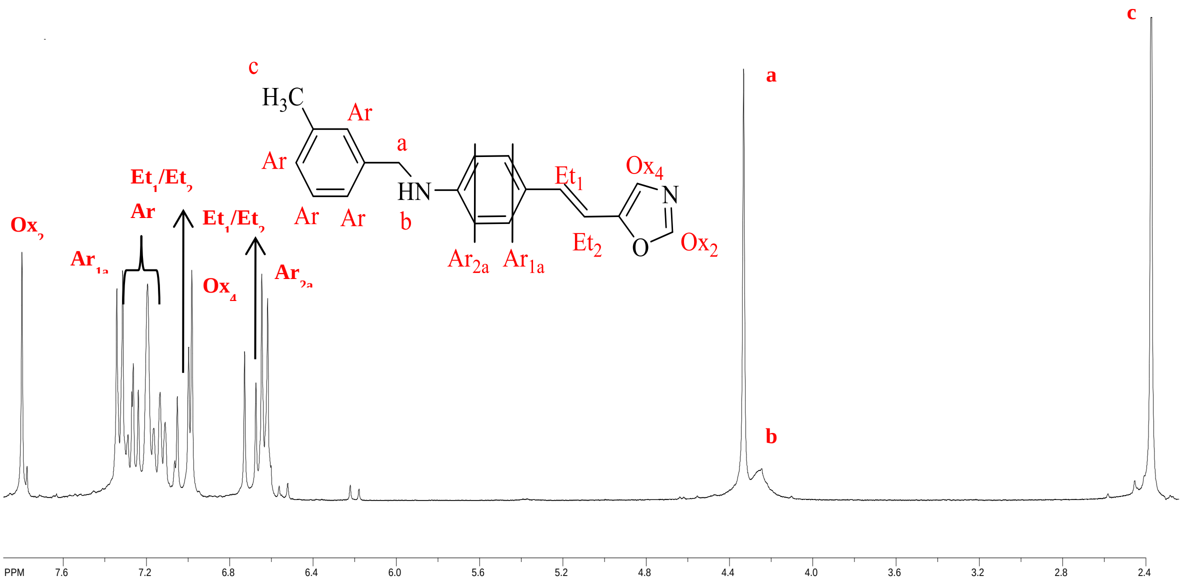

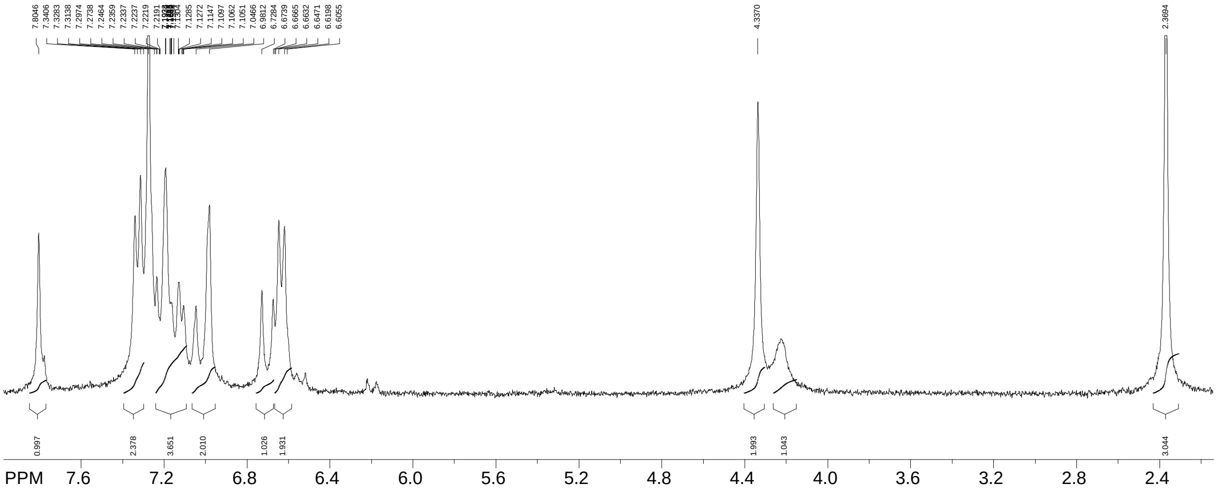
4.3370
2.3694
0.997
2.378
3.651
2.010
1.026
1.931
1.993
1.043
3.044
PPM 7.6 7.2 6.8 6.4 6.0 5.6 5.2 4.8 4.4 4.0 3.6 3.2 2.8 2.4

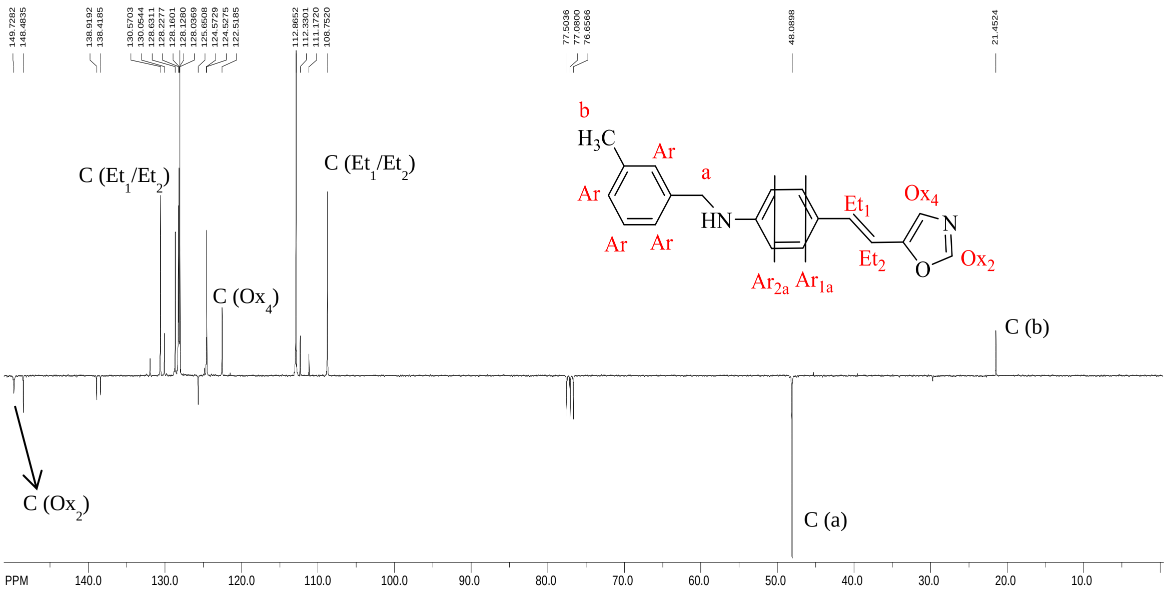
149.7282
148.4835
138.9192
138.4185
112.8652
108.7520
77.5036
77.0800
76.6566
48.0898
21.4524
C (Et1/Et2)
C (Et1/Et2)
C (Ox4)
C (Ox2)
C (a)
C (b)
b
H3C
Ar
Ar
Ar
Ar
a
HN
Et1
Et2
Ox4
N
O
Ox2
Ar2a Ar1a
PPM 140.0 130.0 120.0 110.0 100.0 90.0 80.0 70.0 60.0 50.0 40.0 30.0 20.0 10.0

Figure S7. COSY spectra of (*E*)-*N*-(3-methylbenzyl)-4-(2-(oxazol-5-y)vinyl)aniline (*trans*-**7**)

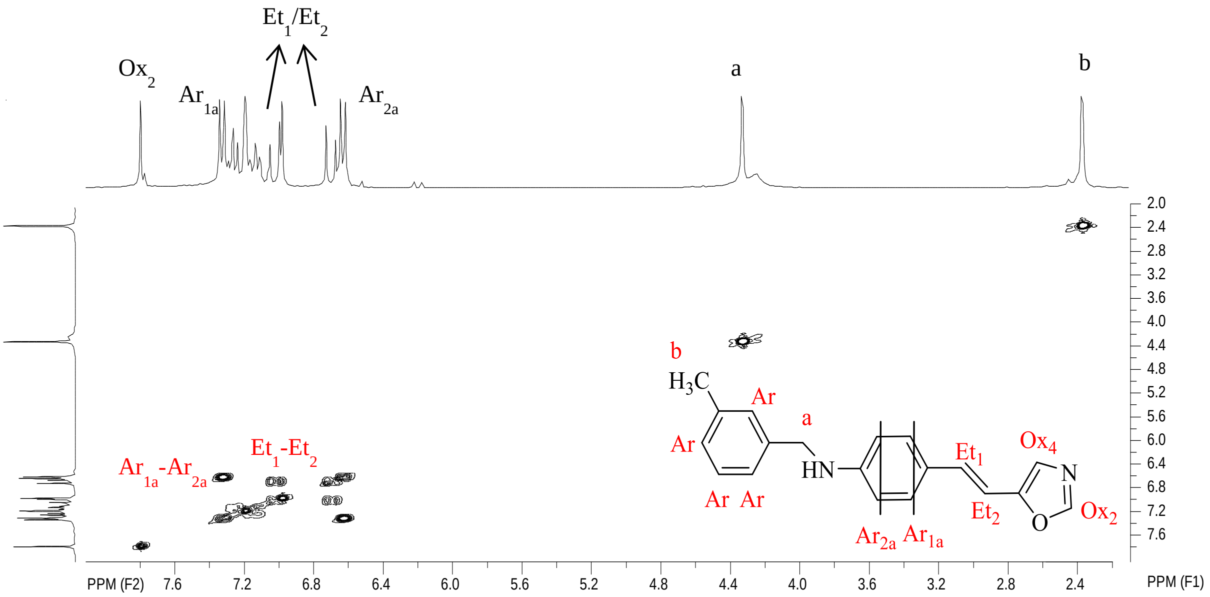

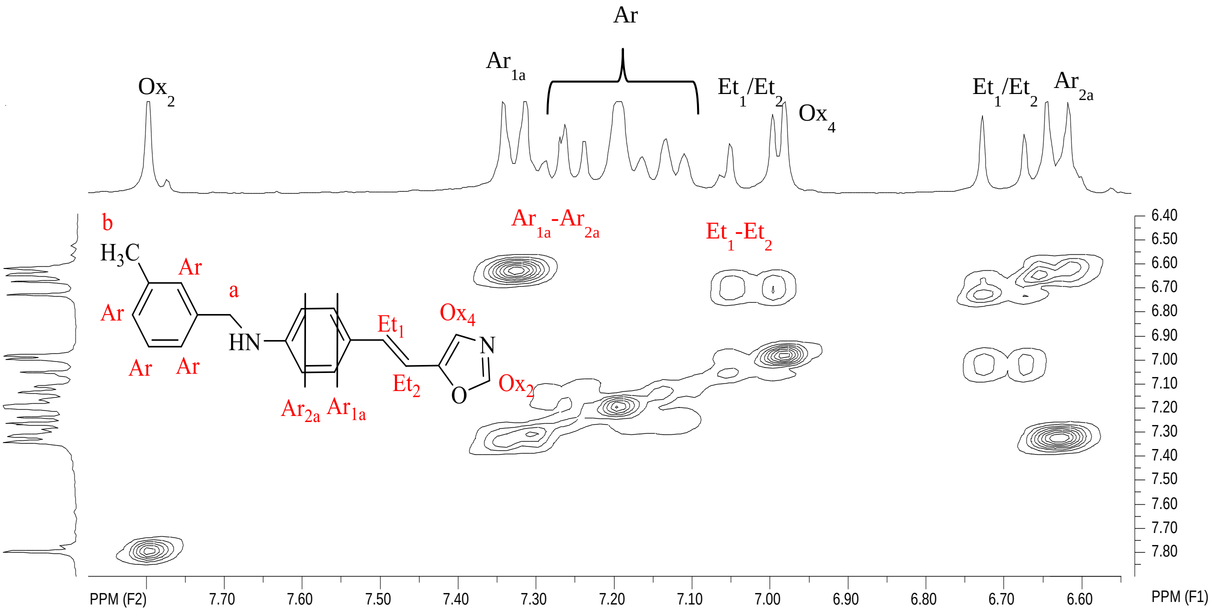

Figure S8. HETCOR spectra of (*E*)-*N*-(3-methylbenzyl)-4-(2-(oxazol-5-il)vinil)aniline (*trans*-**7**)

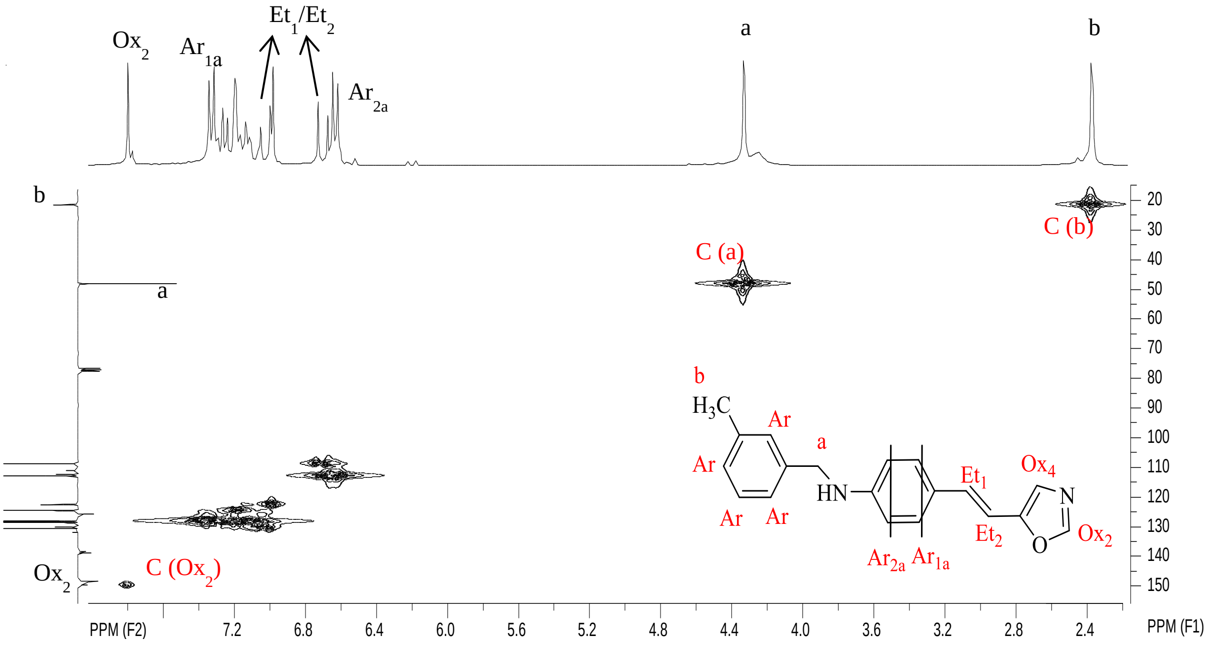

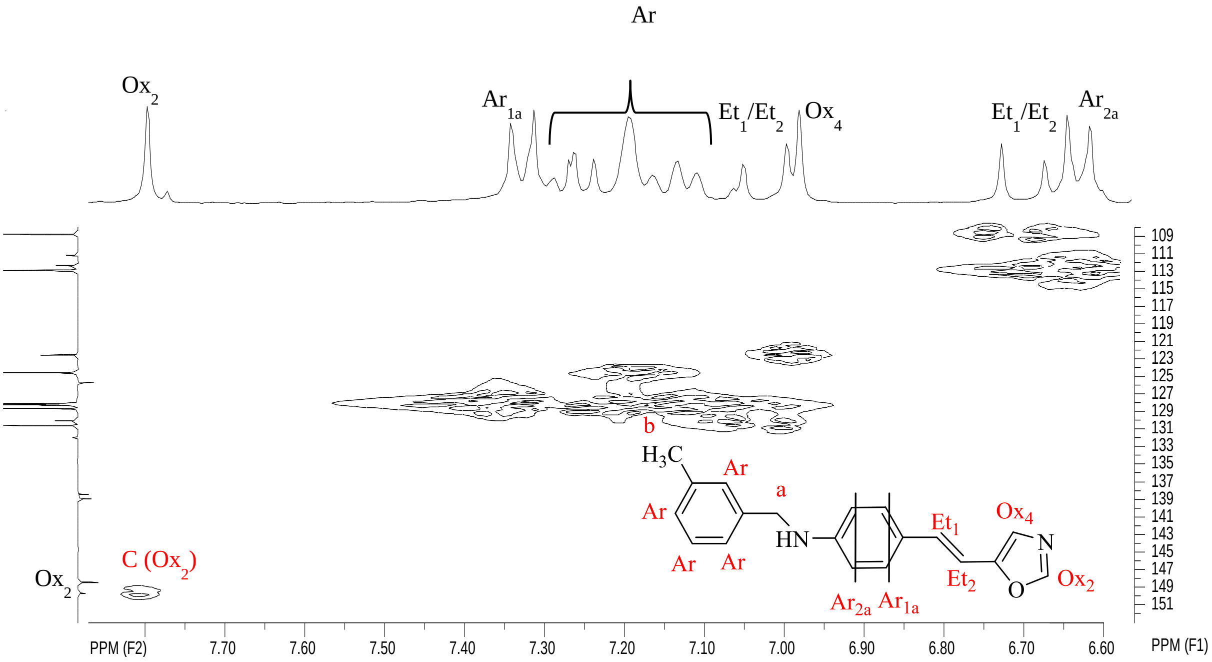

Figure S9. NOESY spectra of (*E*)-*N*-(3-methylbenzyl)-4-(2-(oxazol-5-yl)vinyl)aniline (*trans*-**7**)

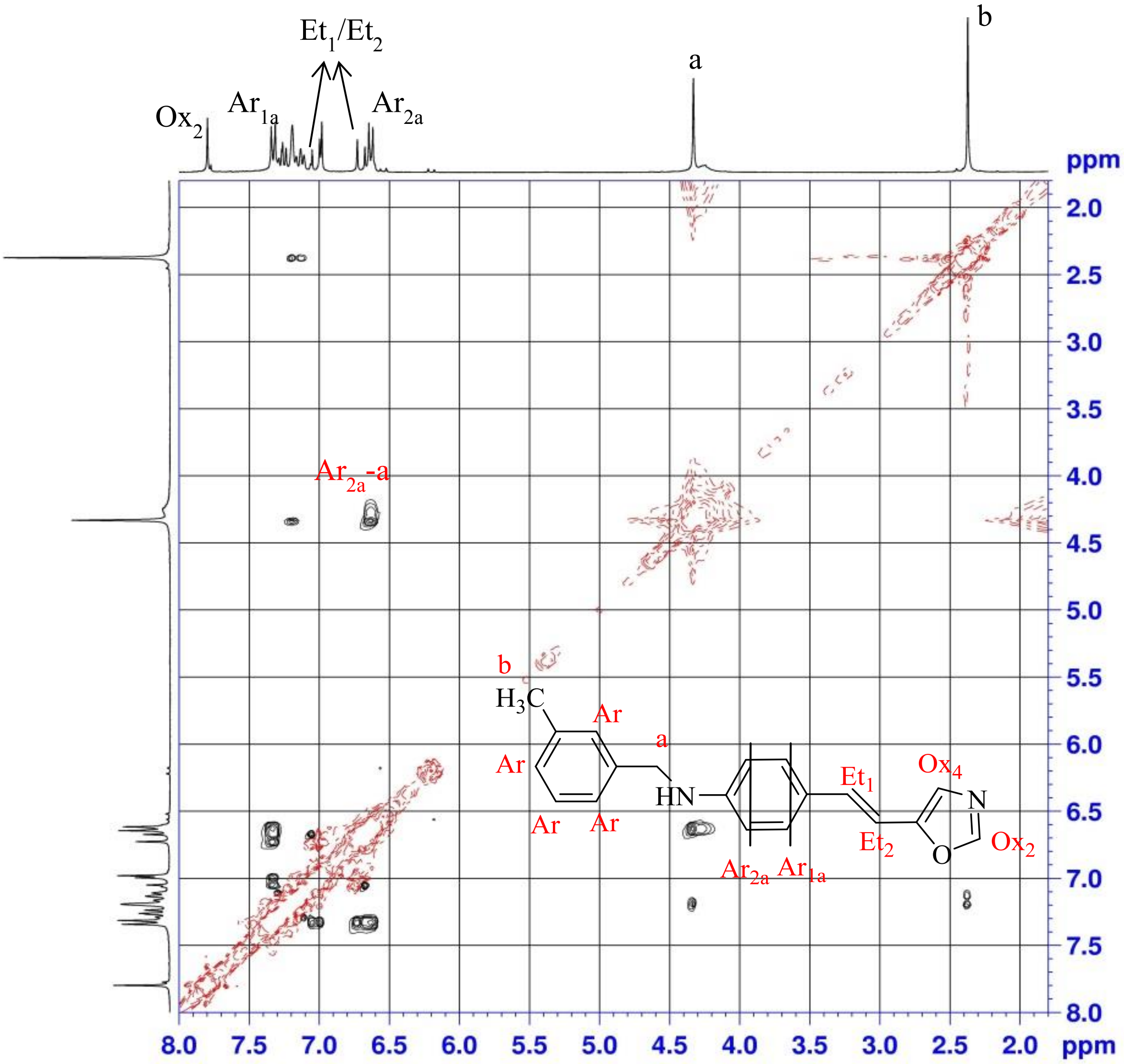

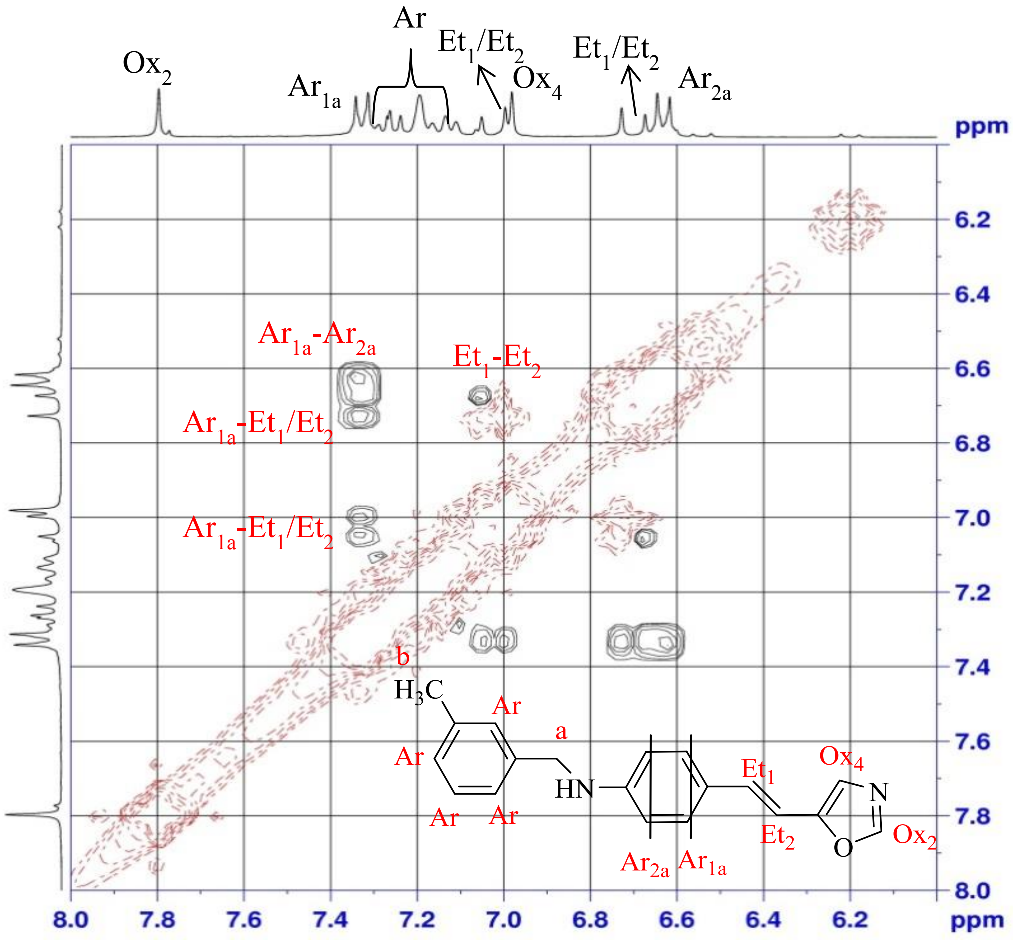
Ox2
Ar1a
Ar
Et1/Et2
Ox4
Et1/Et2
Ar2a
ppm
Ar1a-Ar2a
Et1-Et2
Ar1a-Et1/Et2
Ar1a-Et1/Et2
b
H3C
Ar
a
Ar
HN
Et1
Ox4
N
Ar
Ar
Et2
O
Ox2
Ar2a
Ar1a
6.2
6.4
6.6
6.8
7.0
7.2
7.4
7.6
7.8
8.0
8.0
7.8
7.6
7.4
7.2
7.0
6.8
6.6
6.4
6.2
ppm

Figure S10. ^{1}H and ^{13}C NMR spectra of (*E*)-*N*-(3-methoxybenzyl)-4-(2-(oxazol-5-yl)vinyl)aniline (*trans*-**8**)

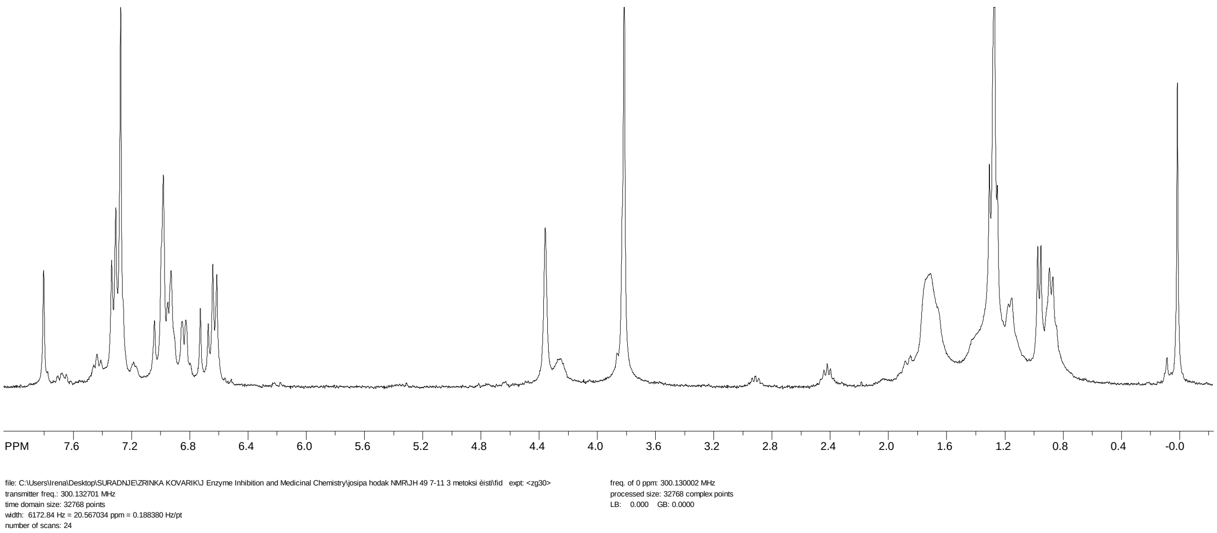

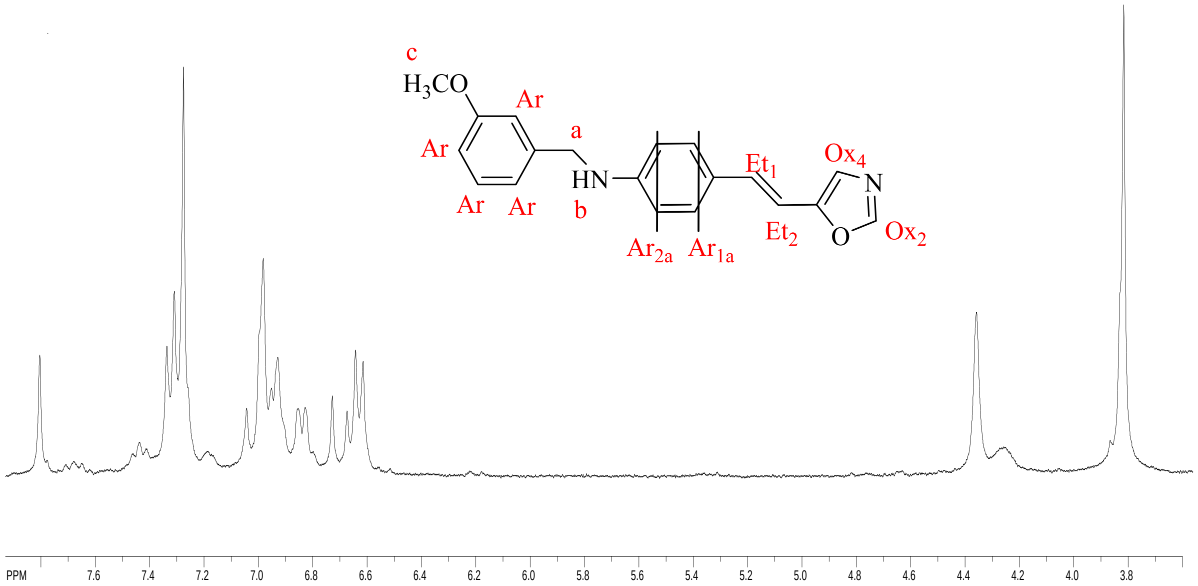

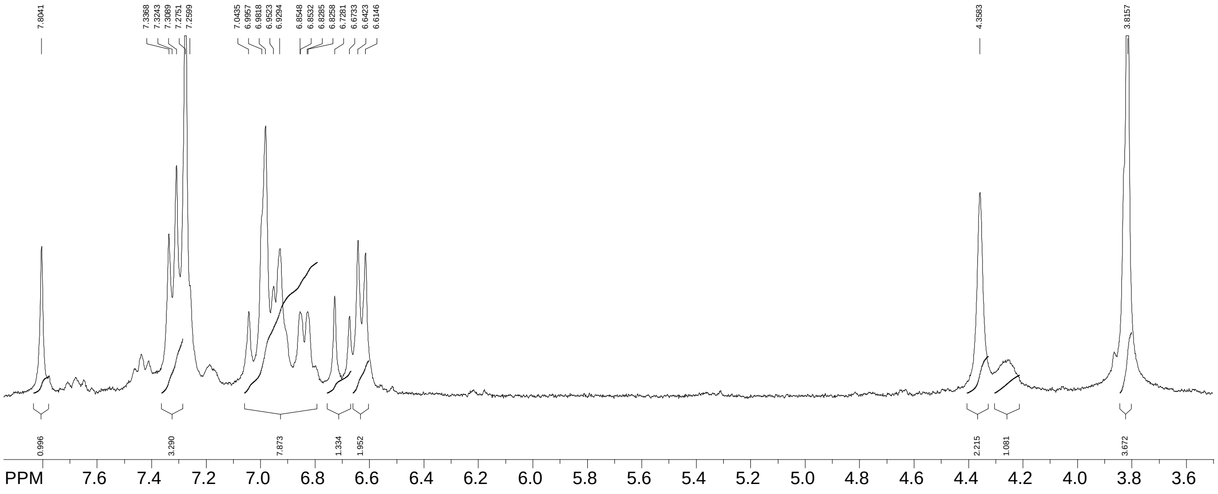
PPM
7.6
7.4
7.2
7.0
6.8
6.6
6.4
6.2
6.0
5.8
5.6
5.4
5.2
5.0
4.8
4.6
4.4
4.2
4.0
3.8
3.6

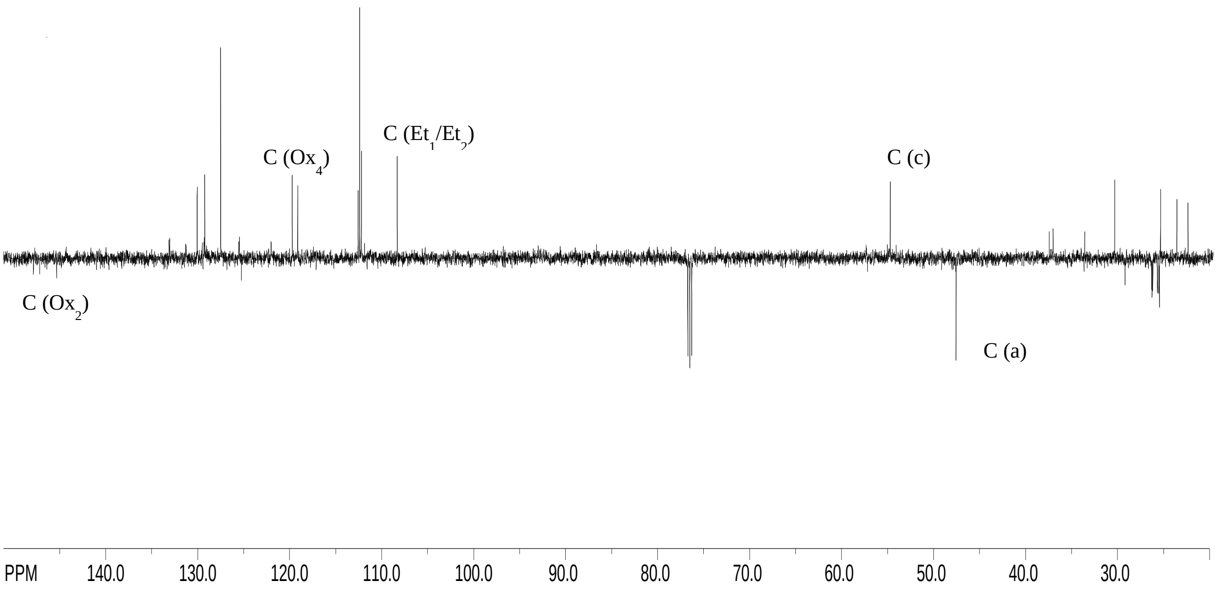
C (Ox$_4$)
C (Et$_1$/Et$_2$)
C (c)
C (Ox$_2$)
C (a)
PPM
140.0
130.0
120.0
110.0
100.0
90.0
80.0
70.0
60.0
50.0
40.0
30.0

Figure S11. ^{1}H and ^{13}C NMR spectra of (*E*)-*N*-(<u>3-chlorobenzyl</u>)-4-(2-(oxazol-5-yl)vinyl)aniline (*trans*-**9**)

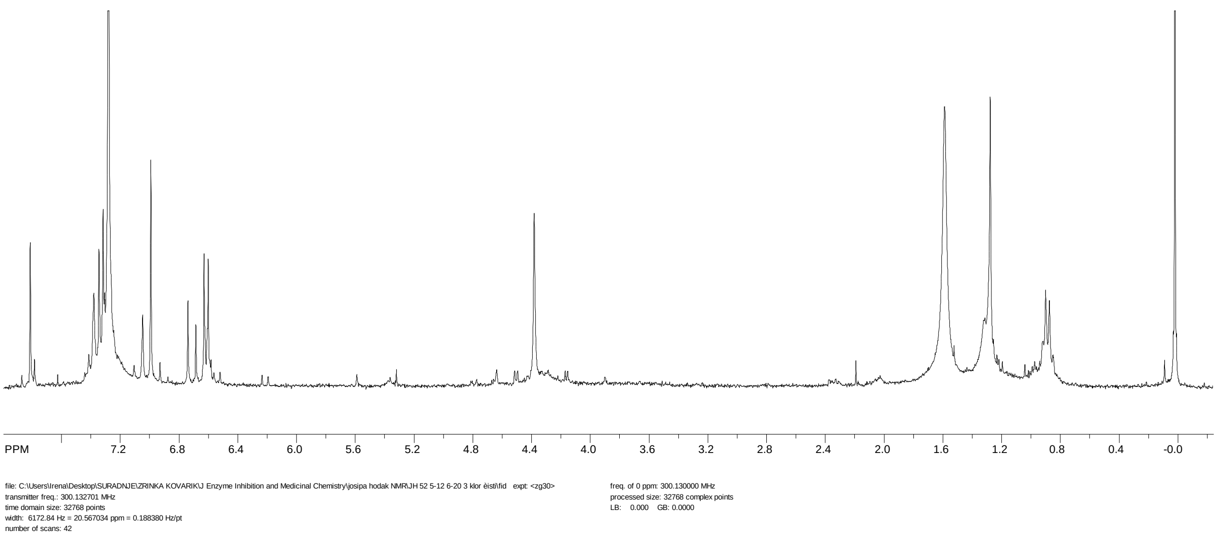

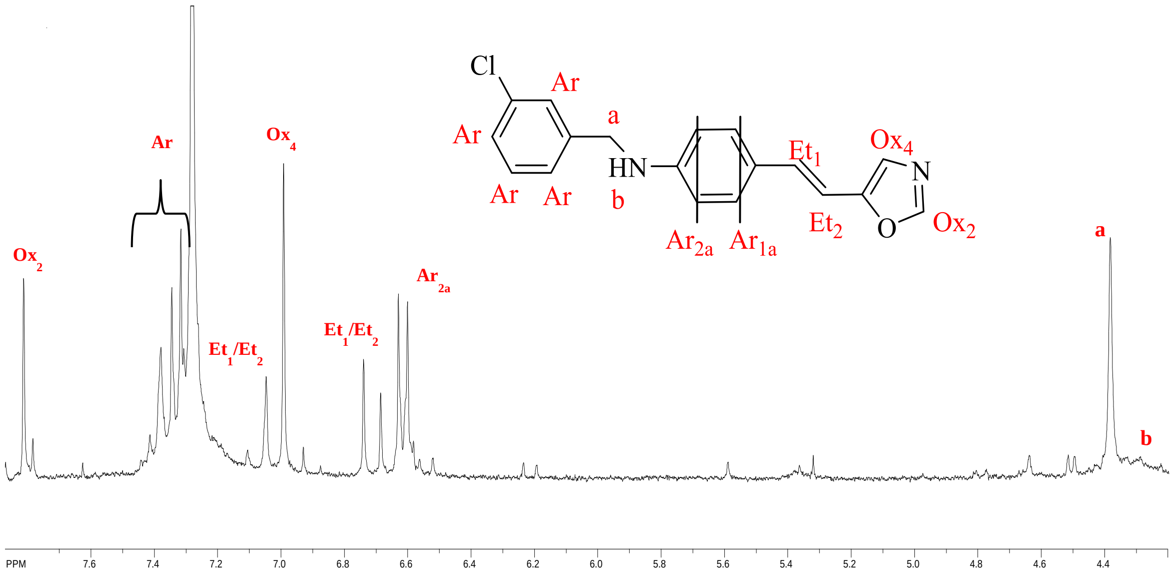

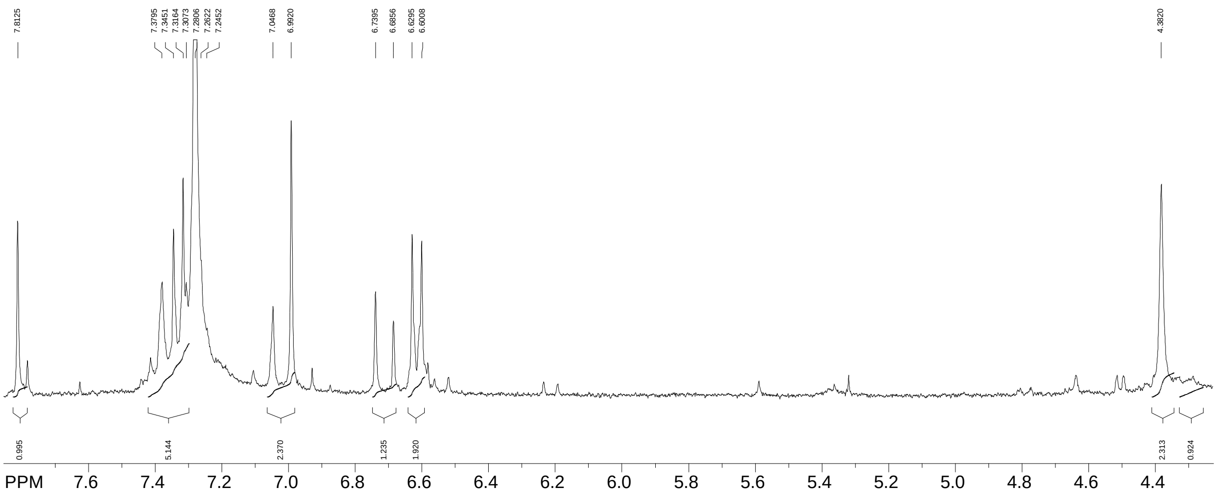
7.8125
7.3795
7.3451
7.3164
7.3073
7.2806
7.2622
7.2452
7.0468
6.9920
6.7395
6.6856
6.6295
6.6008
4.3820
0.995
5.144
2.370
1.235
1.920
2.313
0.924
PPM
7.6
7.4
7.2
7.0
6.8
6.6
6.4
6.2
6.0
5.8
5.6
5.4
5.2
5.0
4.8
4.6
4.4

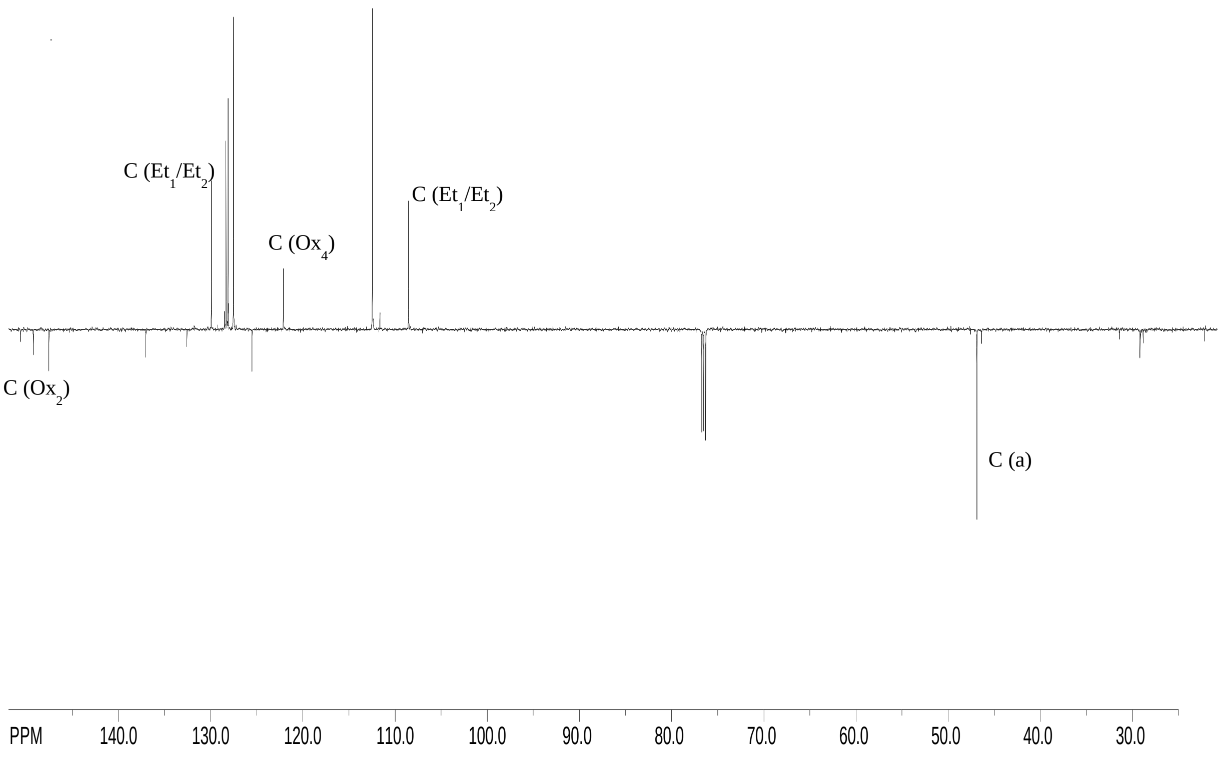
C (Et1/Et2)
C (Et1/Et2)
C (Ox4)
C (Ox2)
C (a)
PPM
140.0
130.0
120.0
110.0
100.0
90.0
80.0
70.0
60.0
50.0
40.0
30.0

Figure S12. ^{1}H and ^{13}C NMR spectra of (*E*)-*N*-(3-fluorobenzyl)-4-(2-(oxazol-5-yl)vinyl)aniline (*trans*-**10**)

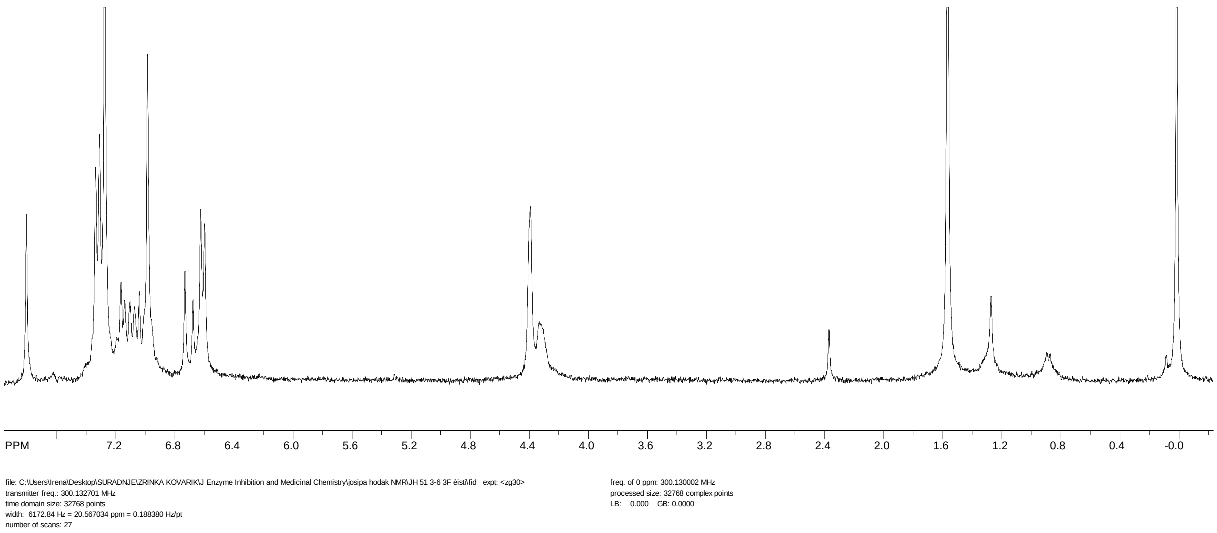

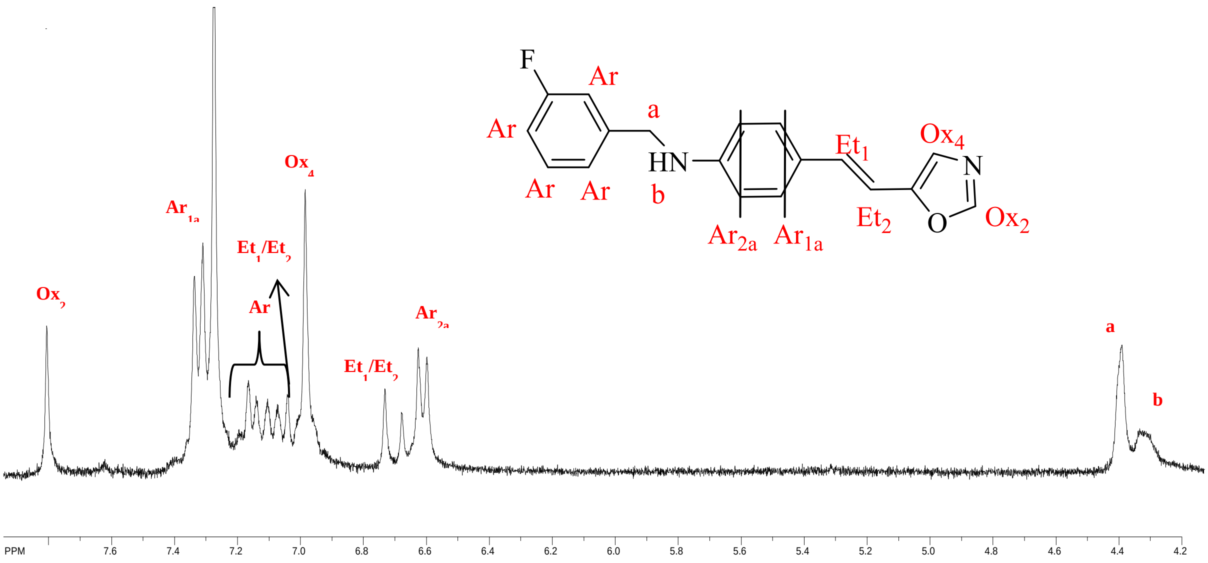

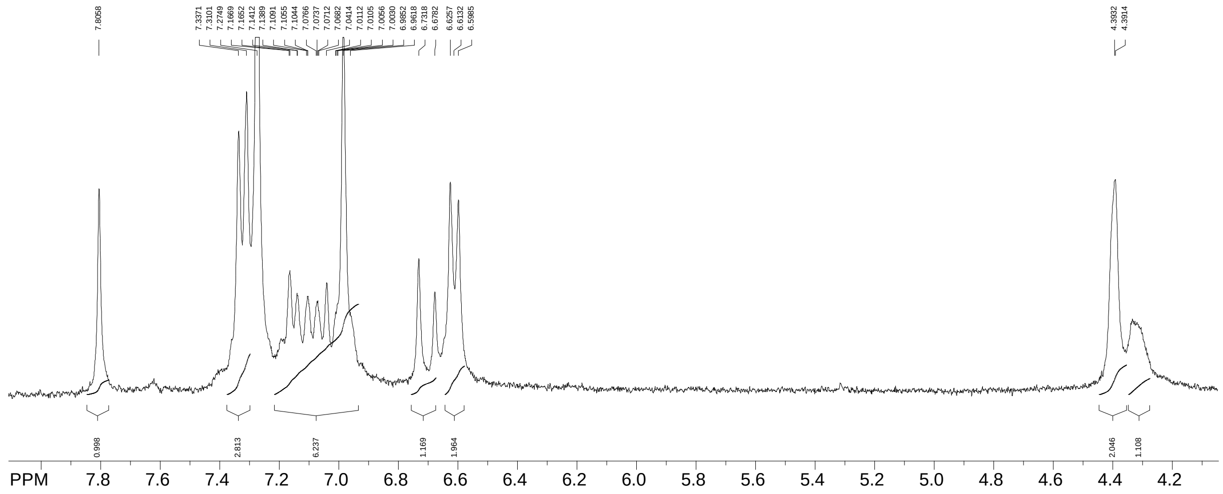
7.8058
4.3932
4.3914
0.998
2.813
6.237
1.169
1.964
2.046
1.108
PPM 7.8 7.6 7.4 7.2 7.0 6.8 6.6 6.4 6.2 6.0 5.8 5.6 5.4 5.2 5.0 4.8 4.6 4.4 4.2

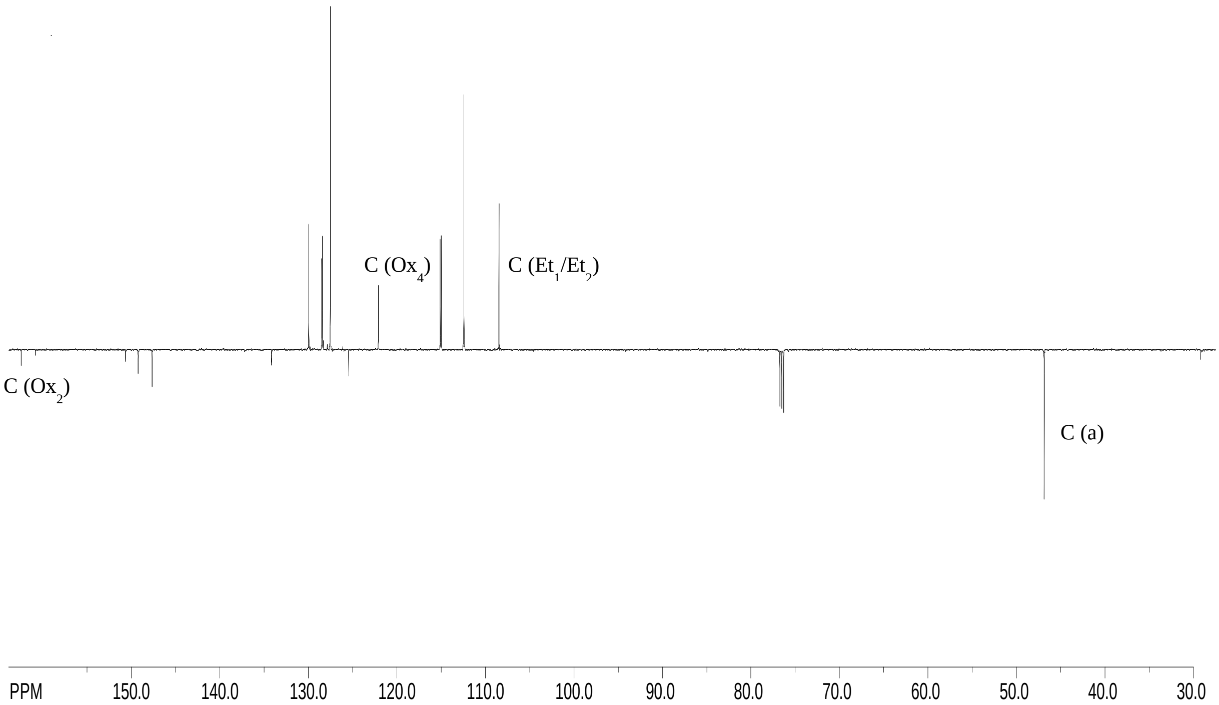
C (Ox$_4$)
C (Et$_1$/Et$_2$)
C (Ox$_2$)
C (a)
PPM 150.0 140.0 130.0 120.0 110.0 100.0 90.0 80.0 70.0 60.0 50.0 40.0 30.0

Figure S13. ^{1}H and ^{13}C NMR spectra of (*E*)-*N*-(<u>2-methylbenzyl</u>)-4-(2-(oxazol-5-yl)vinyl)aniline (*trans*-**11**)

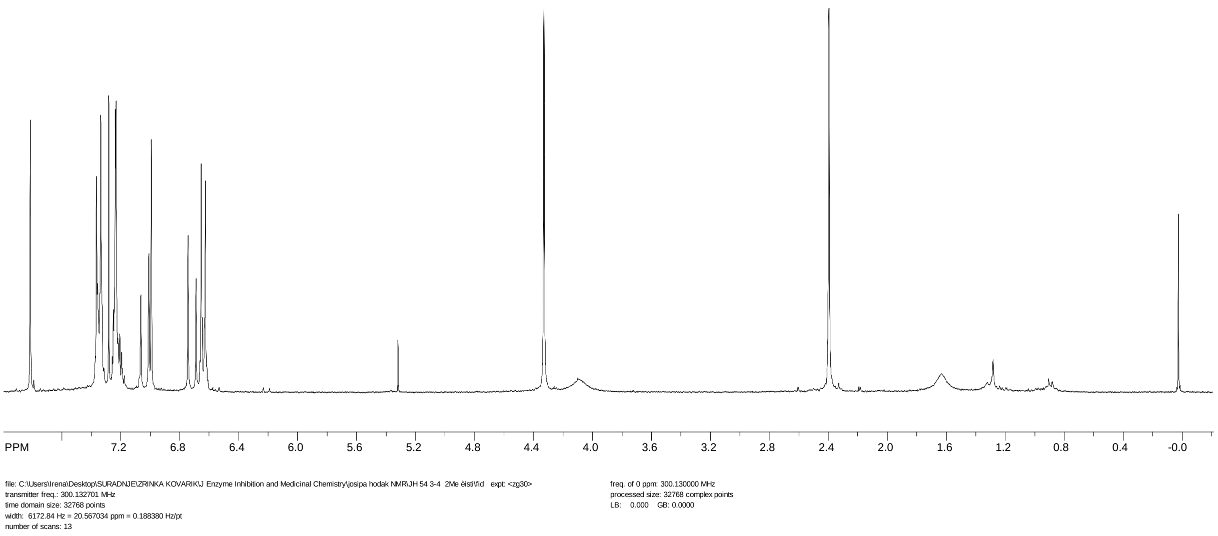

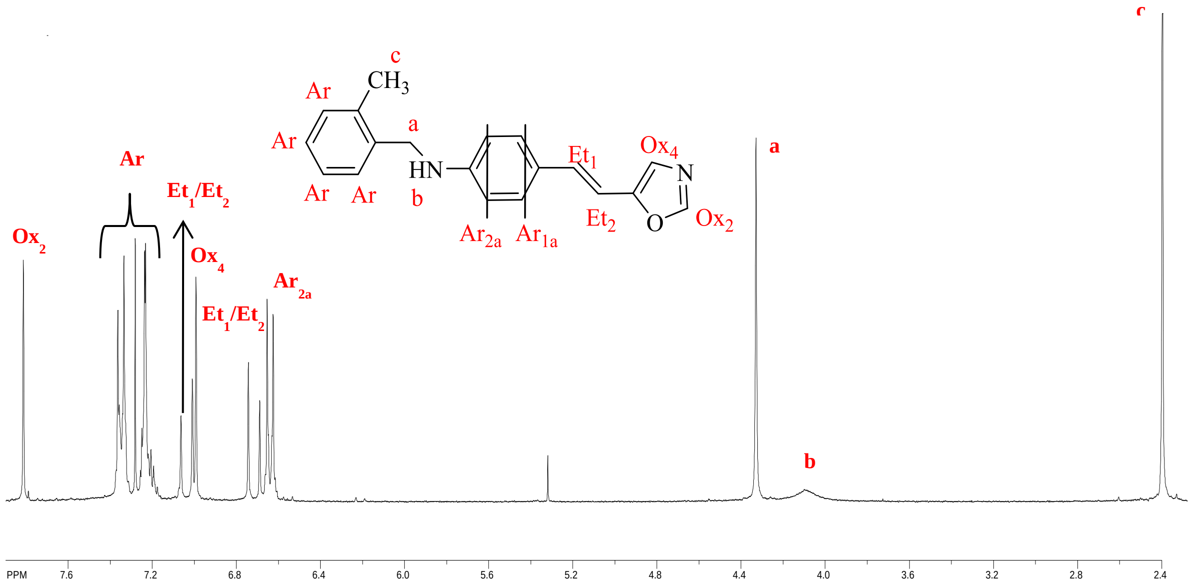

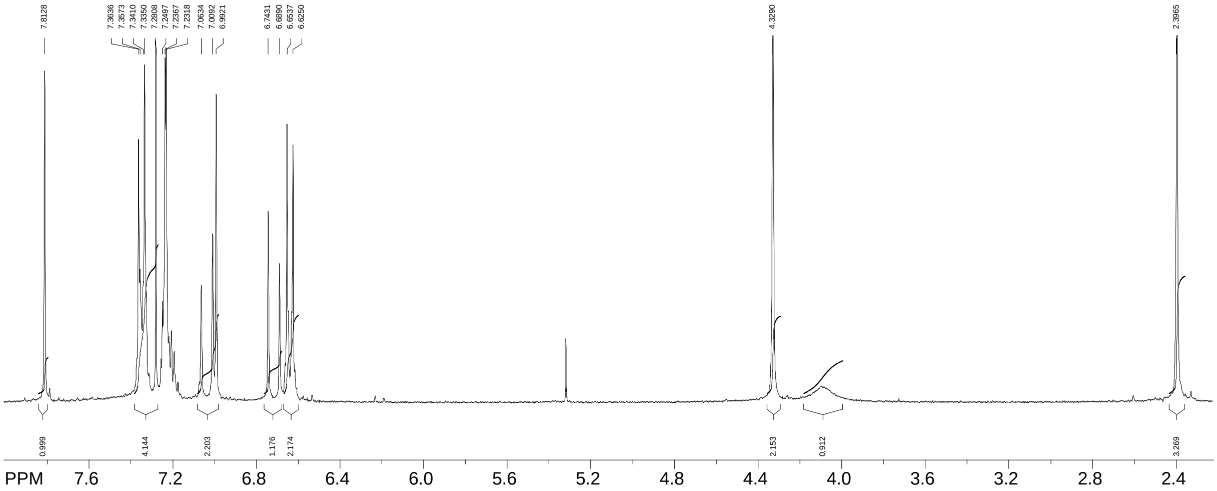
7.8128
7.3636
7.3573
7.3410
7.3350
7.2808
7.2497
7.2367
7.2318
7.0634
7.0092
6.9921
6.7431
6.6890
6.6537
6.6250
4.3290
2.3965
0.999
4.144
2.203
1.176
2.174
2.153
0.912
3.269
PPM 7.6 7.2 6.8 6.4 6.0 5.6 5.2 4.8 4.4 4.0 3.6 3.2 2.8 2.4

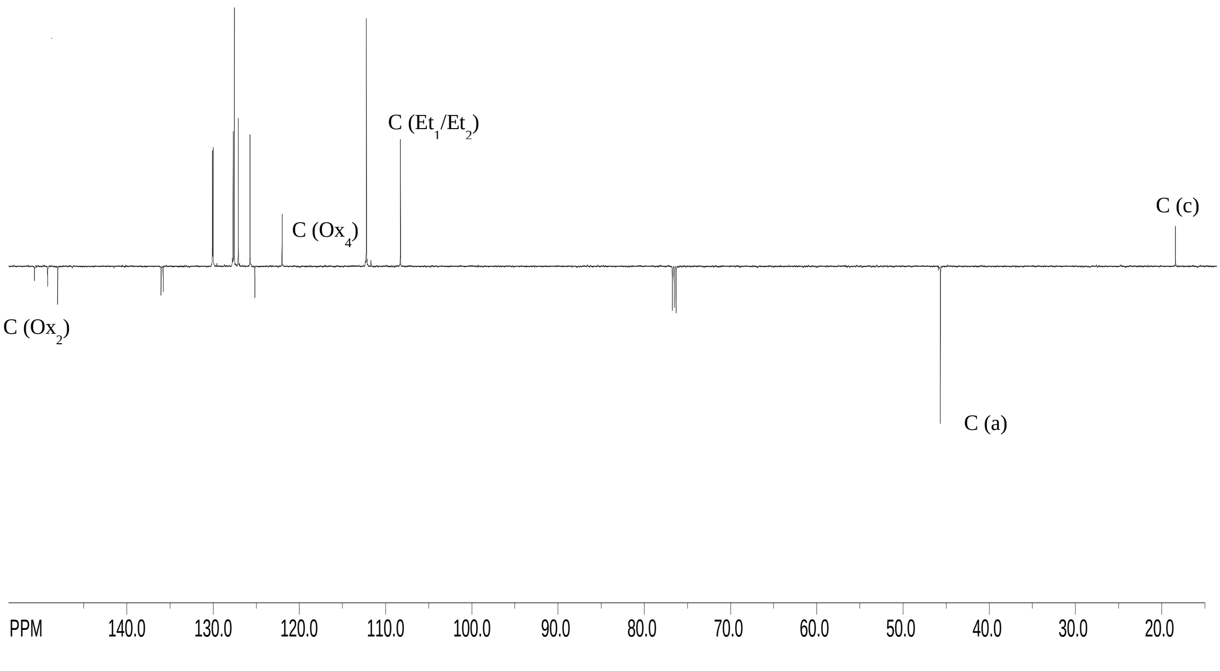
C (Et1/Et2)
C (c)
C (Ox4)
C (Ox2)
C (a)
PPM 140.0 130.0 120.0 110.0 100.0 90.0 80.0 70.0 60.0 50.0 40.0 30.0 20.0

Figure S14. ^{1}H and ^{13}C NMR spectra of (*E*)-*N*-(2-methoxybenzyl)-4-(2-(oxazol-5-yl)vinyl)aniline (*trans*-**12**)

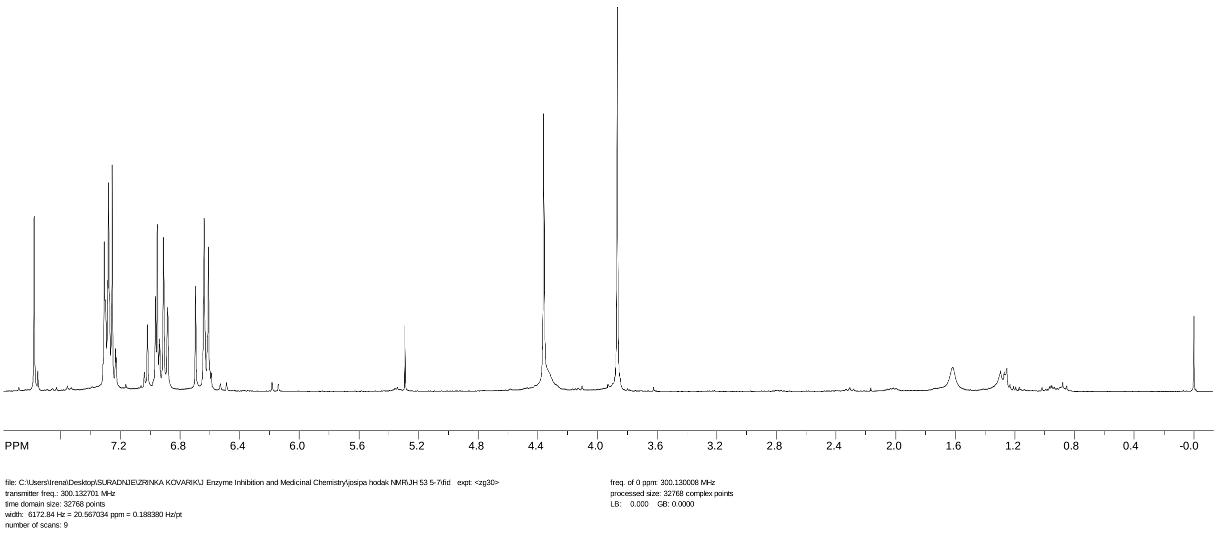

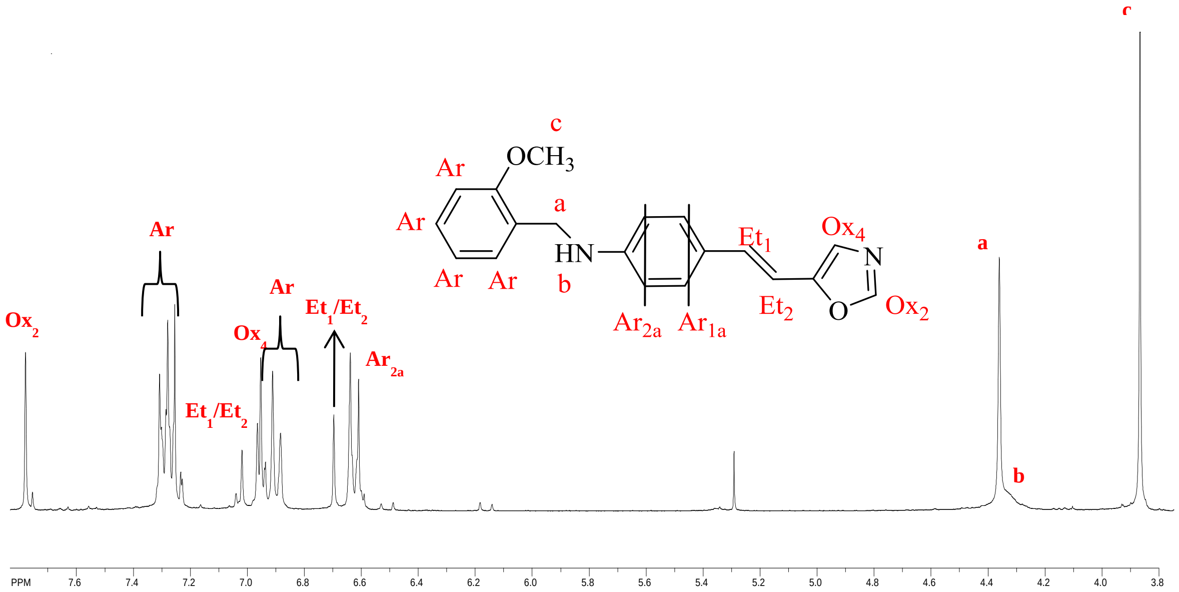

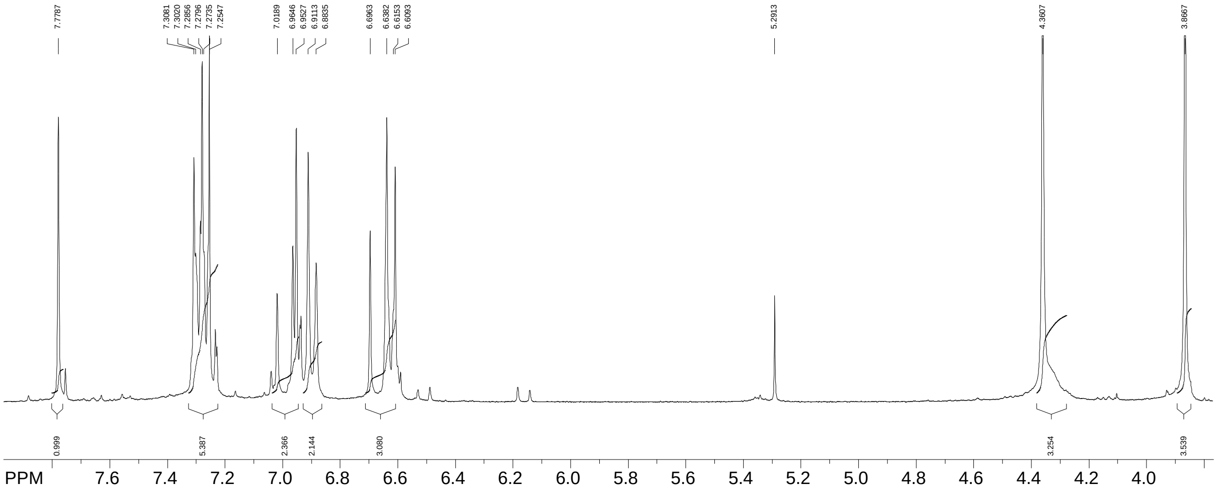
7.7787
7.3081
7.3020
7.2856
7.2796
7.2735
7.2547
7.0189
6.9646
6.9527
6.9113
6.8835
6.6963
6.6382
6.6153
6.6093
5.2913
4.3607
3.8667
0.999
5.387
2.366
2.144
3.080
3.254
3.539
PPM
7.6
7.4
7.2
7.0
6.8
6.6
6.4
6.2
6.0
5.8
5.6
5.4
5.2
5.0
4.8
4.6
4.4
4.2
4.0

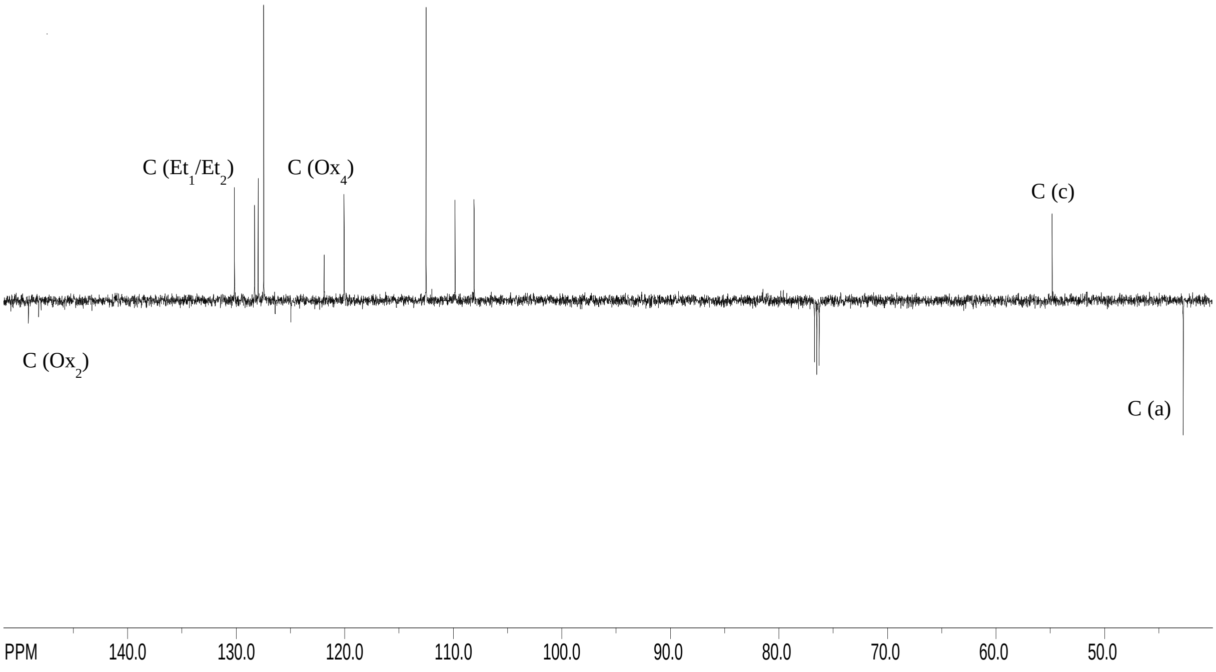
C (Et1/Et2)
C (Ox4)
C (c)
C (Ox2)
C (a)
PPM
140.0
130.0
120.0
110.0
100.0
90.0
80.0
70.0
60.0
50.0

Figure S15. ^{1}H and ^{13}C NMR spectra of (*E*)-*N*-(2-fluorobenzyl)-4-(2-(oxazol-5-yl)vinyl)aniline (*trans*-**14**)

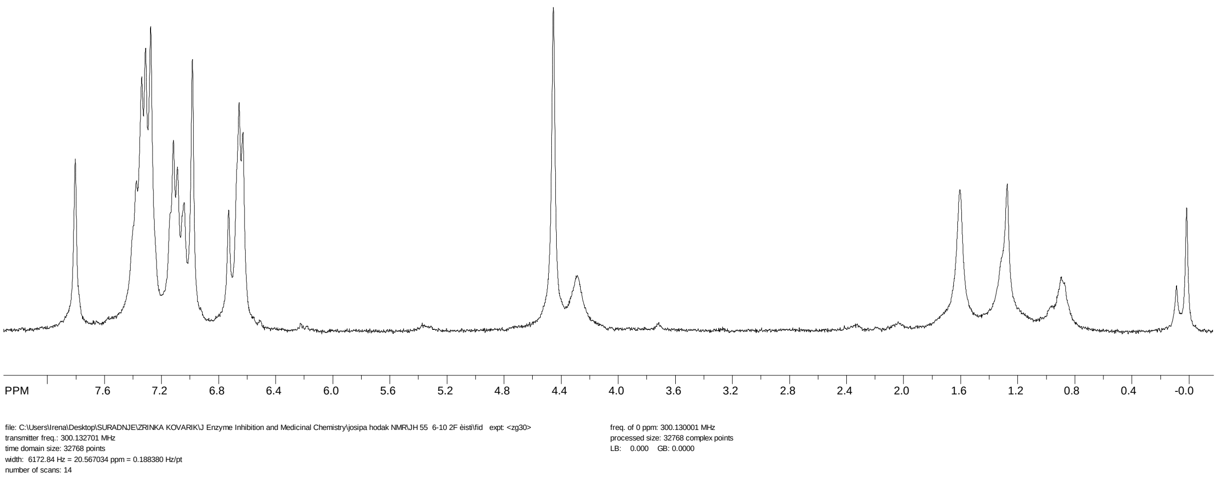

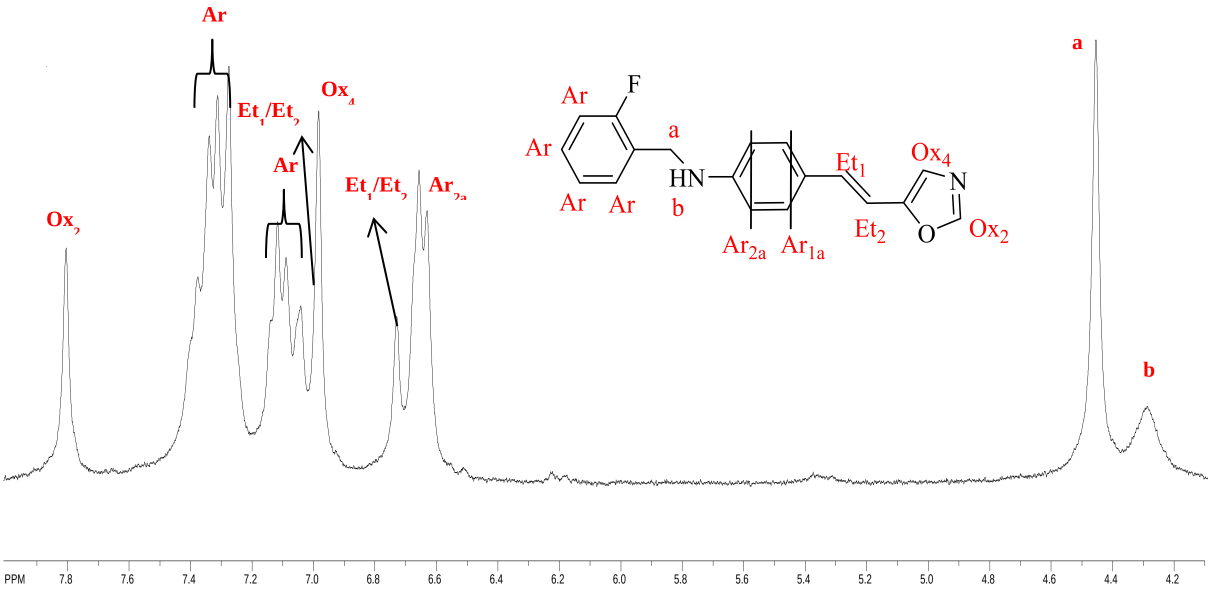

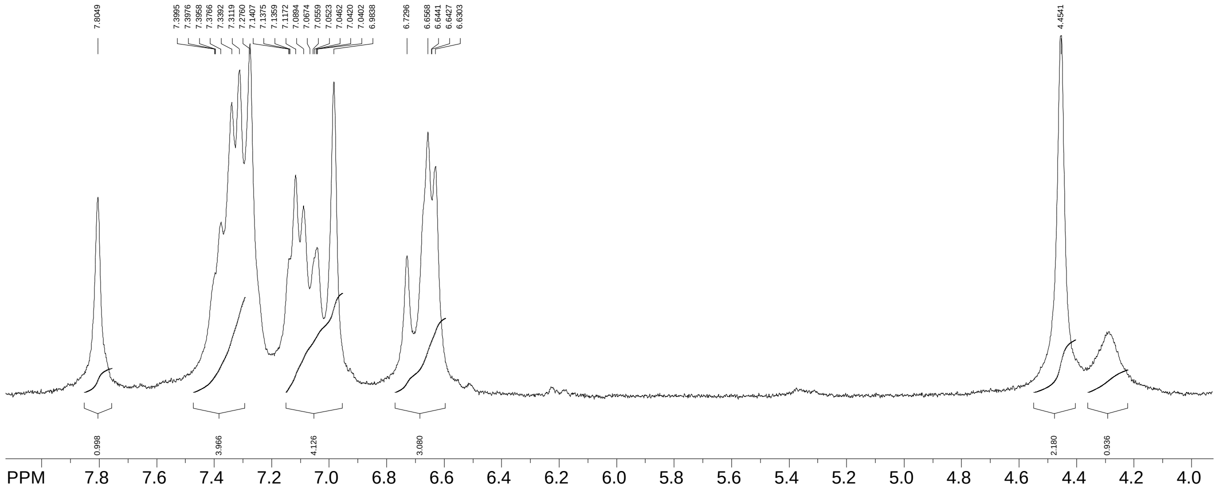
7.8049
7.3995
7.3976
7.3958
7.3766
7.3392
7.3119
7.2760
7.1407
7.1375
7.1359
7.1172
7.0894
7.0674
7.0559
7.0523
7.0462
7.0420
7.0402
6.9838
6.7296
6.6568
6.6441
6.6427
6.6303
4.4541
0.998
3.966
4.126
3.080
2.180
0.936
PPM 7.8 7.6 7.4 7.2 7.0 6.8 6.6 6.4 6.2 6.0 5.8 5.6 5.4 5.2 5.0 4.8 4.6 4.4 4.2 4.0

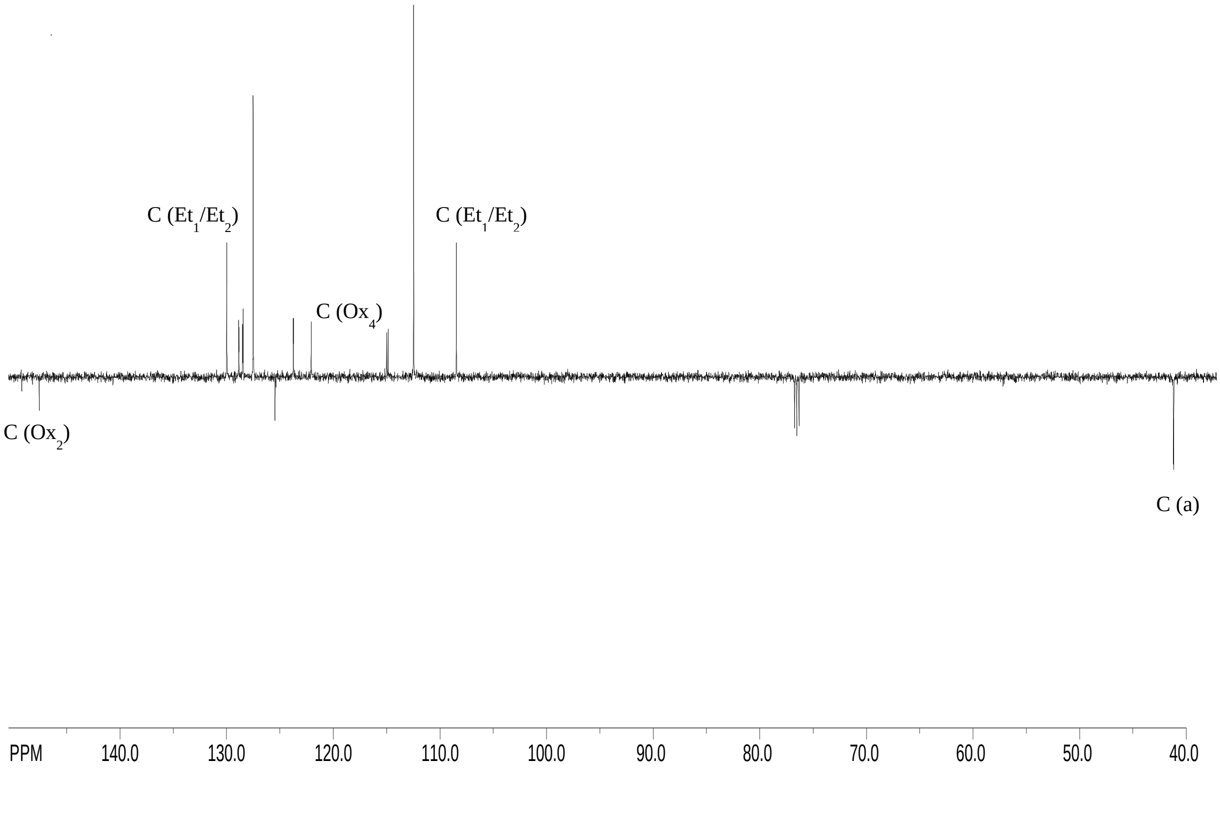
C (Et1/Et2)
C (Et1/Et2)
C (Ox4)
C (Ox2)
C (a)
PPM 140.0 130.0 120.0 110.0 100.0 90.0 80.0 70.0 60.0 50.0 40.0

Figure S16. ^{1}H and ^{13}C NMR spectra of (*E*)-*N*-(<u>furan-2-ylmethyl</u>)-4-(2-(oxazol-5-yl)vinyl)aniline (*trans*-**17**)

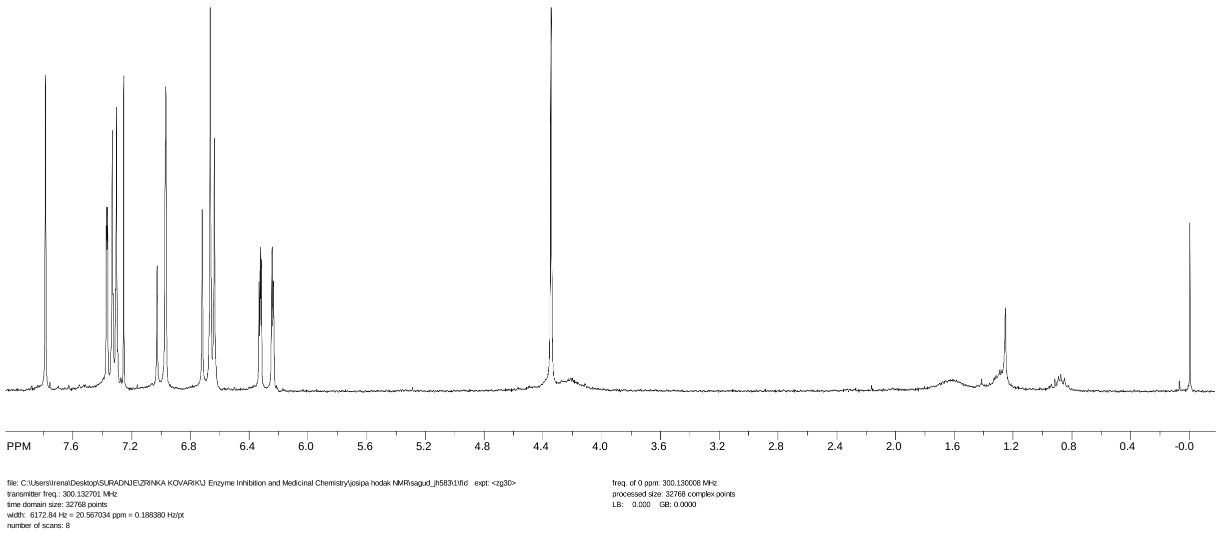

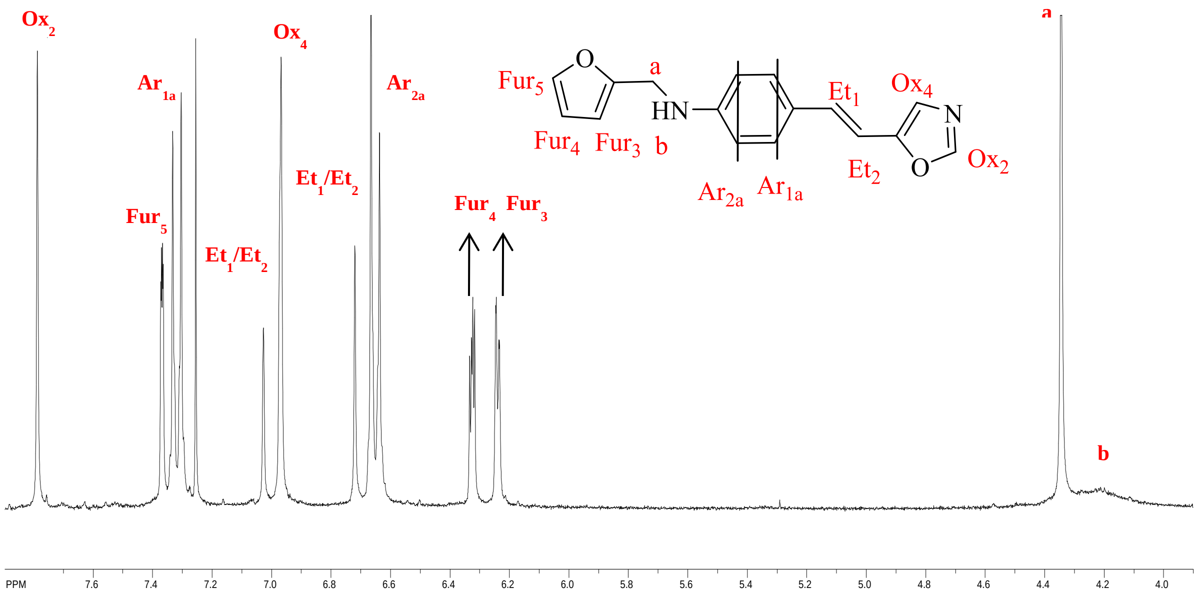

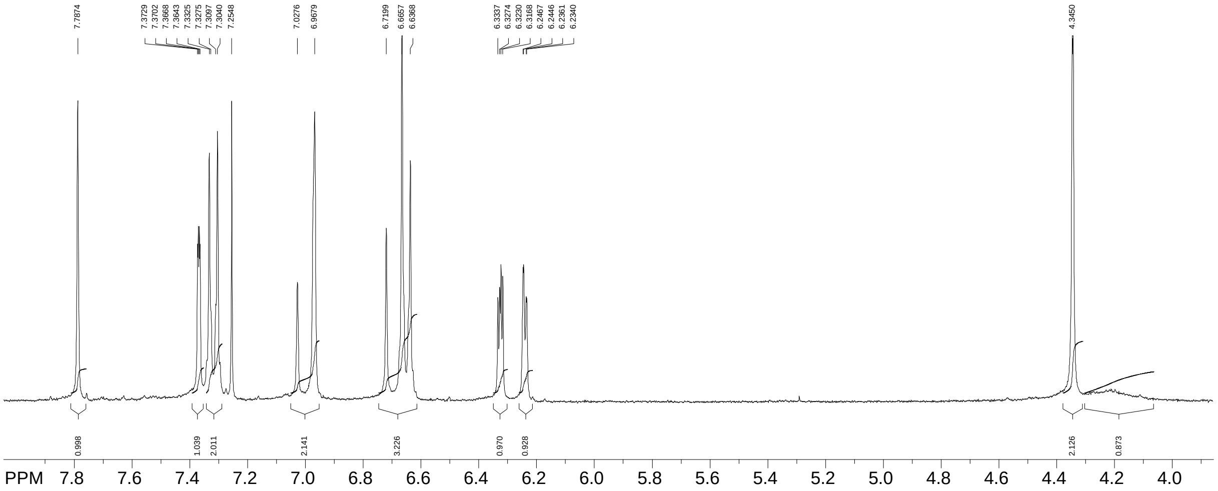
7.7874
7.3729 7.3702 7.3668 7.3643 7.3325 7.3275 7.3097 7.3040 7.2548
7.0276 6.9679
6.7199 6.6657 6.6368
6.3337 6.3274 6.3230 6.3168 6.2467 6.2446 6.2361 6.2340
4.3450
0.998 1.039 2.011 2.141 3.226 0.970 0.928 2.126 0.873
PPM 7.8 7.6 7.4 7.2 7.0 6.8 6.6 6.4 6.2 6.0 5.8 5.6 5.4 5.2 5.0 4.8 4.6 4.4 4.2 4.0

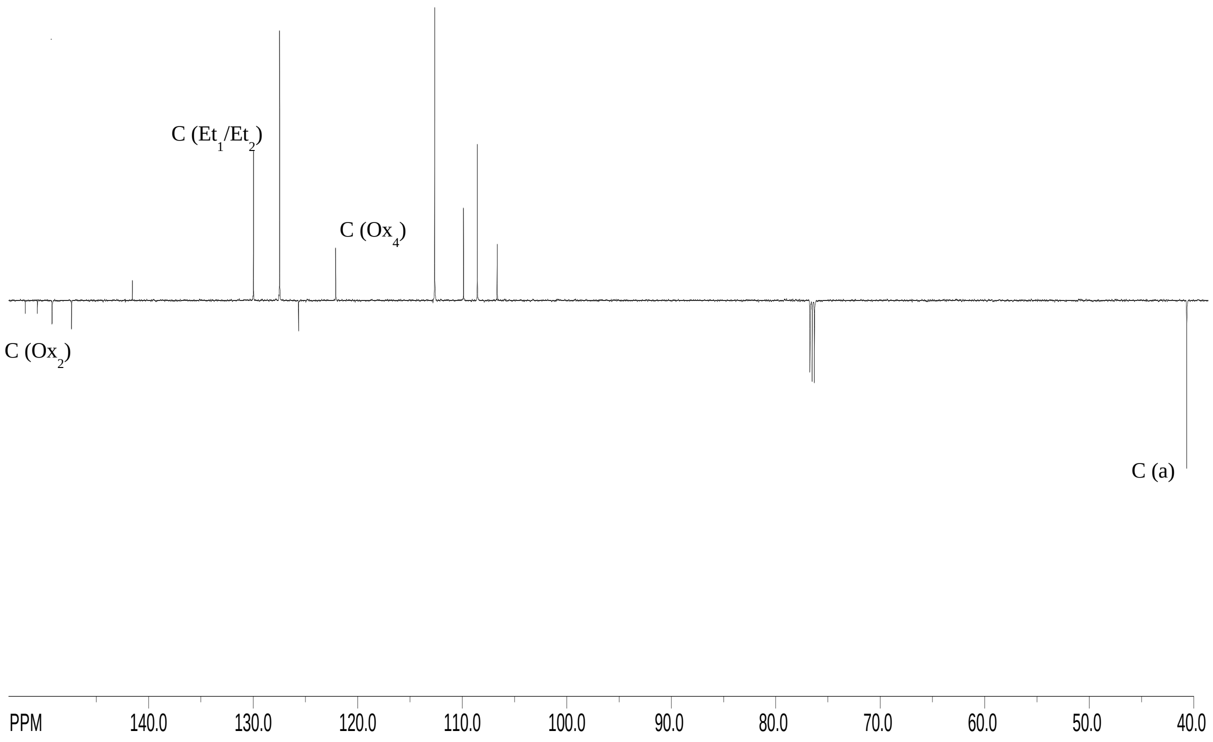
C (Et1/Et2)
C (Ox4)
C (Ox2)
C (a)
PPM 140.0 130.0 120.0 110.0 100.0 90.0 80.0 70.0 60.0 50.0 40.0

Figure S17. ^{1}H and ^{13}C NMR spectra of *N*-benzylnaphtho[1,2-*d*]oxazol-8-amine **(19)**

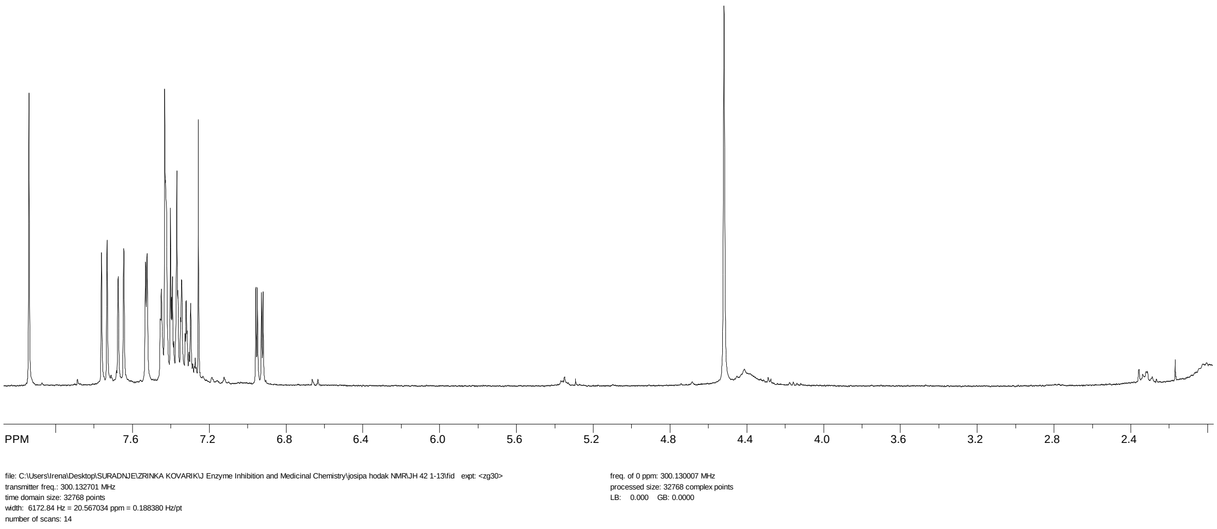

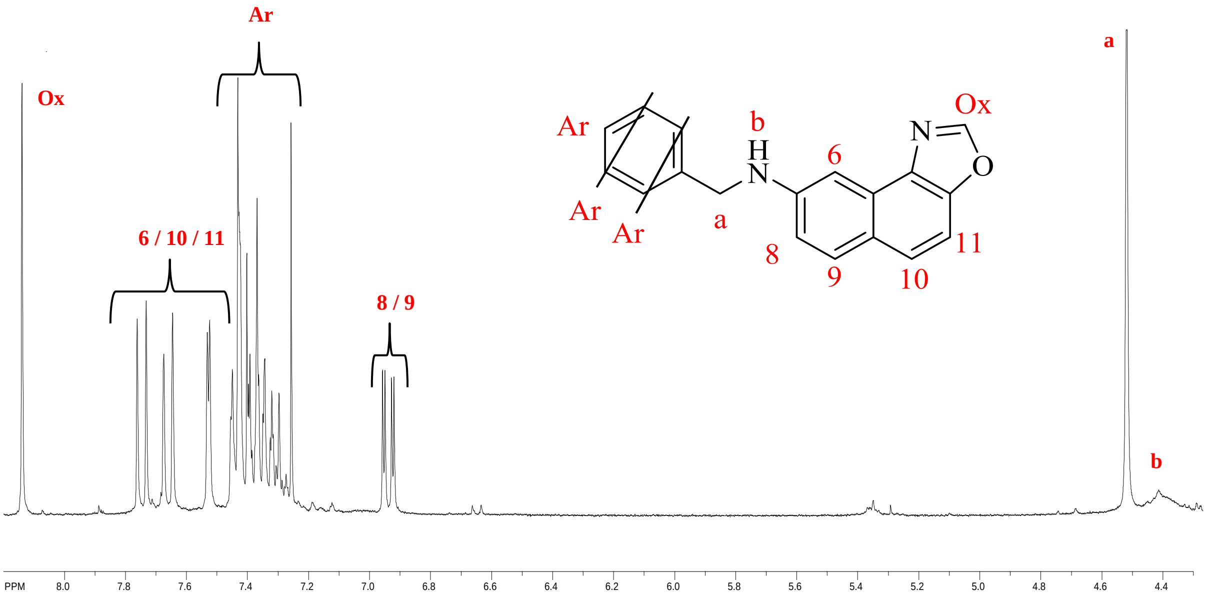

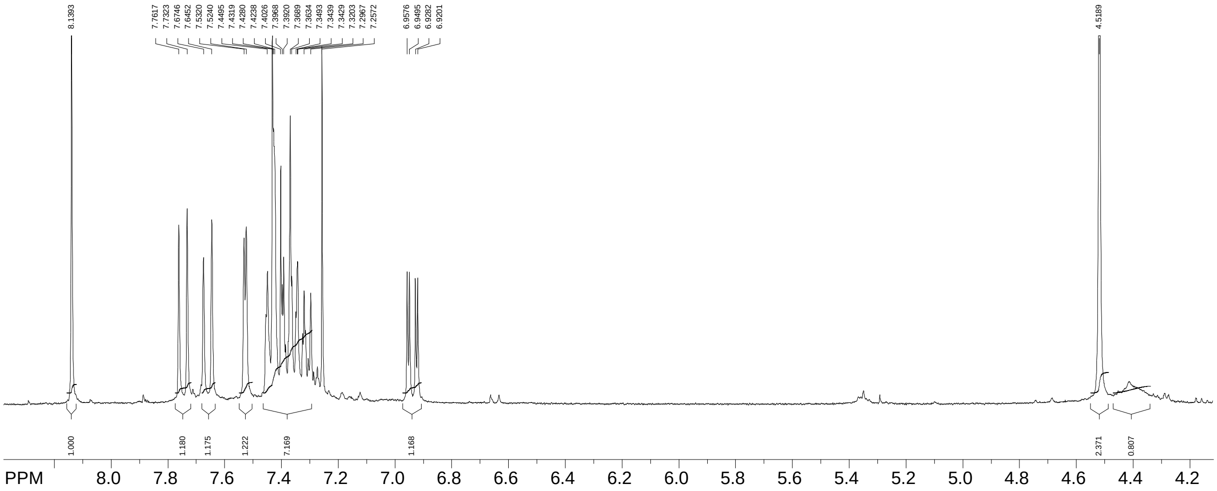
8.1393
7.7617 7.7323 7.6746 7.6452 7.5320 7.5240 7.4495 7.4319 7.4280 7.4238 7.4026 7.3968 7.3920 7.3889 7.3834 7.3493 7.3439 7.3429 7.3203 7.2967 7.2572
6.9576 6.9495 6.9282 6.9201
4.5189
1.000 1.180 1.175 1.222 7.169 1.168 2.371 0.807
PPM 8.0 7.8 7.6 7.4 7.2 7.0 6.8 6.6 6.4 6.2 6.0 5.8 5.6 5.4 5.2 5.0 4.8 4.6 4.4 4.2

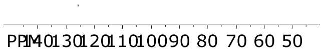
PPM 140 130 120 110 100 90 80 70 60 50

C (a)

C (Ox_2)

C (Ox_4)

Figure S18. ^{1}H and ^{13}C NMR spectra of *N*-(4-fluorobenzyl)naphtho[1,2-*d*]oxazol-8-amine **(20)**

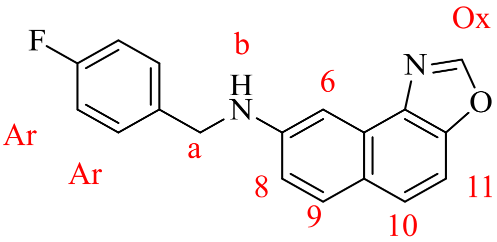

Ar

Ox

8/9

a

6/10/11

b

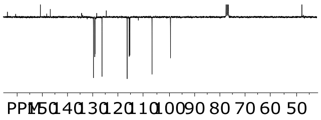

C (a)

C (Ox$_2$)

Figure S19. ¹H NMR spectrum of *N*-(thiophen-2-ylmethyl)naphtho[1,2-*d*]oxazol-8-amine **(21)**

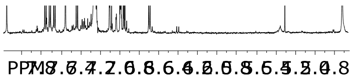

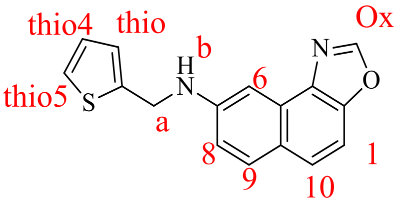

Figure S20. Acetylcholinesterase inhibition by *trans*-amino-5-arylethenyl-oxazole derivatives. Due to the inhibition of AChE by the solvent, DMSO, maximal tested concentration of the compounds was 100 µM and, substrate (acetylthiocholine) concentration was 0.2 mM.

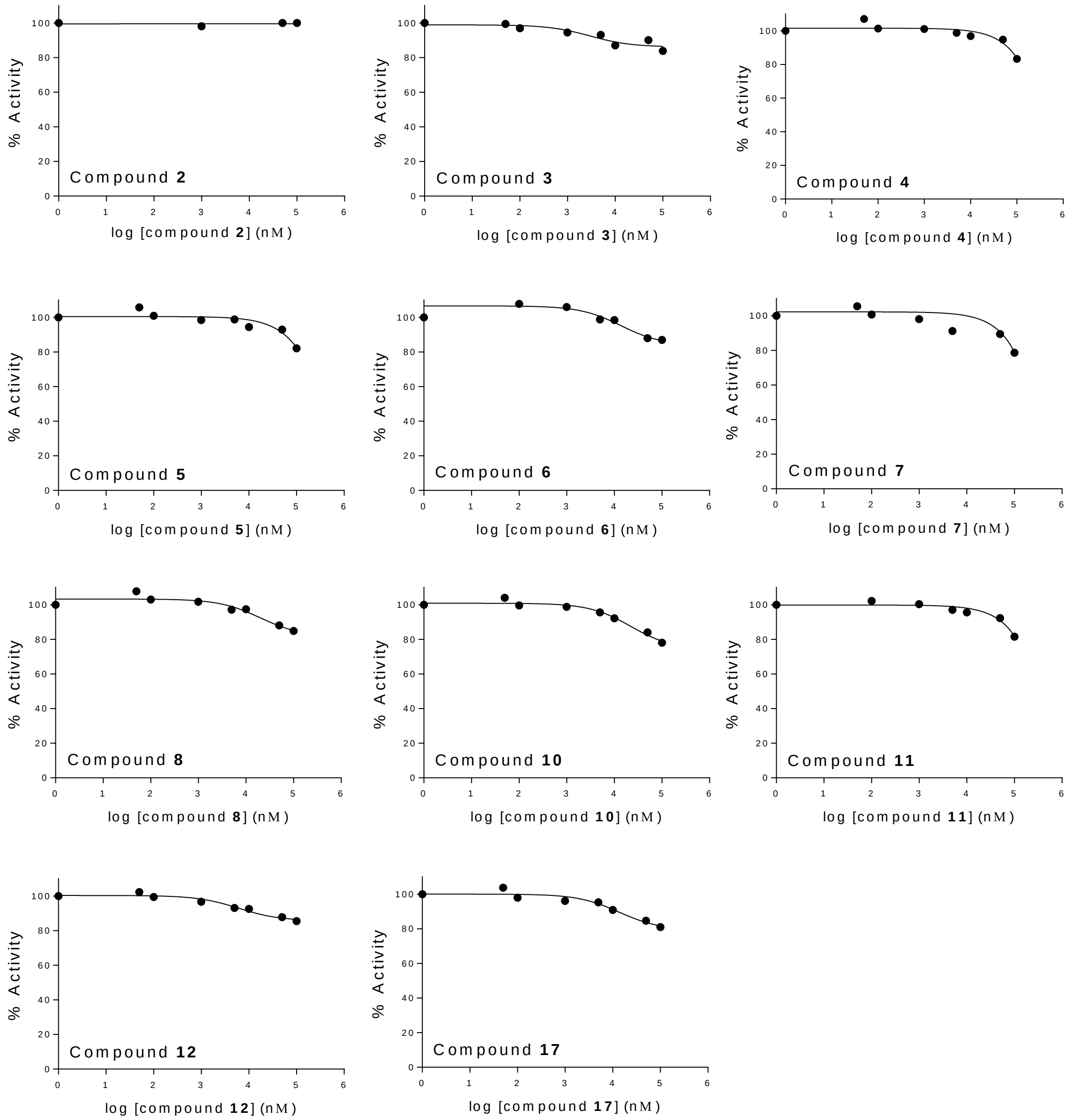

Supplement: Supplemental Material [file IENZ_A_1707197_SM5345.pdf]
